# Sex difference of pre- and post-natal exposure to six developmental neurotoxicants on intellectual abilities: a systematic review and meta-analysis of human studies

**DOI:** 10.1186/s12940-023-01029-z

**Published:** 2023-11-17

**Authors:** Carly V. Goodman, Rivka Green, Allya DaCosta, David Flora, Bruce Lanphear, Christine Till

**Affiliations:** 1 Faculty of Health, York University, Toronto, M3J 1P3 ON Canada; 2 Faculty of Health Sciences, Simon Fraser University, Vancouver, BC Canada

**Keywords:** Developmental Neurotoxicant, IQ, Sex, Prenatal, Postnatal

## Abstract

**Background:**

Early life exposure to lead, mercury, polychlorinated biphenyls (PCBs), polybromide diphenyl ethers (PBDEs), organophosphate pesticides (OPPs), and phthalates have been associated with lowered IQ in children. In some studies, these neurotoxicants impact males and females differently. We aimed to examine the sex-specific effects of exposure to developmental neurotoxicants on intelligence (IQ) in a systematic review and meta-analysis.

**Method:**

We screened abstracts published in PsychINFO and PubMed before December 31st, 2021, for empirical studies of six neurotoxicants (lead, mercury, PCBs, PBDEs, OPPs, and phthalates) that (1) used an individualized biomarker; (2) measured exposure during the prenatal period or before age six; and (3) provided effect estimates on general, nonverbal, and/or verbal IQ by sex. We assessed each study for risk of bias and evaluated the certainty of the evidence using Navigation Guide. We performed separate random effect meta-analyses by sex and timing of exposure with subgroup analyses by neurotoxicant.

**Results:**

Fifty-one studies were included in the systematic review and 20 in the meta-analysis. Prenatal exposure to developmental neurotoxicants was associated with decreased general and nonverbal IQ in males, especially for lead. No significant effects were found for verbal IQ, or postnatal lead exposure and general IQ. Due to the limited number of studies, we were unable to analyze postnatal effects of any of the other neurotoxicants.

**Conclusion:**

During fetal development, males may be more vulnerable than females to general and nonverbal intellectual deficits from neurotoxic exposures, especially from lead. More research is needed to examine the nuanced sex-specific effects found for postnatal exposure to toxic chemicals.

**Supplementary Information:**

The online version contains supplementary material available at 10.1186/s12940-023-01029-z.

## Background

The prevalence of neurodevelopmental disorders (NDDs) is on the rise. From 1997 to 2017, the prevalence of having any one NDD increased from 13 to 18%, especially among males [1]*.* Males have a twofold higher prevalence of NDDs than females due to a complex and dynamic interplay between genetic, hormonal, and environmental factors [2]. The rapid increase in the prevalence of NDDs, especially in males, shows that we need more research on environmental causes to guide policy decisions.

While a variety of environmental factors may contribute to the prevalence of NDDs, mounting evidence suggests that exposure to toxic chemicals during critical periods of development increases children’s risk of NDDs, including intellectual disabilities (ID), attention-deficit/hyperactivity disorder (ADHD), and autism spectrum disorder (ASD) [3–5]. Moreover, experimental and epidemiological studies demonstrate that early-life exposure to toxic chemicals can impact males and females differently [6–8], potentially accounting for sex differences in a variety of NDDs. Nonetheless, the literature remains inconclusive regarding a sex-specific vulnerability. A better understanding of the impact of toxic chemicals on sex-specific differential susceptibility can provide critical insight into the mechanisms underlying risk of NDDs.

The developing brain is susceptible to even low levels of toxic chemicals that might not have an adverse effect on adults [9]. Development is a period of rapid growth when the blood–brain barrier is more permeable and growing cells are more susceptible to toxic chemicals [10]. Thus, as toxic chemicals traverse the blood–brain barrier, they can interfere with sensitive biological processes such as neuronal migration, differentiation, and synaptogenesis [3]. Additionally, fetuses, infants, and young children may have immature metabolic pathways and enzymes to metabolize and excrete toxic chemicals [4].

Unfortunately, pregnant women and infants are exposed to a wide range of chemicals (i.e., developmental neurotoxicants) that can interfere with the developing central nervous system [11–13]. To reduce exposure and harm, Project TENDR (Targeting Environmental Neuro-Development Risks; 2016) – an alliance of more than 50 leading scientists, health.

professionals, and advocates – identified the following as developmental neurotoxicants: lead, mercury, polychlorinated biphenyls (PCBs), polybromide diphenyl ether (PBDE) flame retardants, organophosphate pesticides (OPPs), and phthalates [14]. Each of these developmental neurotoxicants is widespread in North America and has a substantial amount of empirical support indicating that they can cross the placenta and alter brain or endocrine function, even at low levels [14–21].

Developmental neurotoxicants may impact males and females differently [6–8]. Males and females differ in their anatomy, physiology, and biochemistry, all of which can contribute to sex-linked variations in toxicokinetics and toxicodynamics [22, 23]. Sex refers to an individual's physical and biological characteristics that differentiate them as male and female. Males and females may differ in their patterns of exposure as hormonal and social influences shape behaviours, activities, and characteristics [24]. Once a toxic chemical is absorbed via inhalation, ingestion, or dermal absorption, sex differences can also be found in distribution and metabolism. On average, females have greater body fat percentage than males [25, 26]. As a result, females may be more vulnerable to lipophilic chemicals that preferentially accumulate in fat tissue [27]. Moreover, there is evidence for sex differences in the activity of various cytochrome P-450 s (CYP450), the class of enzymes involved in the metabolism of toxic chemicals [28–30], as well as in the activity of glutathione peroxidase, which protects against oxidative damage [31].^.^ Differential activity of detoxification mechanisms can place one sex at heightened vulnerability compared with the other depending on the chemical of exposure.

In recognition of sex-based biological differences, the Institute of Medicine (IOM) published a report in 2001 concluding that sex is a fundamental variable that should be considered at all levels of basic and clinical research [32]. Nevertheless, until 2005, sex was typically used as a confounder in neurotoxicology studies; few studies examined differential effects by sex. In 2005, the Scientific Group on Methodologies for the Safety Evaluation of Chemicals (SGOMSEC) extended the arguments made by the IOM to the field of toxicology [33]. SGOMSEC recommended that future toxicological research should consider sex when designing and analyzing their studies [33].

To determine whether there were any existing systematic reviews or meta-analyses on the sex-specific effects of developmental neurotoxicants, we searched the databases PubMed and PsychINFO for “systematic review” and “meta-analysis” in the title, combined with the search keywords “neurotox*”, “metal*”, “endocrine disrupt*”, “sex”, “gender”, “cognition”, “neurodev”. While some systematic reviews have examined sex-specific neurodevelopmental impacts from heavy metals [6, 8, 34, 35], phthalates [36], and developmental neurotoxicants more broadly [7], they have aggregated data across a variety of endpoints or timing of exposure [6–8, 34]. Aggregating data in such a way can result in vague conclusions and make it difficult to conduct a meta-analysis and quantitatively assess the strength of evidence. Most previous studies have also not considered risk of bias, weighing studies of varying quality equally. Without a better understanding of these sex effects, we may overlook a potentially harmful effect on one sex, especially given the higher rates of NDDs in males. Thus, a systematic review and meta-analysis of the data on sex-specific outcomes from neurotoxic exposure is of high research importance.

The present study aims to examine the sex-specific effects of developmental neurotoxicants in the context of the specific neurotoxicant, the window of exposure, and a specific outcome. IQ is the most studied neurodevelopmental outcome in children [37]. Even mild IQ deficits predict poorer academic and occupational success as well as reduced emotional and physical well-being [38]. Thus, the present study is a systematic review and meta-analysis to determine the sex-specific effects of general, verbal, and nonverbal IQ deficits from pre- and postnatal exposure to six developmental neurotoxicants: lead, mercury, PCBs, PBDEs, OPPs, and phthalates.

## Methods

Our systematic review and meta-analysis was conducted according to the Preferred Reporting Items for Systematic Reviews and Meta-Analysis (PRISMA) statement. The protocol for our review was registered with PROSPERO in 2019 prior to formal screening and was revised in 2020, given changes in our methodology after the pilot screening (CRD42020156526). The changes in methodology and reasons for those changes are in our PROSPERO registration.

### Study question

The search question was: “Among the general population, what are the sex-specific effects of exposure to developmental neurotoxicants (i.e., lead, mercury, PCBs, PBDEs, phthalates, and OPPs) on intellectual abilities?”.

### Search strategy

Titles and abstracts up until December 31st, 2021, were extracted from the electronic databases PubMed and PsycINFO. The search strategy was developed in collaboration with an academic librarian at York University and the collective expertise of review authors (see Table 1). Additional filters were applied to ensure we extracted English, peer-reviewed human studies on children. We also screened the reference lists of included papers to identify any additional studies. The search strategy was first piloted to ensure the sensitivity and specificity in retrieving articles aligned with our PECO statement (changes to our search after pilot testing are in our PROSPERO protocol).

**Table 1:** Example PubMed Search Strategy

No	Search
1	neurotoxic* OR mercury OR PCB OR “polychlorinated biphenyl” OR PBDE OR “polybrominated diphenyl ethers” OR “flame retardants” OR phthalates OR OP OR organophosphates OR pesticides OR lead
2	cogniti* OR neurodev* OR intel* OR IQ
3	#1 AND #2

### Study selection and eligibility criteria

Abstracts were screened by four reviewers (JR, JJ, AD, and CG) and then independently re-reviewed by a fifth reviewer (CG or RG) to determine whether they met the eligibility criteria. Eligibility criteria for Population, Exposure, Comparator, and Outcomes were defined and summarized in a PECO statement (Table 2) [39].

**Table 2:** PECO Statement

Population	Humans of age 3–17 years at time of IQ test with toxicant exposure measured during the prenatal or early postnatal period (up to age six);
Exposure	Exposure to one of the six neurotoxicants (lead, mercury, PCBs, PBDEs, OPPs, and phthalates; level of toxicant exposure was determined through an individualized biomarker (e.g., blood, hair, urine)
Comparator	Children with lower levels of lead, mercury, PCBs, PBDEs, OPPS, or phthalates
Outcome	IQ measured in individual children at ages 3–17 years. IQ assessments include (but are not limited to): Wechsler Preschool and Primary Scale of Intelligence (WPPSI), Wechsler Intelligence Scale for Children (WISC), Wechsler Abbreviated Scale of Intelligence (WASI), Stanford-Binet Intelligence Scale, and the McCarthy Scales of Children's Abilities (MSCA)
Study Design	Empirical epidemiological studies excluding case studies

A full-text review was then independently carried out by three reviewers (CG, RG, and AD) for all study abstracts that met inclusion criteria. Included in the analysis were studies that [1] evaluated general, verbal, and/or nonverbal IQ and [2] reported the interaction by sex (i.e., interaction coefficient/*p*-value) or effects separately by sex. Sex was defined as an individual's physical and biological characteristics that differentiate them as male or female. A full-text review was also carried out for study abstracts in which there was ambiguity concerning whether they met the inclusion criteria or in which no abstract was available.

### Data extraction

Two reviewers (CG and one of JJ, JR, RM, NS, and AD) independently extracted data on authors, publication year, study aim, design, population, sample size, exposure characteristics (toxicant, timing of exposure, biomarker, mean of exposure), outcome characteristics (IQ test, and age at IQ test), and results (regression coefficients, standard errors, p-values), using a standardized extraction form in Covidence (a systematic review software tool). A third reviewer (RG) settled any discrepancies. If a study was missing quantitative data, its corresponding author was contacted. If the author did not respond after three attempts of contact over the course of a two-month period, the data were considered unretrievable.

### Risk of bias

We evaluated risk of bias using Navigation Guide, a systematic review methodology developed by Woodruff and Sutton [43]Click or tap here to enter text.. This systematic review tool was designed to evaluate evidence relating environmental exposures to adverse health outcomes and has been recommended for use over similar tools [40]. Nine areas were assessed for risk of bias: selection bias, blinding, confounding, exposure, outcome, incomplete outcome data, selective outcome reporting, conflict of interest, and other threats to internal validity. Each area could be rated as “low”, “probably low”, “probably high”, or “high” risk of bias. Instructions for making risk of bias determinations were modified from Lam and colleagues’ systematic review [20] (Additional file 1: Appendix A). Two reviewers (CG and one of JJ, JR, RM, NS, and AD) independently made risk of bias determinations for each study and all discrepancies were resolved by a third reviewer (RG).

### Certainty of the evidence

#### Quality

We rated the overall quality of the evidence for each grouping in our meta-analysis as "high," "moderate," or "low." Initial classification of human observational studies was set at "moderate" quality [41]. Subsequently, we considered modifications ("decreases" or "increases") in the quality rating, taking into account eight different aspects: bias risk, indirectness, inconsistency, imprecision, the likelihood of publication bias, considerable effect size, dose–response, and the potential of residual confounding to lessen the overall impact estimate [42]. Guidelines for reviewers were based on those outlined in the Navigation Guide methodology protocol [43]. The rating possibilities consisted of 0 (no deviation from initial quality rating), − 1 (single level decrease), -2 (double level decrease), + 1 (single level increase), or + 2 (double level increase).

#### Strength

We rated the overall strength of the evidence for each grouping in our meta-analyses (i.e., associations between prenatal exposure and general, verbal, and nonverbal intelligence, as well as associations between postnatal lead exposure and general intelligence in males and females) based on four considerations: quality of body of evidence; direction of effect; confidence in effect; and other characteristics of the data that may influence certainty. Possible ratings were “sufficient evidence of toxicity,” “limited evidence of toxicity,” “inadequate evidence of toxicity,” or “evidence of lack of toxicity”, based on guidelines from Navigation Guide [43].

All study authors contributed to assessing the overall quality and strength of the evidence. Discrepancies were discussed until consensus was reached.

### Evidence synthesis

#### Quantitative synthesis

##### Studies suitable for quantitative synthesis

We identified studies suitable for quantitative synthesis based on the study features, exposure assessment, outcome assessment, and method of data analysis (i.e., linear regression techniques). We considered studies that measured the exposure at any point during pregnancy or at birth as combinable measures of prenatal exposure. We considered studies that measured the exposure at any point from infancy to age six years as combinable measures of postnatal exposure. When regression coefficients were available for groups of chemicals, for instance, PBDE, PCB, phthalate, and OPP congeners, we considered studies that evaluated the sum of exposures (i.e., ΣDEHPs for phthalates, ΣPCBs for PCBs, ΣBDEs for PBDEs, and ΣDAPs for OPPs) as combinable.

We considered Full-Scale IQ (FSIQ) evaluated by any Wechsler test and the General Cognitive Index (GCI) evaluated by the McCarthy Scale of Children’s Abilities (MSCA) as combinable measures of general intelligence [44]. We considered verbal IQ (VIQ), or the Verbal Comprehension Index (VCI) evaluated by any Wechsler test and the Verbal Scale evaluated by the MSCA as combinable measures of verbal intelligence. We considered performance IQ (PIQ) or the Perceptual Reasoning Index (PRI) evaluated by any Wechsler test, the Perceptual-Performance Scale (PPS) evaluated by the MSCA, and the Snijders-Oomen Non-Verbal Intelligence Test (SON-R) as combinable measures of nonverbal intelligence [45]. Since Weschler, MSCA, and SON-R tests are standardized with mean scores of 100 and a standard deviation of 15, they were not rescaled.

When multiple effect sizes (i.e., Beta coefficients) were available from the same study or sample (e.g., multiple exposure measurements or outcome measurements), effect sizes based on the methodologies that most closely resembled the methodologies of the other included studies in the meta-analysis were selected to minimize heterogeneity among studies. If the same prospective cohort study included data on more than one neurotoxicant, they were considered as separate effects.

##### Effect size transformation

To homogenize the magnitude of effect observed in each study, results were recalculated as an absolute change in the dependent variable (i.e., IQ) for a relative difference of k = 1.5 times in the exposure variable (i.e., a 50% difference; see Additional file 1: Appendix B for additional information) [46]. For studies that did not report a standard error or confidence interval, we calculated the standard error as a function of the t-statistic for the regression coefficient of the exposure.

##### Meta analysis

We ran separate random effects meta-analyses using the DerSimonian-Laird method [47] examining the male- and female-specific effects of prenatal exposures on general, nonverbal, and verbal intelligence. Further, given that each neurotoxicant may differ in its route of exposure and toxicological effects, we also ran subgroup analyses by neurotoxicant, for those with at least two studies. Due to the limited number of studies available, we did not perform a separate analysis of PCBs for any intelligence outcome, nor separate analyses of OPPs or PBDEs for verbal intelligence.

Moreover, we ran separate random effects meta-analyses of the male- and female-specific effects of postnatal lead exposure on general intelligence. Due to the limited number of studies (i.e., fewer than two per neurotoxicant), we were unable to analyze postnatal effects of any of the other neurotoxicants. The results from the meta-analyses are graphed with forest plots.

##### Heterogeneity and publication bias

We assessed the heterogeneity of studies using the τ^2^, I^2^ and H^2^ statistics [48]. We used Egger’s Test to assess potential publication bias via funnel plot asymmetry [49].

##### Sensitivity analyses

To examine the influence of each study on the overall effect-size estimate, we used the leave-one-out method whereby the overall effect size was re-computed from a meta-analysis excluding one study at a time [50]. Moreover, we ran additional meta-analyses in which only included studies rated as “low” or “probably” low risk of bias across all areas.

All statistical analyses were run using Stata 17.0 (Stata Corporation, College Station, TX, U.S.A.). A *p*-value < 0.05 was considered statistically significant for all analyses in our study.

##### Narrative synthesis

Studies not included in the meta-analysis were narratively described in a table with data on the country/cohort, biomarker, mean of exposure, outcome measure, and a general interpretation of the study findings. Studies identified as “low” or “probably low” risk of bias across all nine areas were organized into meaningful groups based on our PECO components and then narratively described based on these same groupings [i.e., by timing (pre- or post-natal) and outcome measure (general, nonverbal, or verbal IQ)]. Given the limited number of studies in each grouping with data on each developmental neurotoxicant, we did not further subdivide these studies by neurotoxicant.

## Results

### Study selection

**Fig. 1:**
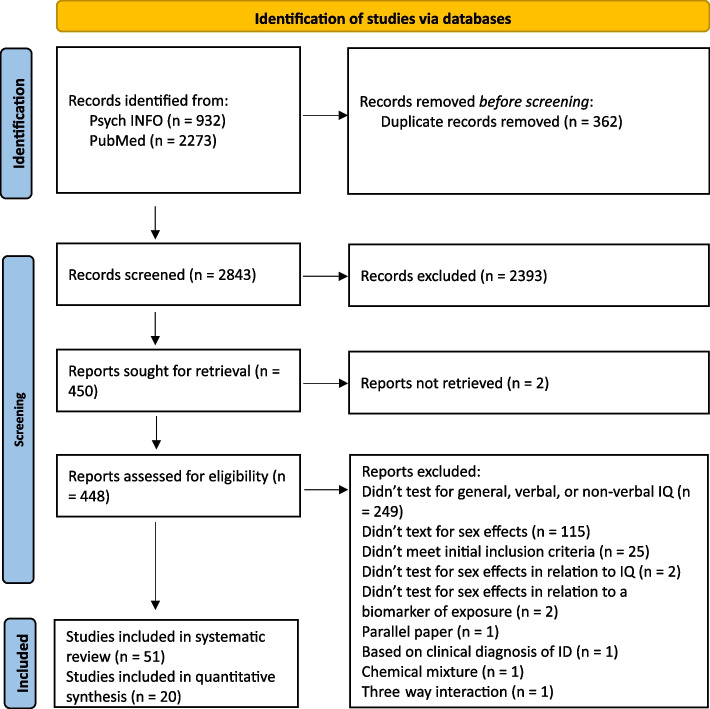
PRISMA 2020 Flow Diagram

The PRISMA 2020 flow diagram is shown in Fig. 1. Our search retrieved 2843 unique articles, of which 450 met the initial inclusion criteria and were included in the full-text review. Fifty-one articles fulfilled the full-text inclusion criteria and were included in the systematic review [51–102]. Of the 51 studies included in the systematic review, 17 pertained to lead, 13 mercury, nine phthalates, six OPPs, five PCBs, and five PBDEs. Four articles included data from exposure to more than one neurotoxicant, thus data on each neurotoxicant was included in the systematic review^86−89^. Of the 51 studies included in the systematic review, 20 were included in the meta-analyses [53–55, 57, 63, 64, 66, 67, 69, 73, 76, 79, 80, 83, 88, 90, 95–97, 97, 99, 101].

### Meta-analysis study characteristics

We tabulated the characteristics of the studies in the meta-analysis, including the country/cohort, biomarker, mean of exposure in parts per billion (ppb), and the outcome measure (Table 3).

**Table 3:** Meta-analysis study characteristics

First Author, Year	Country (Cohort)	N (%_males_)	Neurotoxicant	Compound	Biomarker and Timing of Exposure	Median of Exposure (ppb)	Outcome Measure
Azar, 2021 [55]	Canada (MIREC)	584–589 (48.8–49.1)	PBDE	ΣPBDE^c^	1^st^ trimester blood	12.9	FSIQ and PIQ on WPPSI-III at 3–4 years
Bouchard, 2011 [57]	USA (CHAMACOS)	297 (47.5)	OPP	∑DAP^d^	Mean of 5–27 weeks’ gestation urine and 18–39 weeks’ gestation urine	41.3	FSIQ on WISC-IV at 7 years
Desrochers-Couture, 2018 [63]	Canada (MIREC)	601–606 (~ 48.8)	Lead Lead	N/A N/A	Cord blood Child blood at 3–4 years	7.9 6.7	FSIQ, PIQ, and VIQ on WPPSI-III at 3–4 years
Dietrich, 1993 [64]	USA (Cincinnati Lead Study)	251 (N/A)	Lead	N/A	Cord Blood	83.0^a^	PIQ on WISC-R at 6.5 years
Eskenazi, 2013 [66]	USA (CHAMACOS)	231–256 (45.5–45.7)	PBDE	ΣPBDE^c^	2^nd^ trimester blood or maternal blood at delivery	24.9	PIQ on WPPSI-III at 5 years and FSIQ on WISC-IV at 7 years
Gascon, 2015 [69]	Spain (INMA- Sabadell)	367 (52.0)	Phthalate	ΣDEHP^e^	Average of 1^st^ and 3^rd^ trimester urine	99,800	GCI, PPS, VS on MSCA at 4 years
Guo, 2020 [53]	China (SMBCS)	297 (~ 57.0)	Lead Mercury	N/A	Maternal urine at delivery Maternal urine at delivery	1.7 0.4	FSIQ, PRI, and VCI, on C-WISC at 7 years
Huang, 2012 [101]	Taiwan (N/A)	133 (~ 51.9)	Lead	N/A	Blood at 2–3 years Blood at 5–6 years	25.0 23.0	FSIQ on WPPSI-R at 5–6 years and WISC-III at 8–9 years
Hyland, 2019 [73]	USA (CHAMACOS)	321–323 (47.9- 48.5)	Phthalate	ΣDEHP^e^	Median of 13- and 26-week urine	78.1	FSIQ, PRI, and VCI on WISC-IV at 7 years and 10.5 years
Julvez, 2013 [76]	UK (ALSPAC)	914 (52.4)	Mercury	N/A	Umbilical cord tissue	26.0^a^	FSIQ on WISC-III short form at 8 years
Jusko, 2019 [77]	Netherlands (Generation R)	708 (51.3)	OPP	∑DAP^d^	Mean of three urines	~ 314 nmol/g	Mosaics and Categories subtests on SON-R at 6 years
Li, 2019 [79]	USA (HOME)	245–251 (~ 43.0–45.0)	Phthalate	ΣDEHP^f^	26 weeks’ gestation urine	69.8	FSIQ on WPPSI-III at 5 years or WISC-IV at 8 years
Llop, 2017 [80]	Spain (INMA)	1362 (52.3)	Mercury	N/A	Cord blood	8.80^a^	GCI, PPS, VS on MSCA at 4 years
McMichael, 1992 [83]	Australia (Port Pirie Cohort Study)	548 (N/A)	Lead	N/A	Lifetime average blood up to 4 years	~ 190^a^	GCI on MSCA at 4 years
Ntantu Nkinsa, 2020 [88]	Canada (MIREC)	505–510 (48.5–48.8)	OPP	∑DAP^d^	1^st^ trimester urine	27.8	FSIQ and PIQ on WPPSI-III at 3–4 years
Pocock, 1987 [90]	UK (The Institute of Child Health/Southampton Study)	402 (45.3)	Lead	N/A	Incisor teeth at 6 years	Males: 3980^b^ Females: 4020^b^	FSIQ on WISC-R at 6 years
Tatsuta, 2020 [54]	Japan (TSCD)	289 (51.2)	Lead Mercury	N/A	Cord blood Cord blood	8.0 ~ 15.6	FSIQ, PRI, and VCI on Japanese version of the WISC-IV at 12 years
Taylor, 2017 [95]	UK (ALSPAC)	1826 (56.6)	Lead	N/A	1^st^ trimester blood	34.1	FSIQ, PIQ, and VIQ on WISC-III (UK) at 8 years
Torres-Olascoaga, 2020 [96]	Mexico (ELEMENT)	188–214 (~ 46.8)	Phthalate	ΣDEHP^e^	2^nd^ trimester urine	N/A	GCI on MSCA at 4 years
van den Dries, 2020	Netherlands (Generation R)	1269–1274 (~ 50.5)	Phthalate	ΣDEHP^e^	Mean of three urines	~ 0.042	Mosaics and Categories subtests on SON-R at 6 years

^a^Arithmetic mean, ^b^geometric mean

^c^ΣPBDE = BDE-47 + BDE-99 + BDE-100 + BDE-153; ^d^ΣDAP = DMP + DMTP + DMDTP + DEP + DETP + DEDTP; ^e^ΣDEHP = MEHHP + MEHP + MEOHP + MECPP; ^f^ΣDEHP = MEOHP + MEHHP + MECPP

*Abbreviations: ALSPAC* Avon Longitudinal Study of Parents and Children, *BDE* Brominated Diphenyl Ethers, *CCCEH* Columbia Center for Children's Environmental Health, *CHAMACOS* Center for the Health Assessment of Mothers and Children of Salinas, *C-WISC* Chinese Version of the Wechsler Intelligence Scale for Children, *DAP* Dialkyl Phosphates, *DEDTP* Diethyl Dithiophosphate, *DEHP* Bis(2-ethylhexyl) Phthalate, *DEP* Diethyl phosphate, *DETP* Diethyl Thiophosphate, *DMDTP* Dimethyl Dithiophosphate, *DMP* Dimethyl Phosphate, *DMTP* Dimethyl Thiophosphate, *ELEMENT* Early Life Exposures in Mexico to ENvironmental Toxicants, *FSIQ* Full-Scale IQ, *GCI* General Cognitive Index, *HOME* Health Outcomes and Measures of the Environment, *INMA* INfancia y Medio Ambiente (Environment and Childhood) Project, *IQ* Intelligence Quotient, *MABC*: The Ma'anshan Birth Cohort, *MBzP* Monobenzyl Phthalate, *MECPP* Mono(2-ethyl-5-carboxypentyl) Phthalate, *MEHHP* Mono(2-ethyl-5-hydroxyhexyl) Phthalate, *MEHP* Mono(2-ethylhexyl) Phthalate, *MEOHP* Mono-(2-ethyl-5-oxohexyl) Phthalate, *MEP* Mono-ethyl Phthalate, *MiBP* Mono-isobutyl Phthalate, *MIREC* Maternal-Infant Research on Environmental Chemicals, *MnBP* Mono-n-butyl Phthalate, *MSCA* McCarthy Scales of Children's Abilities, *OPP* Organophosphate Pesticides, *PBDE* Polybrominated Diphenyl Ethers, *PCB* Polychlorinated Biphenyls, *PIQ* Performance Intelligence, *PPS* Perceptual Performance Scale, *PRI* Perceptual Reasoning Index, *SMBCS* Sheyang Mini Birth Cohort Study, *SON-R* Snijders-Oomen Nonverbal Intelligence Test, *TSCD* Tohoku Study of Child Development, *UK* United Kingdom, *USA* United States of America, *VCI* Verbal Comprehension Index, *VIQ* Verbal Intelligence, *VS* Verbal Scale, *VSI* Visual Spatial Index, *WISC-III* Wechsler Intelligence Scale for Children, Third Edition, *WISC-IV* Wechsler Intelligence Scale for Children, Fourth Edition, *WISC-R* Wechsler Intelligence Scale for Children, Revised, *WPPSI-III* Wechsler Preschool & Primary Scale of Intelligence, Third Edition, *WPPSI-R* Wechsler Preschool & Primary Scale of Intelligence, Revised

Included studies were published between 1987 and 2021 and involved between 188 and 1827 study participants from 14 different cohorts around the world. Nine studies were conducted in North America, seven in Europe, one in Australia, and three in Asia. Seven studies pertained to lead, four to mercury, two to PBDEs, three to OPPs, and five to phthalates. Studies measured neurotoxicant exposure in urine (*n* = 9), blood (*n* = 9), incisor teeth (*n* = 1) or umbilical cord tissue (*n* = 1). Sixteen studies measured toxicant exposure during the prenatal period, three measured toxicant exposure during the postnatal period, and one measured toxicant exposure during both the prenatal and postnatal period. Among the 20 studies included, 14 assessed intelligence using a Wechsler test. Nineteen studies examined general intelligence, 14 examined nonverbal intelligence, and ten examined verbal intelligence.

We rated the risk of bias for the studies in the meta-analysis (Table 4). Nine studies were rated as “low” or “probably low” risk of bias in all areas assessed. Eleven studies were rated as “high” or “probably high” risk of bias in one or more area. Many of the studies that had at least one rating of “probably high” risk of bias were rated as such in the area of confounding. Our rationale for each study’s rating across each area is provided in Supplementary Tables 1–20.

**Table 4:**
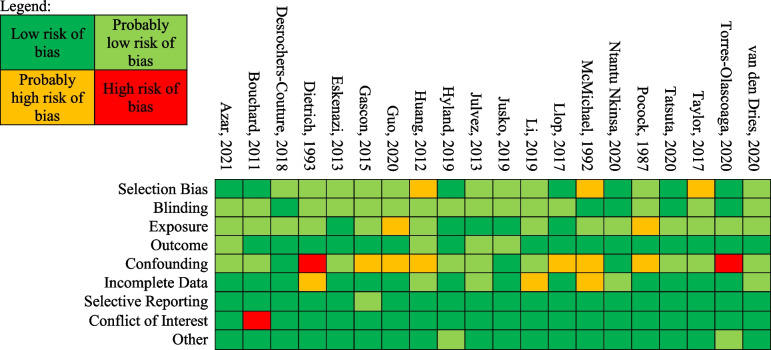
Risk of Bias Ratings for Studies Included in the Meta-Analysis

### Prenatal exposures

#### General intelligence

The meta-analysis of prenatal exposures and general intelligence included 14 studies: four lead [53, 54, 63, 95], four mercury [53, 54, 76, 80], two PBDEs [55, 66], two OPPs [57, 88], and four phthalates [69, 73, 79, 96]. Two articles included data from exposure to more than one neurotoxicant (i.e., lead and mercury), thus data on each neurotoxicant were included in the meta-analysis [53, 54].

**Fig. 2:**
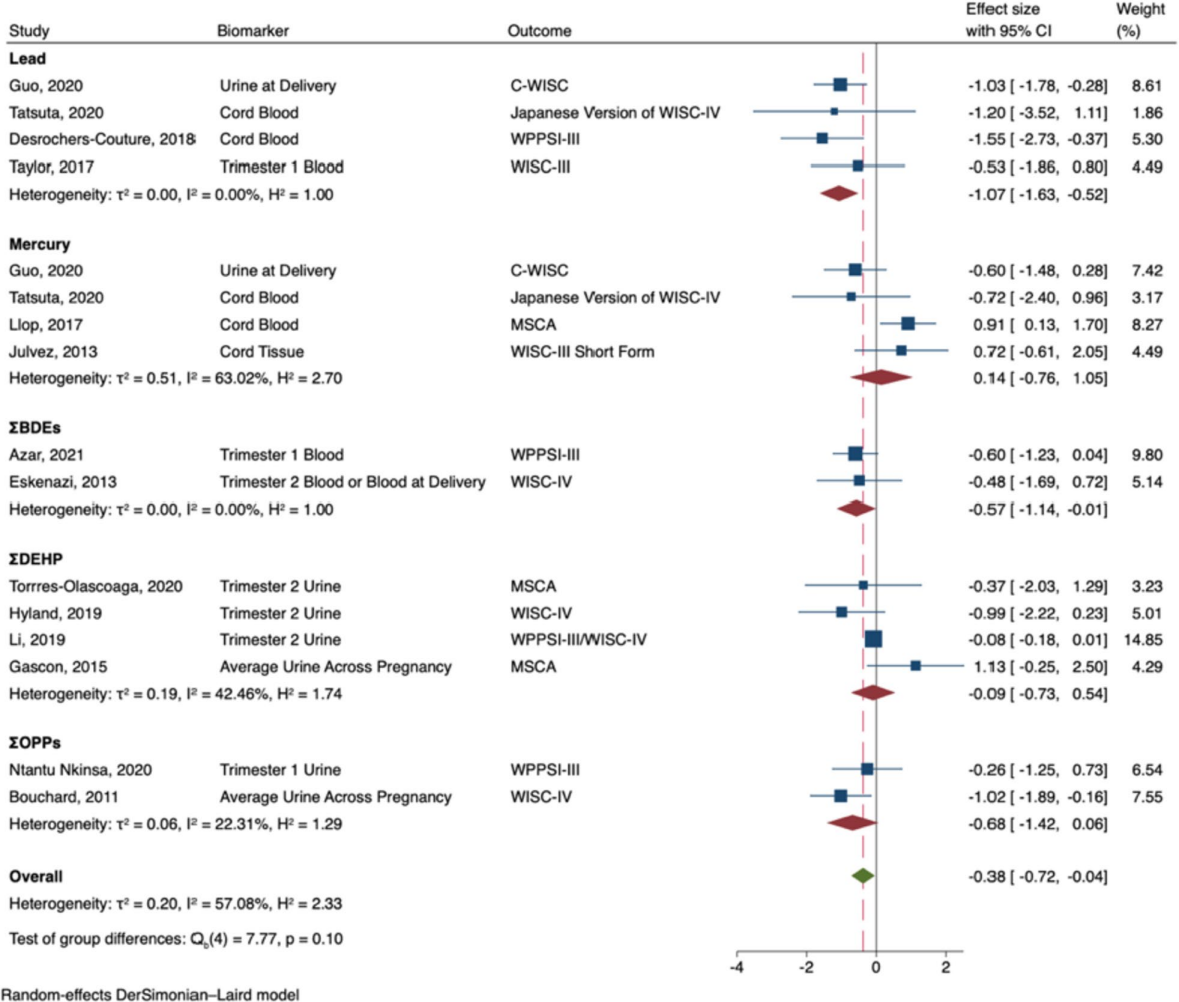
Random-Effects Meta-Analysis—General Intelligence in Males (The range of beta coefficient values is on the X-axis and the included references are listed on the Y-axis. The blue boxes correspond to each reference's beta coefficient and the size of the blue box represents the weight of the study in the meta-analysis, based on its’ standard error. The error bars on each box represent the upper and lower 95% confidence intervals. The subgroup pooled estimates are each provided by red diamonds. The overall meta-analysis pooled estimate is provided by a green diamond and a vertical dashed red line. The ‘line of no effect’ (i.e., the line at which the beta coefficient equals 0) is represented by a black vertical line)

**Fig. 3:**
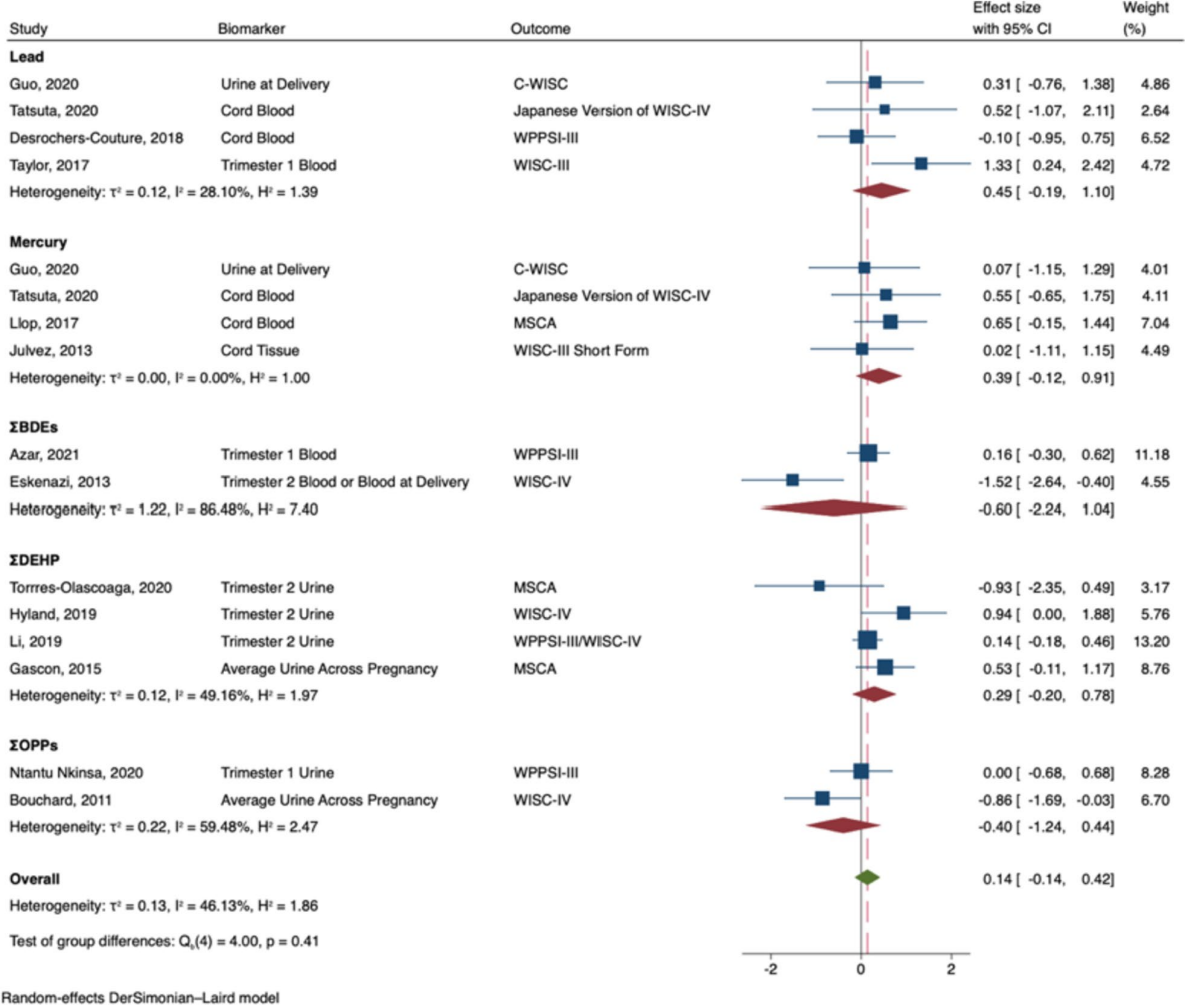
Random-Effects Meta-Analysis—General Intelligence in Females

Prenatal exposure to developmental neurotoxicants was associated with decreased general intelligence in males (B = -0.38; 95% CI -0.72, -0.04; I^2^ = 57%; see Fig. 2). In subgroup analyses, prenatal exposure to lead (B = -1.07; 95% CI: -1.63, -0.52), and ΣPBDEs (B = -0.57; 95% CI: -1.14, -0.01) were significantly associated with decreased general intelligence in males. Nonetheless, the effects did not significantly differ by neurotoxicant (Q_b_ = 7.77, p = 0.10), and effect sizes were largely negative across neurotoxicants. In contrast, prenatal exposure to developmental neurotoxicants was not significantly associated with general intelligence in females (B = 0.14; 95% CI: -0.14, 0.42; I^2^ = 46%; see Fig. 3). Effect sizes were either largely near zero or slightly positive, regardless of the neurotoxicant (Q_b_ = 4.00, p = 0.41).

#### Nonverbal intelligence

The meta-analysis of prenatal exposures and nonverbal intelligence included 13 studies: five lead [53, 54, 63, 64, 95], three mercury [53, 54, 80], two PBDEs [55, 66], three phthalates [69, 73, 97], and two OPPs [77, 88]. Two articles included data from exposure to more than one neurotoxicant (i.e., lead and mercury), so we included results for each neurotoxicant in the meta-analysis [53, 54].

**Fig. 4:**
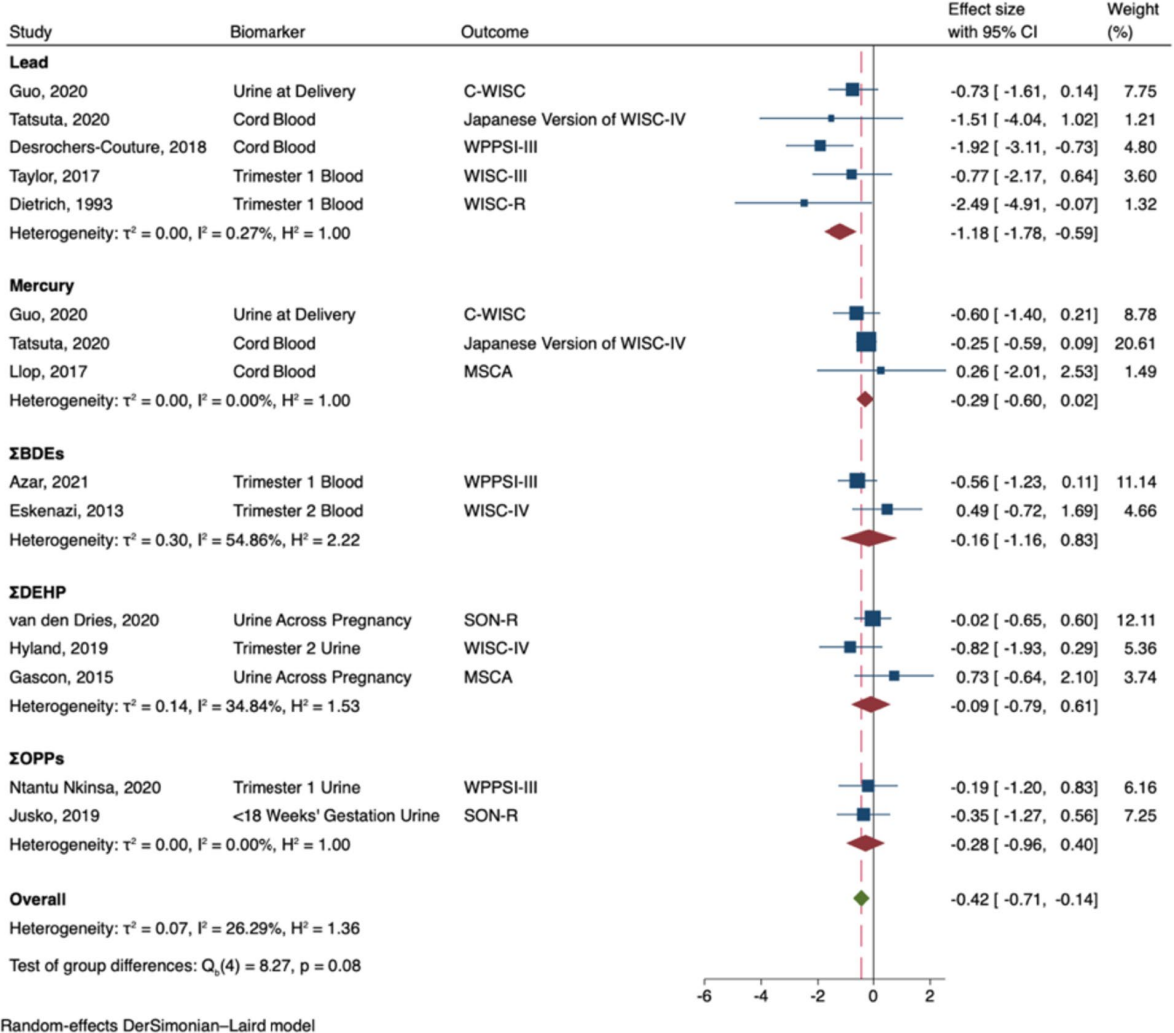
Random-Effects Meta-Analysis—Nonverbal Intelligence in Males

**Fig. 5:**
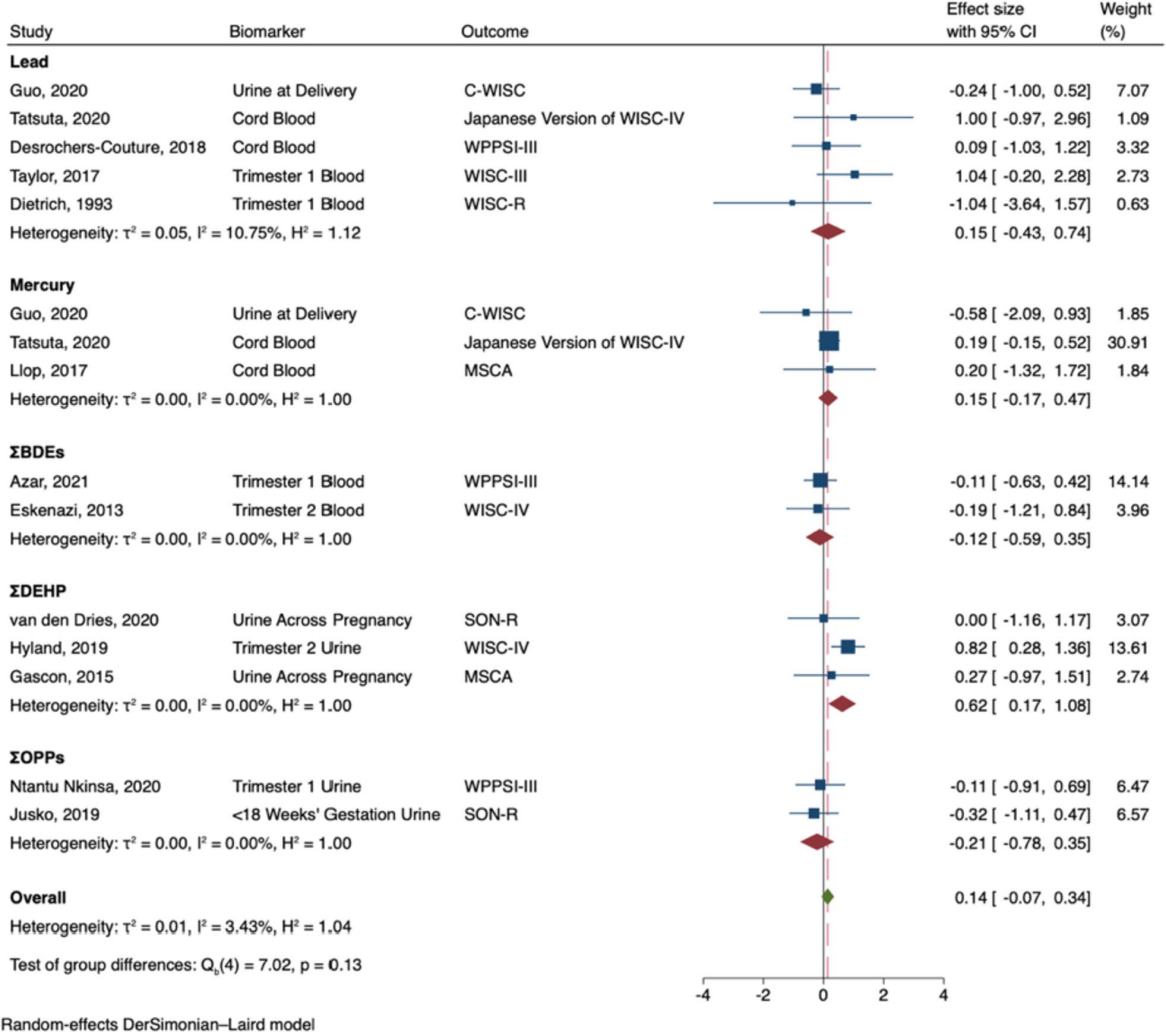
Random-Effects Meta-Analysis—Nonverbal Intelligence in Females

Prenatal exposure to developmental neurotoxicants was associated with decreased nonverbal intelligence in males (B = -0.42; 95% CI: -0.71, -0.14; I^2^ = 26%; see Fig. 4). In subgroup analyses, prenatal exposure to lead (B = -1.18; 95% CI: -1.78, -0.59) was significantly associated with decreased nonverbal intelligence. Nonetheless, effect sizes were largely negative across the neurotoxicants, and the effects did not significantly vary by neurotoxicant (Q_b_ = 8.27, p = 0.08). In contrast, prenatal exposure to developmental neurotoxicants was not significantly associated with nonverbal intelligence in females (B = 0.14; 95% CI: -0.07, 0.34; I^2^ = 3%; see Fig. 5). In subgroup analyses, prenatal exposure to ΣDEHP (B = 0.62; 95% CI: 0.17, 1.08) was significantly associated with greater nonverbal intelligence in females; however, effect sizes did not significantly vary by neurotoxicant (Q_b_ = 7.02, p = 0.13).

#### Verbal intelligence

**Fig. 6:**
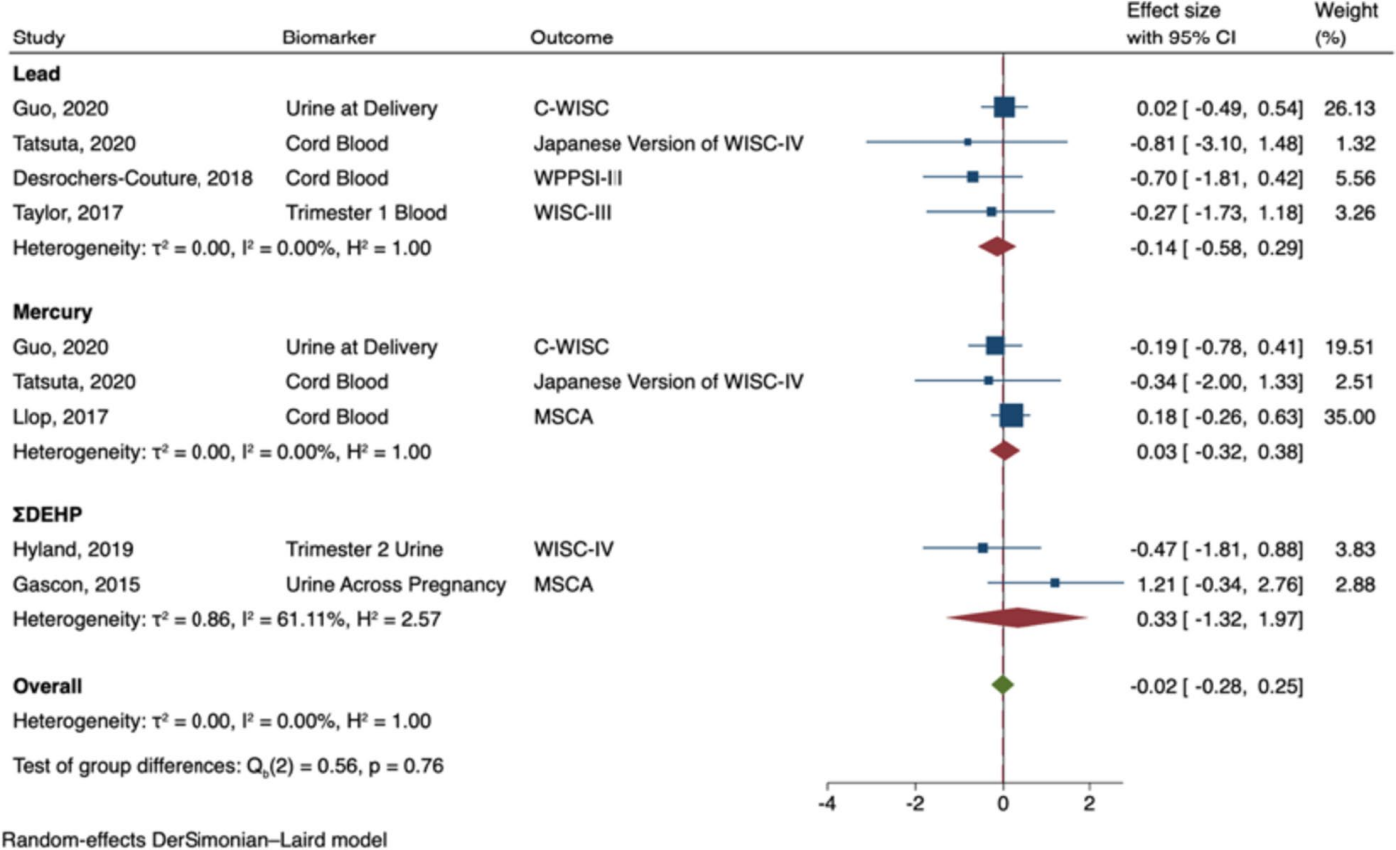
Random-Effects Meta-Analysis—Verbal Intelligence in Males

**Fig. 7:**
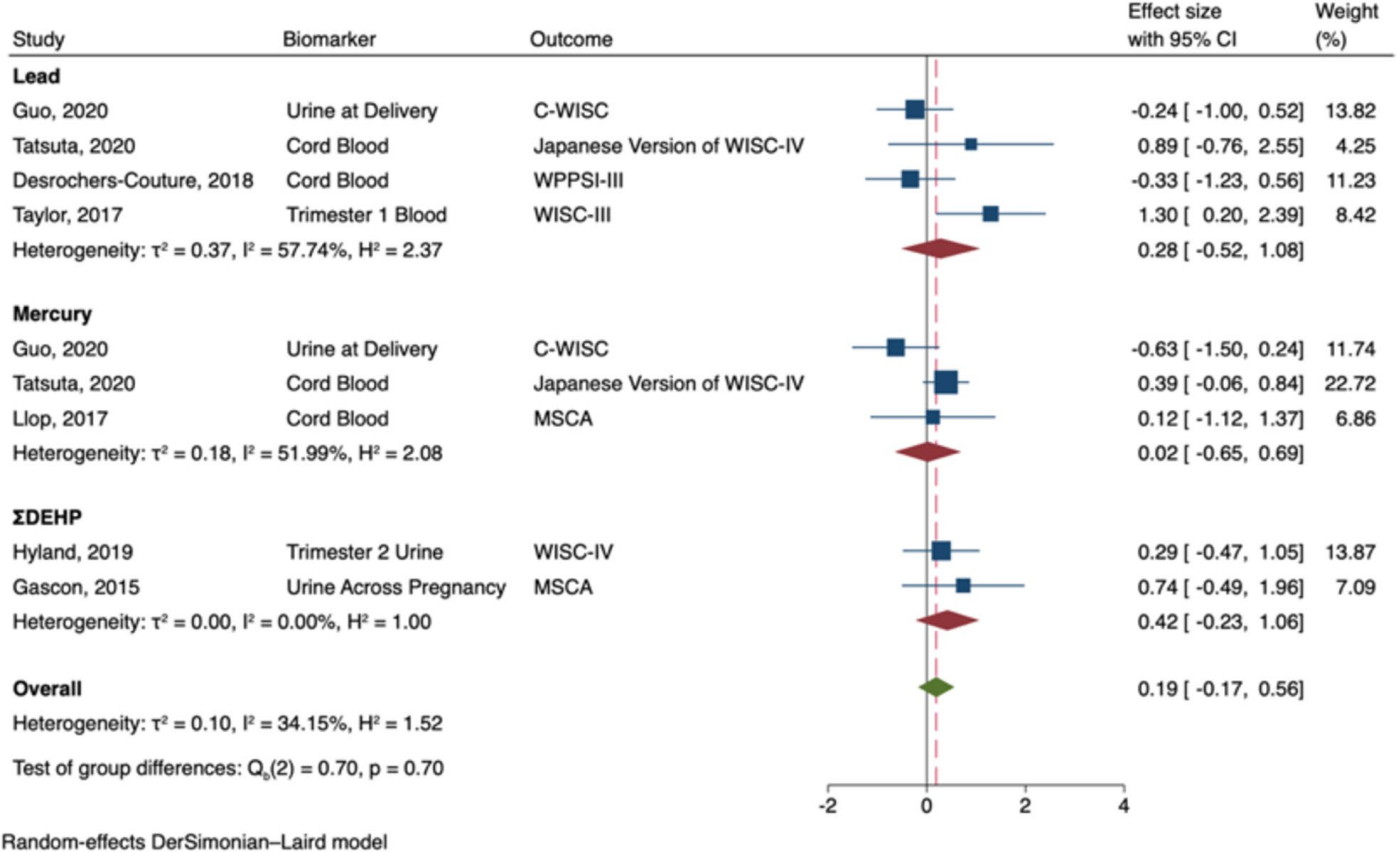
Random-Effects Meta-Analysis—Verbal Intelligence in Females

The meta-analysis of prenatal exposures and verbal intelligence included seven studies: four pertaining to lead [53, 54, 63, 95], three mercury [53, 54, 80], and two phthalates [69, 73]. Two articles included data from exposure to more than one neurotoxicant (i.e., lead and mercury) so data on each neurotoxicant was included in the meta-analysis [53, 54]. Prenatal exposure to developmental neurotoxicants was not significantly associated with verbal intelligence in males (B = -0.02; 95% CI: -0.28, 0.25; I^2^ = 0%; see Fig. 6), nor in females (B = 0.19; 95% CI: -0.17, 0.56; I^2^ = 34%; see Fig. 7). Effect sizes did not significantly vary by neurotoxicant in either males or females (Q_b_ males = 0.56, *p* = 0.76; Q_b_ females = 0.70, *p* = 0.70).

### Postnatal exposure

#### Lead and general intelligence

**Fig. 8:**
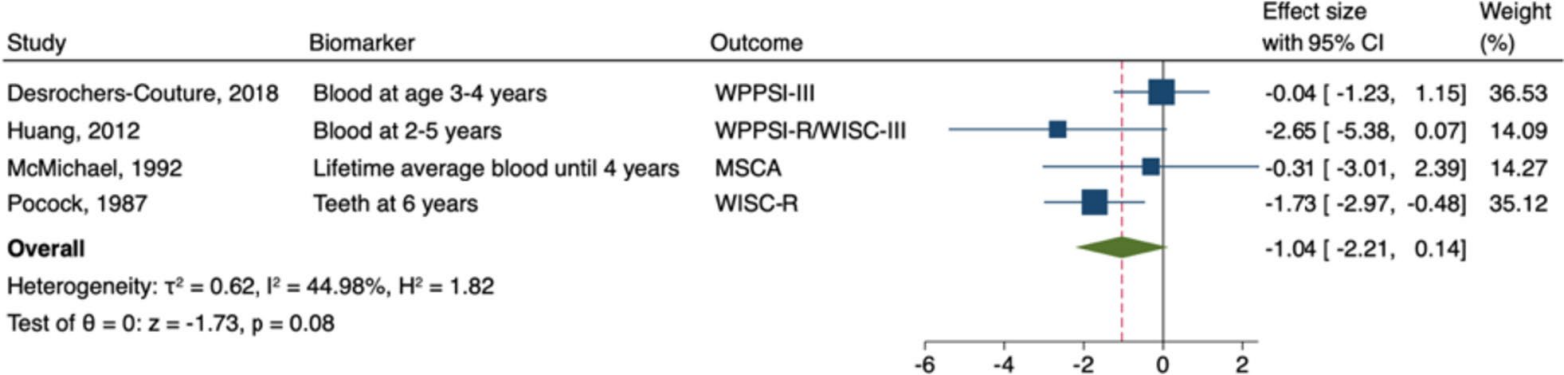
Random-Effects Meta-Analysis—Postnatal Lead and General Intelligence in Males

**Fig. 9:**
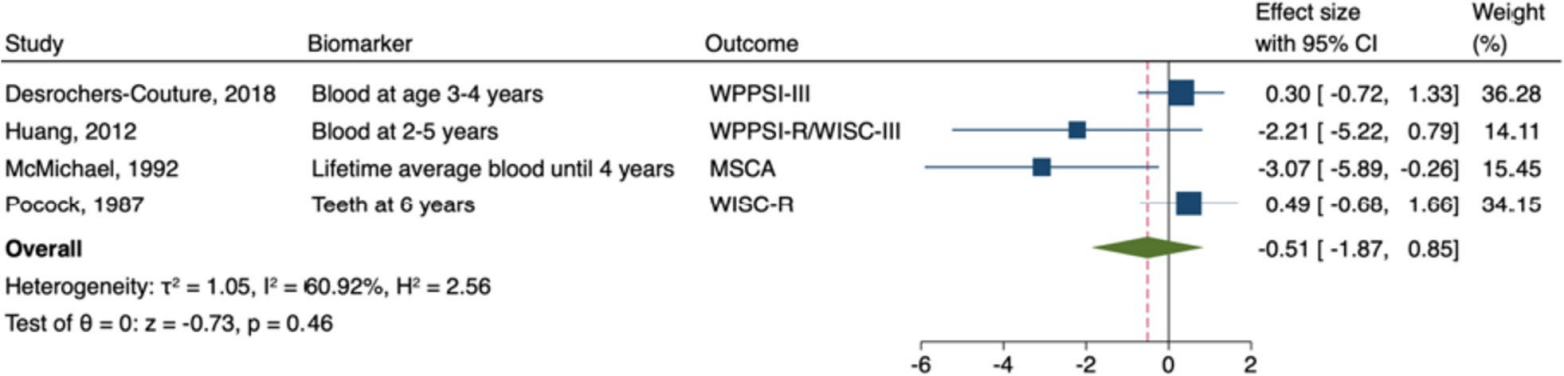
Random-Effect Meta-Analysis - Postnatal Lead and General Intelligence in Females

The meta-analysis of postnatal lead exposure and general intelligence included four studies [63, 83, 90, 101]. Postnatal exposure to lead was not significantly associated with general intelligence in males (B = -1.04; 95% CI: -2.21, 0.14; *I* ^2^ = 45%; Fig. 8) or in females (B = -0.51; 95% CI: -1.87, 0.85; *I* ^2^ = 61%; see Fig. 9).

#### Publication bias

Egger’s test did not indicate substantial funnel plot asymmetry for any of the random-effects meta-analyses, except for postnatal lead exposure in females (*z* = -2.66, *p* = 0.008; see Supplemental Figs. 1, 2, 3, 4, 5, 6, 7, 8).

#### Sensitivity analyses

In both males and females, the overall effect size of prenatal exposure and general, nonverbal, and verbal intelligence does not vary substantially regardless of the study excluded (see Supplemental Figs. 9, 10, 11, 12, 13, 14). However, for postnatal lead exposure in males, the overall effect size varies widely from -0.78 to -1.65, depending on the study excluded (see Supplemental Fig. 15). Similarly, in females, the overall effect size varies greatly from 0.15 to -1.34 (see Supplemental Fig. 16). The overall pooled effects of neurotoxicants on general and nonverbal intelligence in males and females did not change appreciably when only low risk of bias studies were included (see Supplemental Figs. 17, 18, 19, 20). However, the overall pooled effect for verbal intelligence became more negative (-0.02 to -0.57; but remained nonsignificant) in males and became more positive (0.19 to 0.28; but remained nonsignificant) in females (see Supplemental Figs. 21 and 22).

### Narrative results

#### Study characteristics

We tabulated the studies included in the systematic review but excluded from the meta-analysis (see Table 5). Studies were published from 1982 to 2021 and involved between 35 and 2128 participants from 23 different cohorts around the world. Eleven studies were conducted in North America, seven from Africa, six in Europe, five from Asia, and four in Australia. Ten studies pertained to lead, nine to mercury, three to PBDEs, two to OPPs, five to phthalates, and five to PCBs. Most of these studies measured neurotoxicant exposure in blood (*n* = 16), followed by hair (*n* = 7) and urine (*n* = 7), teeth (*n* = 2), and the placenta (*n* = 1). Seventeen studies measured toxicant exposure during the prenatal period, seven studies measured toxicant exposure during the postnatal period, and seven measured toxicant exposure during both the prenatal and postnatal period. Intelligence was assessed using a variety of measures.

**Table 5:** Systematic Review Study Characteristics

Lead Author, Year	Country (Cohort)	N (%_males_)	Neuro-toxicant	Compound	Biomarker	Median of Exposure (ppb)	Outcome Measure	Interpretation	Reason Excluded from Meta-Analysis
Baghurst, 1992 [56]	Australia (Port Pirie Cohort Study)	494 (N/A)	Lead	N/A	Lifetime average blood up to 3 years	122.0–282.0*^a^	FSIQ on WISC-R at 7 years	The negative association between lead exposure and FSIQ was more pronounced in females	No standard error or p-value to calculate effect size
Berghuis, 2018 [51]	Netherlands (DACE Study)	35–40 (~ 54.5)	PCB PBDE	ΣOH-PCBs^c^ BDE-47	2^nd^/3^rd^ trimester serum 2^nd^/3^rd^ trimester serum	0.4 0.9	FSIQ, PRI, and VCI on WISC-III-NL at 13–15 years	Positive association between ΣOH-PCBs and VCI and FSIQ in females. Negative association between BDE-47 and FSIQ in females	No other combinable study of OH-PCBs or BDE-47 (only data for females)
Castorina, 2017 [58]	USA (CHAMACOS)	248–275 (~ 54.8)	OPP OPP OPP	BDCIPP DPHP ip-PPP	2^nd^ trimester urine 2^nd^ trimester urine 2^nd^ trimester urine	0.4 0.9 0.3	FSIQ, PRI, VCI on WISC-IV at 7 years	No significant differences between the sexes	No data available
Chen, 2014 [102]	USA (HOME)	190 (44.0)	PBDE	BDE-47	2^nd^ trimester blood	18.9	FSIQ on WPPSI-III at 5 years	No significant differences between the sexes	Not enough combinable date to meta-analyze BDE-47
Choi, 2021 [100]	Norway (MoBA, Preschool ADHD Sub-Study)	310 (56.9)	Phthalate Phthalate Phthalate Phthalate Phthalate Phthalate	MBzP, MiBP, MnBP, MEP, ∑DEHP^d^ ∑DiNP^e^	2^nd^ trimester urine 2^nd^ trimester urine 2^nd^ trimester urine 2^nd^ trimester urine 2^nd^ trimester urine 2^nd^ trimester urine	5.45^b^ 19.64^b^ 21.70^b^ 129.71^b^ 0.30^b^ 0.02^b^	Verbal and non-verbal WM on SB5 at 3.5 years	The negative association between MiBP, MnBP, and MEP and non-verbal WM was more pronounced in females	Non-combinable outcome measure (i.e., verbal, and nonverbal WM)
Damm, 1993 [59]	Denmark (N/A)	141–162 (45.4–46.9)	Lead	N/A	Shed deciduous teeth at 6 years	Low: < 5,000 High: > 18,700	FSIQ, PIQ, VIQ on the Danish version of the WISC at 8 and 15 years	Females were better at coping with lead-related deficits in PIQ compared to males	No data available
Davidson, 2006 [62]	Republic of Seychelles (SCDS)	711 (N/A)	Mercury Mercury Mercury	N/A	Maternal hair at delivery Hair at 5.5 years Hair at 5.5 years	6800.0^a^ 6500.0^a^ 6100.0^a^	GCI on MSCA at 5.5 years and FSIQ on WISC-III at 8.9 years	Positive association between postnatal mercury exposure and global cognition in males	No data available for prenatal exposure. Not enough combinable data to meta-analyze postnatal exposure
Davidson, 2004 [61]	Republic of Seychelles (SCDS)	711 (N/A)	Mercury	N/A	Maternal hair at delivery	5900.0	GCI on MSCA at 5.5 years	No significant differences between the sexes	No data available
Davidson, 1998 [60]	Republic of Seychelles (SCDS)	711 (N/A)	Mercury Mercury	N/A	Maternal hair at delivery Hair at 5.5 years	6800.0^a^ 6500.0^a^	GCI on MSCA at 5.5 years	No significant differences between the sexes	No data available
Ernhart, 1989 [65]	USA (N/A)	146–169 (N/A)	Lead Lead	N/A	Cord blood Maternal blood at delivery	58.9^a^ 65.0^a^	FSIQ, PIQ, and VIQ on WPPSI at 4 years 10 months	No significant differences between the sexes	No data available
Factor-Litvak, 2014 [67]	USA (CCCEH)	328 (47.3)	Phthalate Phthalate Phthalate Phthalate Phthalate Phthalate	MnBP MBzP MEHHP MEHP MEP MiBP	3^rd^ trimester urine 3^rd^ trimester urine 3^rd^ trimester urine 3^rd^ trimester urine 3^rd^ trimester urine 3^rd^ trimester urine	38.0 14.4 21.8 4.9 141.5 9.2	FSIQ, PRI, and VCI on WISC-IV at 7 years	No significant differences between the sexes	No measure of overall DEHPs
Freire, 2018 [52]	Spain (INMA)	302 (71.5)	Lead Mercury	N/A	Placenta at birth Placenta at birth	< 6.5 0.025	GCI on MSCA at 4–5 years	The negative association between mercury and GCI is more pronounced in males	Same cohort, exposure, and outcome as Llop, 2017
Furlong, 2017 [68]	USA (The Mount Sinai Children’s Environmental Health Center)	141 (51.0)	OPP OPP	∑DEP^f^ ∑DMP^g^	2^nd^/3^rd^ trimester urine 2^nd^/3^rd^ trimester urine	2.5 4.7	FSIQ, VIQ and PIQ on the WPPSI-III at 6 years and FSIQ, PRI, VCI,on the WISC-IV between 7–9 years	No significant differences between the sexes	No data available
Golding, 2017 [70]	UK (ALSPAC)	1758–1759 (49.9)	Mercury	N/A	1^st^ trimester blood	1.86	FSIQ, PIQ, and VIQ on WISC-III at 8 years	No significant differences between the sexes	Non-linear regression techniques (logistic regression)
Huang, 2015 [72]	Taiwan (TMICS)	73–100 (N/A)	Phthalate Phthalate Phthalate Phthalate Phthalate	MMP MEP MBP MBzP ΣMEHP^h^	3^rd^ trimester urine, urine at 2–3, and urine at 5–6 years	49,840^b^ 66,610^b^ 77,870^b^ 17,430^b^ 58,690^b^	FSIQ on WPPSI-R at 5 years, WISC-III at 8 years and WISC-IV at 11 years	No significant differences between the sexes	No data available
Huang, 2007 [71]	Republic of Seychelles (SCDS)	643 (N/A)	Mercury	N/A	Maternal hair at delivery	5,990 – 8,790^a^	FSIQ on WISC-III at 9 years	No significant differences between the sexes	No data available
Ikeno, 2018 [74]	Japan (N/A)	141 (46.8)	PCBs PCBs PCBs	Non-Ortho Mono-Ortho Coplanar	2^nd^ trimester blood 2^nd^ trimester blood 2^nd^ trimester blood	0.059 8.029 8.101	MPS on the K-ABC at 3.5 years	Strong negative association for total non-ortho PCBs and MPS in males	No other combinable study of PCBs
Jacobson, 2002 [75]	USA (N/A)	124–158 (N/A)	PCBs PCBs PCBs	Total PCBs Total PCBs Total PCBs	Cord blood Maternal blood at birth Maternal milk at 0.5–4.5 months	2.6^a^ 5.7^a^ 829.7^a^	GCI, VS, and PPS on MSCA at 4 years and FSIQ, VCI, and PRI on WISC-R at 11 years	At 11 years, the negative effects of cord blood PCBs on FSIQ and PRI were more pronounced among males	No standard-error or exact p-value to calculate effect size
Kyriklaki, 2016 [78]	Greece (Rhea Study)	695 (51.6)	PCBs	Total PCBs	1^st^ trimester blood	0.32	GCI on MSCA at 4 years	No significant differences between the sexes	No data available
McBride, 1982 [81]	Australia (N/A)	84–88 (N/A)	Lead	N/A	Blood at 4–5 years	2629.4^b^	VIQ on the PPVT at 4–5 years	No interpretation made	Non-linear regression technique (mean scores)
McMichael, 1994 [82]	Australia (Port Pirie Cohort Study)	262 (43.3)	Lead	N/A	Incisor teeth at 6 years	8600^b^	FSIQ, PIQ, and VIQ on WISC-R at 7 years	No significant differences between the sexes	No data available
McMichael, 1988 [84]	Australia (Port Pirie Cohort Study)	474–534 (NA)	Lead Lead Lead Lead Lead Lead Lead	N/A	1^st^ trimester blood Cord blood Blood at 6 months Blood at 15 months Blood at 2 years Blood at 3 years Blood at 4 years	91.00^b^ 95.00^b^ 144.7^b^ 208.9^b^ 213.0^b^ 194.4^b^ 163.3^b^	GCI and PPS, on MSCA at 4 years	No significant differences between the sexes	No data available
Min, 2009 [85]	USA (N/A)	273–278 (~ 48)	Lead	N/A	Blood at 4 years	61.0	FSIQ, PIQ and VIQ, on WPPSI-R at 4 years and FSIQ, PRI, and VCI, on WISC-IV at 9 and 11 years	No significant differences between the sexes	No data available
Myers, 2003 [86]	Republic of Seychelles (SCDS)	643 (N/A)	Mercury	N/A	Maternal hair at delivery	6900.0^a^	FSIQ on WISC-III at 9 years	No significant differences between the sexes	No data available
Myers, 1995 [87]	Republic of Seychelles (SCDS)	217 (N/A)	Mercury	N/A	Maternal hair at delivery	7100.0	GCI and PPS on MSCA at 5.5 years	No significant differences between the sexes	No data available
Palumbo, 2000 [89]	Republic of Seychelles (SCDS)	708–711 (N/A)	Mercury Mercury	N/A	Maternal hair at delivery Hair at 5.5 years	6800.0^a^ 6500.0^a^	PPS and VS on MSCA at 5.5 years	No significant differences between the sexes	No data available
Rauh, 2011 [91]	USA (CCCEH)	265 (44.2)	OPP	CPF	Cord blood	0.003^a^	FSIQ, PRI, VCI, on WISC-IV at 7 years	No significant differences between the sexes	No data available
Ris, 2004 [92]	USA (Cincinnati Lead Study)	195 (53.6)	Lead	N/A	Maternal blood Blood samples up to 5 years	Range: 50.0–270.0	Learning/IQ factor derived from WRAT-3 and WISC-III at 15–17 years	No significant differences between the sexes	No data available
Schnaas, 2000 [93]	Mexico (Mexico City Prospective Lead Study)	112 (47.3)	Lead	N/A	Blood at 6–18 months Blood at 24–36 months Blood at 42–54 months	101^b^ 97^b^ 84^b^	GCI on MSCA at 3, 3.5, 4, 4.5 and 5 years	No significant differences between the sexes	No data available
Tatsuta, 2014 [94]	Japan (TSCD)	387 (52.2)	PCBs	9CBs	Cord blood	0.22	MPS on K-ABC at 3.5 years	The negative association between the MPS and 9CBs was more pronounced in males	No other combinable study of 9CBs
Vuong, 2017 [98]	USA (HOME)	208 (44.7)	PBDE	∑PBDEs^i^	Blood at 1 year Blood at 2 years Blood at 3 years Blood at 5 years	118.1^b^ 127.7^b^ 100.5^b^ 64.6^b^	FSIQ on WISC-IV at 8 years	No significant differences between the sexes	No other combinable study of postnatal PBDEs
Zhu, 2020 [99]	China (MABC)	1865–2128 (~ 52.0)	Phthalate Phthalate Phthalate Phthalate Phthalate Phthalate Phthalate	MMP MBP MBzP MEHHP MEHP MEOHP MEP	Mean of three urines Mean of three urines Mean of three urines Mean of three urines Mean of three urines Mean of three urines Mean of three urines	N/A N/A N/A N/A N/A N/A N/A	FSIQ, VCI, and VSI on WPPSI-IV (Chinese version) between 3 to 6 years	Negative association between MBP and VSI and FSIQ in males, whereas in girls’ results were attenuated near zero. Positive association between MEHP and FSIQ in males and MBzP and VCI in males/	No measure of overall DEHPs

^*^Mean low to mean high quartiles

^a^Arithmetic mean, ^b^geometric mean

^c^ΣOH-PCBs = 4-OH-PCB-107 + 4-OH-PCB-146 + 4-OH-PCB-187 + 3-OH-PCB-153 + 3′-OH-PCB-138 + 4′-OH-PCB-172; ^d^∑DEHP = MEHHP + MEHP + MEOHP + MECPP + MMCHP; ^e^∑DiNP = cx-MiNP + oxo-MiNP + OH-MiNP; ^f^∑DEP = DEP + DETP + DEDTP; ^g^∑DMP = DMP + DMTP + DMDTP; ^h^ ΣMEHP = MEHP + MEHHP + MEOHP; ^i^∑PBDE = BDE-28 + BDE-47 + BDE-99 + BDE-100 + BDE-153;

*Abbreviations* *:* *ADHD* Attention-Deficit/Hyperactivity Disorder, *ALSPAC* Avon Longitudinal Study of Parents and Children, *BDCIPP* Bis(1,3-dichloro-2-propyl) Phosphate*,* *BDE* Brominated Diphenyl Ethers, *BDE-47* Tetrabromodiphenyl Ether*,* *CCCEH* Columbia Center for Children's Environmental Health*,* *CHAMACOS* Center for the Health Assessment of Mothers and Children of Salinas*,* *CPF* Chlorpyrifos*,* *cx-MiNP* Mono-4-methyl-7-carboxyheptyl phthalate*,* *DACE* Development at Adolescence and Chemical Exposure*,* *DAP* Dialkyl Phosphates*,* *DEDTP* Diethyl Dithiophosphate*,* *DEHP* Bis(2-ethylhexyl) Phthalate*,* *DEP* Diethyl phosphate*,* *DETP* Diethyl Thiophosphate*,* *DiNP* Diisononyl Phthalate*,* *DMDTP* Dimethyl Dithiophosphate*,* *DMP* Dimethyl Phosphate*,* *DMTP* Dimethyl Thiophosphate*,* *DPHP* Di(2-propylheptyl) Phthalate*,* *FSIQ* Full-Scale IQ*,* *GCI* General Cognitive Index*,* *HOME* Health outcomes and measures of the environment*,* *INMA* INfancia y Medio Ambiente (Environment and Childhood) Project*,* *ip-PPP* 2-((isopropyl) phenyl) Phenyl Hydrogen Phosphate*,* *IQ* Intelligence Quotient*,* *K-ABC* Kaufman Assessment Battery for Children*,* *MBP* Monobutyl Phthalate*,* *MBzP* Monobenzyl Phthalate*,* *MECPP* Mono(2-ethyl-5-carboxypentyl) Phthalate*,* *MEHHP* Mono(2-ethyl-5-hydroxyhexyl) Phthalate*,* *MEHP* Mono(2-ethylhexyl) Phthalate*,* *MEOHP* Mono-(2-ethyl-5-oxohexyl) Phthalate*,* *MEP* Mono-ethyl Phthalate*,* *MiBP* Mono-isobutyl Phthalate*,* *MMP* Mono-methyl Phthalate*,* *MMCHP* Mono-2-methylcarboxyhexyl phthalate*,* *MnBP* Mono-n-butyl Phthalate*,* *MoBA* The Norwegian Mother & Child Cohort Study (den norske Mor & barn-undersøkelsen)*,* *MPS* Mental Processing Scale*,* *MSCA* McCarthy Scales of Children's Abilities*,* *OH-MiNP* Mono-4-methyl-7-hydroxyoctyl phthalate*,* *OH-PCB* Hydroxylated Polychlorinated Biphenyl, *OPP* Organophosphate Pesticides*,* *oxo-MiNP* Mono-4-methyl-7oxooctyl phthalate*,* *PBDE* Polybrominated Diphenyl Ethers*,* *PCB* Polychlorinated Biphenyls*,* *PIQ* Performance Intelligence*,* *PPS* Perceptual Performance Scale*,* *PPVT* Peabody Picture Vocabulary Test*,* *PRI* Perceptual Reasoning Index*,* *SB5* Standford Binet 5*,* *SCDS* Seychelles Child Development Study*,* *SON-R* Snijders-Oomen Nonverbal Intelligence Test*,* *TMICS* Taiwan Maternal and Infant Cohort Study*,* *TSCD* Tohoku Study of Child Development*,* *UK* United Kingdom*,* *USA* United States of America*,* *VCI* Verbal Comprehension Index*,* *VIQ* Verbal Intelligence*,* *VS* Verbal Scale*,* *VSI* Visual Spatial Index*,* *WISC-III* Wechsler Intelligence Scale for Children, Third Edition*,* *WISC-IV* Wechsler Intelligence Scale for Children, Fourth Edition*,* *WISC-III-NL* Wechsler Intelligence Scale for Children, Third Edition, Netherlands Version*,* *WISC-R* Wechsler Intelligence Scale for Children, Revised*,* *WPPSI-III* Wechsler Preschool & Primary Scale of Intelligence, Third Edition*,* *WPPSI-R* Wechsler Preschool & Primary Scale of Intelligence, Revised*,* *WRAT-III* Wide Range Achievement Test, Third Edition

The risk of bias ratings for the studies included in the systematic review but excluded from the meta-analysis are shown (see Table 6). Ten studies were rated as “low” or “probably low” risk of bias across all the areas, whereas 23 studies were rated a “high” or probably high” risk of bias in one or more area. Studies rated as “high” or “probably high” risk of bias were mostly rated as such in the areas of selection bias, confounding, and incomplete outcome data. Our rationale for each study’s rating across each area is provided in Supplementary Tables 1– 32.

**Table 6:**
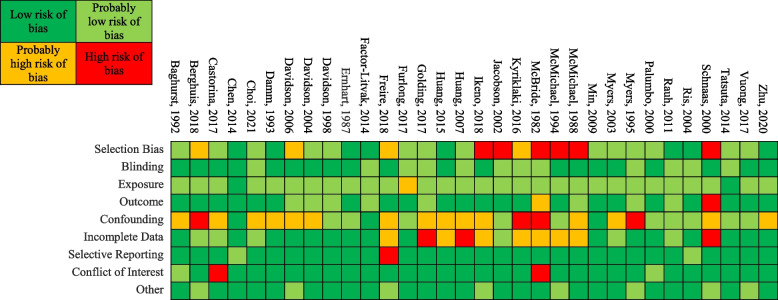
Risk of bias ratings for studies included in the systematic review

Seventeen studies examined the association between prenatal exposure to developmental neurotoxicants and general intelligence. Of those, six were rated as “low” or “probably” low risk of bias across all nine areas. Five of the six low risk of bias studies found no significant differences between the sexes—two examined lead [65, 92], one BDE-47 [102], one OPPs [91]**,** and one phthalates [67]. One low risk of bias study found a negative association between MBP and general intelligence in males, but also a positive association between MEHP and general intelligence in males [99].

Twelve studies examined the association between prenatal exposure to developmental neurotoxicants and nonverbal intelligence. Five of those were rated as “low” or “probably” low risk of bias across all nine areas. Three of five low risk of bias studies found no significant differences between the sexes – one examined phthalates [67], one lead [65], and one OPPs [91]. One low risk of bias study found a negative association between PCBs and the mental processing scale in males [94]; another found a negative association between MBP and nonverbal intelligence in males [99].

Eight studies examined the association between prenatal exposure to developmental neurotoxicants and verbal intelligence. Of those, four were rated as “low” or “probably” low risk of bias across all nine areas and three of which found no significant differences between the sexes – one lead [65], one phthalates [67], and one OPPs [91]. One low risk of bias study found a positive association between MBzP and verbal intelligence in males [99].

Nine studies examined the association between postnatal exposure to developmental neurotoxicants and general intelligence. Four of those were rated as “low” or “probably” low risk of bias across all nine areas, all of which found no significant differences between the sexes—two examined lead [85, 92], one mercury [60], and one PBDEs [98].

Four studies examined the association between postnatal exposure to developmental neurotoxicants and nonverbal intelligence. Of those, two were rated as “low” or “probably” low risk of bias across all nine areas, both of which found no significant differences between the sexes—one examined lead [85] and one mercury [89].

Lastly, three studies examined the association between postnatal exposure to developmental neurotoxicants and verbal intelligence. Of those, two were rated as “low” or “probably” low risk of bias across all nine areas, both of which found no significant differences between the sexes—- one examined lead [85] and one mercury [89].

#### Certainty of the evidence

The certainty of the evidence is summarized in Table 7. All studies included in this meta-analysis were cohort studies, which led to an initial rating of "moderate confidence." Publication bias was identified as the primary factor downgrading the quality of evidence for all outcomes because our results are limited by the exclusion of studies that did not report sex-specific effects. Imprecision was deemed unlikely due to the tightness of the upper-to-lower 95% confidence interval ratios across outcomes. Directness was not downgraded, as all studies included human participants with validated biomarkers of exposure and standardized IQ measures. Moreover, there was neither a dose–response gradient nor large effects; thus, no upgrade in the quality of evidence was warranted. Supplementary Tables 1, 2, 3, 4, 5, 6, 7, 8 provide further rationale for each rating.

**Table 7:** Ratings for the certainty of the evidence

	**Boys**				**Girls**			
**Outcomes**	**Prenatal General**	**Prenatal Nonverbal**	**Prenatal Verbal**	**Postnatal Lead**	**Prenatal General**	**Prenatal Nonverbal**	**Prenatal Verbal**	**Postnatal Lead**
**Initial Rate of Confidence**	Moderate	Moderate	Moderate	Moderate	Moderate	Moderate	Moderate	Moderate
**Downgrading Factors**								
Risk of Bias	0	0	-1	-2	0	0	0	-2
Inconsistency	0	0	0	-1	-1	0	0	-1
Indirectness	0	0	0	0	0	0	0	0
Imprecision	0	0	0	0	0	0	0	0
Publication Bias	-1	-1	-2	-1	-1	-1	-2	-2
**Upgrading Factors**								
Large Magnitude	0	0	0	0	0	0	0	0
Dose Response	0	0	0	0	0	0	0	0
Residual Confounding	0	0	0	0	0	0	0	0
**Overall**								
Quality	Moderate	Moderate	Low	Low	Low	Moderate	Moderate	Low
Strength	Limited Evidence of Toxicity	Limited Evidence of Toxicity	Inadequate Evidence of Toxicity	Inadequate Evidence of Toxicity	Inadequate Evidence of Toxicity	Insufficient Evidence of toxicity	Inadequate Evidence of Toxicity	Inadequate Evidence of Toxicity

Overall, the quality of the evidence remained rated as “moderate” for prenatal exposure effects on general and nonverbal intelligence in males and general and verbal intelligence in females. In contrast, we downgraded the quality of the evidence to “low” for prenatal exposure to verbal intelligence in boys and general intelligence in girls. Similarly, we downgraded the quality of the evidence to “low” for postnatal lead exposure in both boys and girls.

On the basis of our “moderate” level ratings for the confidence and quality of evidence that was synthesized, we rated the overall strength of the evidence as providing “limited” evidence of toxicity for prenatal exposure on general and nonverbal intelligence in boys. In contrast, we rated the strength of the evidence as providing “insufficient” evidence for toxicity for prenatal exposure on nonverbal intelligence in females. For all other outcomes, we rated the strength as “inadequate” evidence of toxicity, which could reflect either a “low” overall quality of evidence or a “moderate” overall quality with “limited” evidence of toxicity.

## Discussion

We conducted a systematic review and series of meta-analyses to examine effects of pre-and post-natal exposure to six neurotoxicants on males’ and females’ intelligence. Although there have been several systematic reviews examining the sex-specific neurodevelopmental impacts from heavy metals [6, 8, 34] and developmental neurotoxicants [7], none have quantitatively examined the strength of the evidence while considering timing of exposure and IQ as the specific outcome measure. Combining data from individual studies using meta-analytic techniques produces a more precise and objective estimate of the underlying effects of neurotoxicants on males’ and females’ intelligence compared to systematic review technqiues.

We found that prenatal exposure to developmental neurotoxicants (lead, mercury, phthalates, PBDEs, and OPPs) was associated with a decrement in general and nonverbal intelligence, but only among males. Specifically, the pooled effect demonstrated that a 50% difference in exposure level was associated with a 0.38 and 0.42 decrease in general and nonverbal intelligence, respectively, among males – the quality of the evidence was considered “moderate”. Despite these moderate ratings, we consider the overall strength rating to reflect “limited” evidence of toxicity (as opposed to “sufficient” evidence) given that the pooled effect size is relatively small in magnitude and the conclusion could be affected by results of future studies based on potential for bias of the existing literature. Further, the observed sex-specific effect on general and nonverbal intelligence in males was largest for prenatal lead exposure.

### Prenatal exposure

#### Theories and mechanisms underlying a male vulnerability to prenatal exposures

Various studies have documented the sex-specific effects of neurotoxicant exposure on a variety of cellular, hormonal, and molecular endpoints, providing potential mechanisms to explain the male vulnerability of prenatal exposure to intellectual abilities. The sex-specific impact of prenatal exposure to developmental neurotoxicants on intelligence is consistent with a growing body of research demonstrating a protective placental female effect in response to maternal perturbations [103]. Specifically, the female placenta conserves resources and adjusts to the maternal milieu through a greater expression of genes and proteins associated with transport, immune regulation, growth, and development than the male placenta [104, 105]. The male placenta, instead, invests resources in growth [106]. As a result, the male fetus has a limited ability to adjust to adversity and is at greater risk for subsequent morbidity and mortality [103]. This pattern is consistently seen in response to exposure to prenatal stress [107].

A similar pattern can be seen in relation to exposure to developmental neurotoxicants. While the placenta plays an essential role in the regulation of the fetal environment, and in the communication and transportation of nutrients to the developing fetus, it is also implicated in fetal exposure to neurotoxicants [108]. The greater expression of genes in female fetuses may then be better equipped to respond to and buffer against neurotoxicants. For example, neurotoxicants (e.g., lead, phthalates) are known to impact intrauterine inflammation and oxidative stress [109, 110], and several studies demonstrate that females are resilient to maternal immune perturbations or oxidative stress, while males are at increased risk [111]. Specifically, intrauterine inflammation has been found to induce a neuroinflammatory response (e.g., up-regulation of pro-inflammatory cytokines in the brain and greater macrophage activation) only in males [112–114]. One study examined twins exposed to the same environmental insult during fetal development and found that male infants at birth had higher levels of oxidative stress in the placenta than females, suggesting that the male fetus may be more susceptible to maternal oxidative stress than the female fetus [115]. Neuroinflammation and oxidative stress in offspring can alter fetal developmental trajectories, which can have detrimental downstream effects on the development of cognitive abilities and ultimately the pathogenesis of intellectual disability [113, 116–118].

There is also evidence that male and female fetuses differ in the rate of neural maturation in utero, where male fetuses’ nervous system functioning matures at a slower rate than females. For example, using fetal heart rate responses to vibroacoustic stimulation (VAS) as a proxy for fetal nervous system development and functioning, studies have found that male fetuses recover more slowly to VAS at 31 weeks’ gestation compared to female fetuses [119]. This difference in maturation may reflect a greater sensitive time window in which males’ brains are more vulnerable to the impact of neurotoxicants.

Furthermore, estradiol and progesterone, the two main circulating female sex hormones, have been found to have neuro-protective and neuro-reparative properties during brain development [120–122]. For example, they can enhance cell proliferation, synaptic plasticity, axonal growth, and remyelination, as well as decrease oxidative stress and neuroinflammation [120, 122]. Males typically have fewer estrogen receptors throughout their central nervous system than females [24]. Thus, differing levels of sex hormones offer another reason that females may be more protected from adverse neurological effects from prenatal exposure to neurotoxicants.

Sex differences in epigenetics have been proposed as another mechanism to explain prenatal sex differential vulnerability [123]. DNA methylation, a type of epigenetic mechanism, may impact neurodevelopment [124], and some studies have found that neurotoxicants can impact DNA methylation differently in males and females [123]. In one study, perinatal exposure to lead resulted in the hypomethylation of a gene in the hippocampal methylome associated with learning and memory, but only in males [125]. In humans, prenatal mercury levels were associated with cord blood DNA methylation changes at the Paraoxonase 1 (PON1) gene in males but not females [126]. Further, cord blood DNA methylation changes at the PON1 gene ultimately predicted lower cognitive test scores during early childhood [126]. Lastly, in humans, maternal exposure to persistent organic pollutants, such as PBDEs, has been found to be associated with increased methylation of the monocarboxylate transporter 8 (MCT8) gene in the placenta, but only in male infants [127]. Importantly, the MCT8 is X-linked and thus, males are more vulnerable than females to impairments in this gene [127]. Higher methylation of this gene can interfere with the transport of T_4_ to the fetus, decreasing the amount of circulating thyroid hormone levels critical for neurodevelopment [127].

#### Domain-specific effects

We found that prenatal exposure to developmental neurotoxicants was negatively associated with males’ general and nonverbal intelligence rather than verbal intelligence. Nonverbal intelligence encompasses an individual’s ability to perceive, process, and manipulate information using visual and spatial reasoning. This domain- and sex-specific effect may be explained by the vulnerability hypothesis based on evolutionary and sexual selection theory [128]. According to this theory, sexually selected cognitive advantages are supported by elaborated brain networks (i.e., more neural tissue and more intermodular connections) that are highly energy dependent [128]. Thus, more elaborated traits are more vulnerable to disruptions in energy production and oxidative stress, as they require more energy to build, maintain, and express [128]. Furthermore, cognitive vulnerabilities are likely to be greatest during trait development and under conditions that require maximum trait expression [128].

The male advantage in visuospatial abilities is a sexually selected trait, as it supports aspects of male-male competition [129]. In line with the vulnerability hypothesis, males have larger volumes and higher tissue density in the hippocampi than females [130], an area involved in higher-order visual-spatial perception [131]. The visual system develops early in the prenatal period. By the third trimester of pregnancy, the human fetus has the capacity to process perceptual information [132]. Thus, visual-spatial abilities in males could be susceptible to energy disruptions during prenatal development. Neurotoxicants can induce oxidative stress in males and that males may be more vulnerable to the effects of it [115, 133]. Thus, the combination of sex and developmental neurotoxicity in fetal life may act synergistically as a double-hit model to disrupt energy production and ultimately affect visual-spatial abilities in males.

### Findings by Neurotoxicant

#### Lead

Although the effect sizes did not significantly vary by neurotoxicant in males, in subgroup analyses, the strongest and most consistent effects on general and nonverbal intelligence in males were for prenatal lead exposure. Specifically, the pooled effect demonstrated that every 50% difference in prenatal lead levels was associated with a 1.07-point and 1.18-point decrease in general and nonverbal intelligence, respectively, in males, whereas the pooled effect among females was a 0.45-point and 0.15-point increase (albeit non-significant) for general and nonverbal intelligence, respectively. This result is not surprising given that the global number of disabilities-adjusted life years of idiopathic developmental intellectual disability in 2019 was 4.39 million, of which 61.90% was attributable to lead exposure [134]. Further, despite global efforts to reduce lead exposure, and research demonstrating that no level of lead is safe, around 1 in 3 children – up to approximately 800 million globally – have blood lead levels at or above 5 µg per deciliter (μg/dL) [135].

Moreover, relative to other neurotoxicants, lead may induce greater negative effects in males due to the interaction between lead and stress, as mothers with exposure to chronic stress (e.g., those with low socioeconomic status or resource deprivation) are more likely to be exposed to higher levels of lead, indicating the potential for a multiple stressor model [136–138]. Prenatal lead exposure and stress both disrupt the programming of the hypothalamic–pituitary–adrenal axis [138]. Further, combined exposure to prenatal lead and stress has been found to alter epigenetic profiles in the brain in sex-specific manner [139–141].

#### Mercury

The subgroup effect of mercury on general intelligence in males had a moderate to high degree of heterogeneity across studies; some studies showed positive effects and others negative effects. This variability is expected and may be due to differences in exposure levels, the confound of fish intake, or the underlying genetic composition of the populations. For example, the mean or median of exposure ranged from 0.4 to 26.0 ppb across the studies [53, 54, 76, 80]. Lower levels of exposure may be indicative of lower consumption of fish. Fish contains essential fatty acids and nutrients such as n-3 polyunsaturated fatty acids, iodine, selenium, vitamin D and B12 that are crucial for the development of the fetal brain [142]; thus lower consumption could also adversely impact neurodevelopment. In fact, Llop and colleagues (2017) found that mercury concentration was only associated with lower scores among children whose mothers consumed fewer than three weekly servings of fish during pregnancy [80]. Moreover, in Julvez et al. (2013), the presence of a specific genetic polymorphism was associated with greater mercury neurotoxicity [76]. The differences across and within studies demonstrate the complexity of isolating effects of exposure on IQ by sex without considering other environmental and genetic factors.

#### Phthalates

We found that the sum of DEHPs was significantly positively associated with nonverbal intelligence in females. Despite this, the pooled effect of developmental neurotoxicants on nonverbal intelligence in females was non-significant and effects did not significantly differ by neurotoxicant. However, generally, effect sizes for the sum of DEHPs were near zero or positive in females. The possible protective or positive effect in females is intriguing. Phthalates are endocrine disrupters and one study found that exposure to phthalates later in pregnancy was associated with increased estrogens in women carrying female fetuses [143]. In contrast, exposure to phthalates earlier in pregnancy was associated with decreased estrogen (albeit not significantly) in women carrying male fetuses [143]. While the increased estrogen may confer a benefit to females with respect to the neurotoxic effects of phthalates due to its neuro-protective properties [120], it may also put females at increased risk for other adverse health outcomes, such as endometriosis, earlier onset of puberty, polycystic ovarian syndrome, breast cancer, or metabolic disorders [144]. Given the relative inconsistency in results of phthalates in females across outcomes (i.e., null for general and verbal intelligence, versus positive for nonverbal intelligence), more research is needed.

#### OPPs and BDEs

We found that the effect sizes for OPPs and BDEs in males were generally negative for general intelligence, consistent with the findings overall. Similarly, we found that the effect sizes for OPPs and BDEs in females were generally negative for general intelligence; however, there were moderate to high degrees of heterogeneity across studies. For nonverbal intelligence, we found that the effects sizes were generally closer to zero. Still, only two studies were available for each of these neurotoxicants and we found a high degree of heterogeneity for females, suggesting that more research is needed.

### Postnatal exposure

We did not find a significant pooled effect of postnatal lead exposure on either males or females general intelligence; however, the effect size in males was negative, as was seen with prenatal exposures. Nevertheless, we were limited by the small number of estimates, most of the studies included were considered higher risk of bias, there was an indication of publication bias, and there were discrepancies in the pooled effect sizes when individual studies were excluded. Further, we were unable to assess the effect of postnatal exposure to other chemicals or on other domains of intelligence which could have been more sensitive to sex-specific effects. Since infancy is a critical period for the development of language [145] and females have advantages in language competence, females may be more vulnerable to energy disruptions to verbal abilities during the early postnatal period, consistent with the vulnerability hypothesis [128]. More research on the sex-specific effects of postnatal neurotoxicant exposure is needed, with consideration of domain-specific effects of intelligence.

### Qualitative findings

Most studies that were not included in the meta-analysis concluded that no differences were noted between the sexes. Many of these studies did not report separate estimates for males and females, and therefore we were unable to determine the general trends of the individual effects. Further, studies of smaller sample sizes may have had inadequate power to detect sex differences through an interaction effect, which highlights the need for studies to report individual effect estimates for males and females to allow for inclusion in a meta-analysis.

Moreover***,*** few studies published to date have evaluated the sex-specific impact of PCB exposure on intellectual abilities. Of the studies that did evaluate the sex-specific impact of PCB exposure on intellectual abilities, the methods were too heterogenous (i.e., different congeners of PCBs or outcomes), thus limiting our ability to include PCB-related effects in the meta-analysis. However, consistent with our meta-analytic findings, four out of the five studies of prenatal PCB exposure found stronger negative associations in males or more positive associations in females [51, 75, 78, 94].

### Limitations and future directions

This study had some limitations. First, only 22 studies were included in this meta-analysis. Given the limited number of estimates available, we included studies from the same cohort with the same methodology if they had data on different neurotoxicants; doing so artificially reduced the standard error of our estimates. Further, there were very few studies that evaluated the sex specific effects of individual phthalate, PCB, PBDE, or OPP compounds and there are additional challenges with grouping individual compounds given differences based on relative toxicities and half-lives. Future systematic reviews on this topic would require a sizeable literature base to disentangle the sex-specific effects of specific individual compounds.

Second, the impact of environmental chemicals on intellectual abilities is a global issue [146]. However, Project TENDR emphasizes developmental neurotoxicants that are particularly relevant in the United States context. It is important to acknowledge that these are not the only chemicals that warrant scrutiny and investigation; for example, chemicals like fluoride, which may be regarded as significantly neurotoxic in diverse global contexts [147], cannot be overlooked. Further, most of the studies on the sex-specific effects of pre- and post-natal exposure to developmental neurotoxicants were conducted in post-industrial countries. The effect of lead on IQ has been found to be stronger in developing countries [148]; however, sex differences on this effect have not been investigated in developing countries. Future research evaluating the impact of neurotoxicants on IQ in developing countries should examine sex-specific effects.

Third, this review focused on pre- and early post-natal exposure. Exposure during early adolescence may represent another critical window where sex-specific effects occur. Adolescence is characterized by substantial structural and functional brain changes, particularly in regions associated with higher-level cognitive processing [149]. Further, there is evidence for sexually dimorphic trajectories of these brain changes [150]. Different behaviours and body weight between genders may also result in differences in exposure levels [27]. Future research should explore early adolescence as a critical window of exposure and take special consideration of sex- and potentially gender-specific effects during this time.

Fourth, while intellectual abilities are the most common neurodevelopmental outcome studied, neurodevelopment can also encompass other outcomes such as attention, motor skills, social skills, reading ability, and memory. Given the higher prevalence of ADHD and ASD in males, future research should explore the potential sex-specific effects related to neurotoxicant exposure on outcomes related to these disorders. Further, although females may demonstrate adaptive flexibility in response to gestational exposures and development of their general intellectual and nonverbal abilities, this adaptability does not preclude the possibility that developmental neurotoxicants influence the development of females. Research should explore the sex-specific effects related to neurotoxicant exposure on other outcomes such as social and psychological outcomes, given the greater prevalence of internalizing disorders in females [151].

Lastly, this review focused on the effects of exposure to a single developmental neurotoxicant on children’s neurodevelopment. Yet, developmental toxicants often co-occur – particularly among at-risk populations [152]- which can produce additive or synergistic effects that may initiate a developmental cascade, as shown with the example of lead and prenatal stress [152]. Thus, the effect in males is likely an underestimate of the true nature of the problem. Additional studies are needed to examine the sex-specific effects of cumulative exposure to environmental stressors, especially in human epidemiological cohorts.

## Conclusions

This is the first study to quantitatively synthesize the sex-specific effects of pre- and post-natal exposure to developmental neurotoxicants on intelligence. Overall, this meta-analysis demonstrated that males’ general and nonverbal intelligence are more impacted by prenatal exposure to developmental neurotoxicants than females, especially from lead exposure. This study highlights the necessity to include sex as a fundamental variable when examining the effects of developmental neurotoxicants on intellectual abilities. Even mild IQ deficits can have major academic, occupational, and psychological consequences [38]. In addition to the individual impacts, the economic impact is enormous. In fact, from 2001 to 2016, exposure to lead, mercury, PBDEs, and OPPs cost over $6 trillion in the US alone due to IQ point loss [153]. The results of this meta-analysis provide much needed insight into one of the influential factors in the sex bias of intellectual disabilities and highlight the possibility for early identification and prevention.

### Supplementary Information

**Additional file 1: Appendices Appendix A.** Risk of Bias Instructions. **Appendix B.** Effect Size Transformation.

**Additional file 2: Certainty of Evidence Table 1. **Prenatal Exposure and General IQ in Males. **Table 2. **Prenatal Exposure and Nonverbal IQ in Males. **Table 2. **Prenatal Exposure and Nonverbal IQ in Males. **Table 4. **Postnatal Lead Exposure and General IQ in Males. **Table 5. **Prenatal Exposure and General IQ in Females. **Table 6. **Prenatal Exposure and Nonverbal IQ in Females. **Table 7. **Prenatal Exposure and Verbal IQ in Females. **Table 8. **Postnatal Lead Exposure and General IQ in Females.

**Additional file 3. **Rationale for Risk of Bias Determinations for Each Study Included in the Meta-Analysis.

**Additional file 4. PRISMA Reporting Guidelines.**

**Additional file 5: Sensitivity Analyses Figure 1. **Funnel Plot - General Intelligence in Males. **Figure 2.** Funnel Plot - General Intelligence in Females. **Figure 3.** Funnel Plot - Nonverbal Intelligence in Males. **Figure 4.** Funnel Plot - Nonverbal Intelligence in Females. **Figure 5.** Funnel Plot - Verbal Intelligence in Males. **Figure 6.** Funnel Plot - Verbal Intelligence in Females. **Figure 7.** Funnel Plot - Postnatal Lead and General Intelligence in Males. **Figure 8.** Funnel Plot - Postnatal Lead and General Intelligence in Females. **Figure 9.** Leave One Out - General Intelligence in Males. **Figure 10.** Leave One Out - General Intelligence in Females. **Figure 11.** Leave One Out - Nonverbal Intelligence in Males. **Figure 12.** Leave One Out - Nonverbal Intelligence in Females. **Figure 13.** Leave One Out - Verbal Intelligence in Males. **Figure 14.** Leave One Out - Verbal Intelligence in Females. **Figure 15.** Leave One Out - Postnatal Lead and General Intelligence in Males. **Figure 16.** Leave One Out - Postnatal Lead and General Intelligence in Females. **Figure 17.** Low Risk of Bias - General Intelligence in Males. **Figure 18.** Low Risk of Bias - General Intelligence in Females. **Figure 19.** Low Risk of Bias - Nonverbal Intelligence in Males. **Figure 20.** Low Risk of Bias - Nonverbal Intelligence in Females. **Figure 21.** Low Risk of Bias - Verbal Intelligence in Males. **Figure 22.** Low Risk of Bias - Verbal Intelligence in Females. 

**Additional file 6. **Rationale for Risk of Bias Determinations for Each Study Included in the Narrative Synthesis.

## Data Availability

All data generated or analyzed during this study are included in this published article and its additional files.

## Abbreviations

9CB: 9 Chlorinated Biphenyls
ADHD: Attention-Deficit/Hyperactivity Disorder
ALSPAC: Avon Longitudinal Study of Parents and Children
ASD: Autism Spectrum Disorder
BDCIPP: Bis(1,3-dichloro-2-propyl) Phosphate
BDE: Brominated Diphenyl Ethers
BDE-47: Tetrabromodiphenyl Ether
CCCEH: Columbia Center for Children's Environmental Health
CHAMACOS: Center for the Health Assessment of Mothers and Children of Salinas
CI: Confidence Interval
CPF: Chlorpyrifos
C-WISC: Chinese Version of the Wechsler Intelligence Scale for Children
cx-MiNP: Mono-4-methyl-7-carboxyheptyl phthalate
DACE: Development at Adolescence and Chemical Exposure
DAP: Dialkyl Phosphates
DEDTP: Diethyl Dithiophosphate
DEHP: Bis(2-ethylhexyl) Phthalate
DEP: Diethyl phosphate
DETP: Diethyl Thiophosphate
DiNP: Diisononyl Phthalate
DMDTP: Dimethyl Dithiophosphate
DMP: Dimethyl Phosphate
DMTP: Dimethyl Thiophosphate
DNA: Deoxyribonucleic Acid
DPHP: Di(2-propylheptyl) Phthalate
ELEMENT: Early Life Exposures in Mexico to ENvironmental Toxicants
FSIQ: Full-Scale IQ
GCI: General Cognitive Index
HOME: Health Outcomes and Measures of the Environment
ID: Intellectual Disability
INMA: INfancia y Medio Ambiente (Environment and Childhood) Project
IOM: Institutes of Medicine
ip-PPP: 2-((Isopropyl) phenyl) Phenyl Hydrogen Phosphate
IQ: Intelligence Quotient
K-ABC: Kaufman Assessment Battery for Children
MABC: The Ma'anshan Birth Cohort
MBP: Monobutyl Phthalate
MBzP: Monobenzyl Phthalate
MECPP: Mono(2-ethyl-5-carboxypentyl) Phthalate
MEHHP: Mono(2-ethyl-5-hydroxyhexyl) Phthalate
MEHP: Mono(2-ethylhexyl) Phthalate
MEOHP: Mono-(2-ethyl-5-oxohexyl) Phthalate
MEP: Mono-ethyl Phthalate
MiBP: Mono-isobutyl Phthalate
MIREC: Maternal-Infant Research on Environmental Chemicals
MMP: Mono-methyl Phthalate
MMCHP: Mono-2-methylcarboxyhexyl phthalate
MnBP: Mono-n-butyl Phthalate
MoBA: The Norwegian Mother & Child Cohort Study (den norske Mor & barn-undersøkelsen)
MPS: Mental Processing Scale
MSCA: McCarthy Scales of Children's Abilities
NDD: Neurodevelopmental Disorder
OH-MiNP: Mono-4-methyl-7-hydroxyoctyl phthalate
OH-PCB: Hydroxylated Polychlorinated Biphenyls
OPP: Organophosphate Pesticides
oxo-MiNP: Mono-4-methyl-7oxooctyl phthalate
PBDE: Polybrominated Diphenyl Ethers
PCB: Polychlorinated Biphenyls
PIQ: Performance Intelligence
PON1: Paraoxonase 1
POP: Persistent Organic Pollutants
PPS: Perceptual Performance Scale
PPVT: Peabody Picture Vocabulary Test
PRI: Perceptual Reasoning Index
PRISMA: Preferred Reporting Items for Systematic Reviews and Meta-Analyses
SB5: Standford Binet 5
SCDS: Seychelles Child Development Study
SGOMSEC: Scientific Group on Methodologies for the Safety Evaluation of Chemicals
SMBCS: Sheyang Mini Birth Cohort Study
SON-R: Snijders-Oomen Nonverbal Intelligence Test
TENDR: Targeting Environmental Neuro-Development Risks
TMICS: Taiwan Maternal and Infant Cohort Study
TSCD: Tohoku Study of Child Development
UK: United Kingdom
USA: United States of America
VAS: Vibroacoustic Stimulation
VCI: Verbal Comprehension Index
VIQ: Verbal Intelligence
VS: Verbal Scale
VSI: Visual Spatial Index
WISC-III: Wechsler Intelligence Scale for Children, Third Edition
WISC-IV: Wechsler Intelligence Scale for Children, Fourth Edition
WISC-III-NL: Wechsler Intelligence Scale for Children, Third Edition, Netherlands Version
WISC-R: Wechsler Intelligence Scale for Children, Revised
WM: Working Memory
WPPSI-III: Wechsler Preschool & Primary Scale of Intelligence, Third Edition
WPPSI-R: Wechsler Preschool & Primary Scale of Intelligence, Revised
WRAT-III: Wide Range Achievement Test, Third Edition

## References

[1] Zablotsky B, Black LI, Maenner MJ, Schieve LA, Danielson ML, Bitsko RH, et al. Prevalence and trends of developmental disabilities among children in the United States: 2009–2017. Pediatrics. 2019;144 (4):e20190811. 10.1542/peds.2019-0811 PMC7076808 31558576

[2] May T, Adesina I, McGillivray J, Rinehart NJ (2019). Sex differences in neurodevelopmental disorders. Curr Opin Neurol..

[3] Lanphear BP. The impact of toxins on the developing brain. Annu Rev Public Health. 2015;36:211–30. 10.1146/annurev-publhealth-031912-114413 25581143

[4] Rauh VA, Margolis AE. Research Review: Environmental exposures, neurodevelopment, and child mental health – new paradigms for the study of brain and behavioral effects. J Child Psychol Psychiatry. 2016;57(7):775–93. 10.1111/jcpp.12537 PMC4914412 26987761

[5] Grandjean P, Landrigan PJ. Neurobehavioural effects of developmental toxicity. Lancet Neurol. 2014;13(3):330–8. 10.1016/S1474-4422(13)70278-3 PMC4418502 24556010

[6] Singh G, Singh V, Sobolewski M, Cory-Slechta DA, Schneider JS (2018). Sex-dependent effects of developmental lead exposure on the brain. Front Genet.

[7] Kern JK, Geier DA, Homme KG, King PG, Bjørklund G, Chirumbolo S (2017). Developmental neurotoxicants and the vulnerable male brain: A systematic review of suspected neurotoxicants that disproportionally affect males. Acta Neurobiol Exp (Warsz).

[8] Gade M, Comfort N, Re DB (2021). Sex-specific neurotoxic effects of heavy metal pollutants: Epidemiological, experimental evidence and candidate mechanisms. Environ Res.

[9] Grandjean P, Landrigan PJ (2006). Developmental neurotoxicity of industrial chemicals. Lancet Lond Engl.

[10] Rodier PM (1995). Developing brain as a target of toxicity. Environ Health Perspect.

[11] Woodruff TJ, Zota AR, Schwartz JM. Environmental chemicals in pregnant women in the united states: NHANES 2003- 2004. Environ Health Perspect. 2011;119(6):878–85. 10.1289/ehp.1002727 PMC3114826 21233055

[12] Arbuckle TE, Liang CL, Morisset AS, Fisher M, Weiler H, Cirtiu CM, et al. Maternal and fetal exposure to cadmium, lead, manganese and mercury: The MIREC study. Chemosphere. 2016;163:270–82. 10.1016/j.chemosphere.2016.08.023 27540762

[13] Sokoloff K, Fraser W, Arbuckle TE, Fisher M, Gaudreau E, LeBlanc A, et al. Determinants of urinary concentrations of dialkyl phosphates among pregnant women in Canada - Results from the MIREC study. Environ Int. 2016;94:133–40. 10.1016/j.envint.2016.05.015 27243443

[14] Anderko L. Project TENDR. Am J Nurs. 2017;117(5):61–64. 10.1097/01.NAJ.0000516275.74228.b6 28448366

[15] Schwartz J. Low-Level Lead Exposure and Children′s IQ: A Metaanalysis and Search for a Threshold. Environ Res. 1994;65(1):42–55. 10.1006/enrs.1994.1020 8162884

[16] Axelrad DA, Bellinger DC, Ryan LM, Woodruff TJ. Dose-response relationship of prenatal mercury exposure and IQ: An integrative analysis of epidemiologic data. Environ Health Perspect. 2007;115(4):609–15. 10.1289/ehp.9303 PMC1852694 17450232

[17] Schoeman K, Bend JR, Hill J, Nash K, Koren G. Defining a lowest observable adverse effect hair concentrations of mercury for neurodevelopmental effects of prenatal methylmercury exposure through maternal fish consumption: A systematic review. Ther Drug Monit. 2009;31(6):670–82. 10.1097/FTD.0b013e3181bb0ea1 19865003

[18] Pessah IN, Lein PJ, Seegal RF, Sagiv SK. Neurotoxicity of polychlorinated biphenyls and related organohalogens. Acta Neuropathol (Berl). 2019;138(3):363–87. 10.1007/s00401-019-01978-1 PMC6708608 30976975

[19] Sapbamrer R, Hongsibsong S (2019). Effects of prenatal and postnatal exposure to organophosphate pesticides on child neurodevelopment in different age groups: a systematic review. Environ Sci Pollut Res..

[20] Lam J, Lanphear BP, Bellinger D, Axelrad DA, McPartland J, Sutton P, et al. Developmental pbde exposure and IQ/ADHD in childhood: A systematic review and meta-analysis. Environ Health Perspect. 2017;125(8):086001. 10.1289/EHP1632 PMC5783655 28799918

[21] Ejaredar M, Nyanza EC, Ten Eycke K, Dewey D. Phthalate exposure and childrens neurodevelopment: A systematic review. Environ Res. 2015;142:51–60. 10.1016/j.envres.2015.06.014 26101203

[22] Arnold AP (2017). A general theory of sexual differentiation. J Neurosci Res.

[23] Gochfeld M. Sex Differences in Human and Animal Toxicology: Toxicokinetics. Toxicol Pathol. 2017;45(1):172–89. 10.1177/0192623316677327 PMC5371029 27895264

[24] Vahter M, Gochfeld M, Casati B, Thiruchelvam M, Falk-Filippson A, Kavlock R (2007). Implications of gender differences for human health risk assessment and toxicology. Environ Res.

[25] Wells JCK (2007). Sexual dimorphism of body composition. Best Pract Res Clin Endocrinol Metab.

[26] Kirchengast S (2010). Gender Differences in Body Composition from Childhood to Old Age: An Evolutionary Point of View. J Life Sci.

[27] Arbuckle TE (2006). Are there sex and gender differences in acute exposure to chemicals in the same setting?. Environ Res.

[28] Hernandez JP, Chapman LM, Kretschmer XC, Baldwin WS (2006). Gender-specific induction of cytochrome P450s in nonylphenol-treated FVB/NJ mice. Toxicol Appl Pharmacol.

[29] Mačak-Šafranko Ž, Sobočanec S, Šarić A, Balog T, Šverko V, Kušić B (2011). Cytochrome P450 gender-related differences in response to hyperoxia in young CBA mice. Exp Toxicol Pathol.

[30] Franconi F, Sanna M, Straface E, Chessa R, Rosano G. Pharmacokinetics and Pharmacodynamics: The Role of Sex and Gender. In: Sex and Gender Aspects in Clinical Medicine. London: Springer; 2012. p. 183–94.

[31] Wang L, Ahn YJ, Asmis R (2020). Sexual dimorphism in glutathione metabolism and glutathione-dependent responses. Redox Biol.

[32] Wizemann TM, Pardue ML. Exploring the biological contributions to human health: Does sex matter? National Academy Press; 2021. 25057540

[33] Klein W, Gochfeld M, Davis B. Background on the Scientific Group on Methodologies for the Safety Evaluation of Chemicals and Workshop 16: Gender differences. Environ Res.2007;104(1):2–3.

[34] Llop S, Lopez-Espinosa MJ, Rebagliato M, Ballester F (2013). Gender differences in the neurotoxicity of metals in children. Toxicology.

[35] Bauer JA, Fruh V, Howe CG, White RF, Claus HB (2020). Associations of Metals and Neurodevelopment: a Review of Recent Evidence on Susceptibility Factors. Curr Epidemiol Rep.

[36] Zhang Q, Chen XZ, Huang X, Wang M, Wu J (2019). The association between prenatal exposure to phthalates and cognition and neurobehavior of children-evidence from birth cohorts. Neurotoxicology.

[37] Neisser U, Boodoo G, Bouchard TJ, Boykin AW, Brody N, Ceci SJ (1996). Intelligence: Knowns and unknowns. Am Psychol.

[38] Seltzer MM, Floyd F, Greenberg J, Lounds J, Lindstromm M, Hong J. Life course impacts of mild intellectual deficits. Am J Ment Retard. 2005;110(6):451–68. 10.1352/0895-8017(2005)110[451:LCIOMI]2.0.CO;2 16212448

[39] Morgan RL, Whaley P, Thayer KA, Schünemann HJ (2018). Identifying the PECO: A framework for formulating good questions to explore the association of environmental and other exposures with health outcomes. Environ Int.

[40] Eick SM, Goin DE, Chartres N, Lam J, Woodruff TJ (2020). Assessing risk of bias in human environmental epidemiology studies using three tools: different conclusions from different tools. Syst Rev.

[41] Woodruff TJ, Sutton P. The navigation guide systematic review methodology: A rigorous and transparent method for translating environmental health science into better health outcomes. Environ Health Perspect. 2014;122(10):1007–14. 10.1289/ehp.1307175 PMC4181919 24968373

[42] Balshem H, Helfand M, Schünemann HJ, Oxman AD, Kunz R, Brozek J (2011). GRADE guidelines: 3. Rating the quality of evidence. J Clin Epidemiol.

[43] Navigation Guide Protocol for Rating the Quality and Strength of Human and Non‐Human Evidence [Internet]. University of California, San Francisco, Program on Reproductive Health and the Environment; 2012. Available from: https://prhe.ucsf.edu/sites/g/files/tkssra341/f/Instructions%20to%20Authors%20for%20GRADING%20QUALITY%20OF%20EVIDENCE.pdf

[44] Karr SK, Carvajal H, Elser D, Bays K, Logan RA, Page GL (1993). Concurrent validity of the wppsi-r and the McCARTHY scales of children’s abilities. Psychol Rep.

[45] Moore C, O’Keefe SL, Lawhon D, Tellegen P (1998). Concurrent validity of the snijders-oomen nonverbal intelligence test 2 1/2–7–revised with the Wechsler preschool and primary scale of intelligence–revised. Psychol Rep.

[46] Rodríguez-Barranco M, Tobías A, Redondo D, Molina-Portillo E, Sánchez MJ. Standardizing effect size from linear regression models with log-transformed variables for meta-analysis. BMC Med Res Methodol. 2017;17(1):1–9. 10.1186/s12874-017-0322-8 PMC5356327 28302052

[47] DerSimonian R, Laird N (1986). Meta-analysis in clinical trials. Control Clin Trials.

[48] Higgins JPT (2003). Measuring inconsistency in meta-analyses. BMJ.

[49] Egger M, Smith GD, Schneider M, Minder C (1997). Bias in meta-analysis detected by a simple, graphical test. BMJ.

[50] Viechtbauer W, Cheung MWL (2010). Outlier and influence diagnostics for meta-analysis. Res Synth Methods.

[51] Berghuis SA, Van Braeckel KNJA, Sauer PJJ, Bos AF (2018). Prenatal exposure to persistent organic pollutants and cognition and motor performance in adolescence. Environ Int.

[52] Freire C, Amaya E, Gil F, Fernández MF, Murcia M, Llop S (2018). Prenatal co-exposure to neurotoxic metals and neurodevelopment in preschool children: The Environment and Childhood (INMA) Project. Sci Total Environ.

[53] Guo J, Wu C, Zhang J, Qi X, Lv S, Jiang S (2020). Prenatal exposure to mixture of heavy metals, pesticides and phenols and IQ in children at 7 years of age: The SMBCS study. Environ Int.

[54] Tatsuta N, Nakai K, Kasanuma Y, Iwai-Shimada M, Sakamoto M, Murata K (2020). Prenatal and postnatal lead exposures and intellectual development among 12-year-old Japanese children. Environ Res.

[55] Azar N, Booij L, Muckle G, Arbuckle TE, Séguin JR, Asztalos E (2021). Prenatal exposure to polybrominated diphenyl ethers (PBDEs) and cognitive ability in early childhood. Environ Int.

[56] Baghurst PA, McMichael AJ, Wigg NR, Vimpani GV, Robertson EF, Roberts RJ (1992). Environmental exposure to lead and children’s intelligence at the age of seven years: The Port Pirie Cohort Study. N Engl J Med.

[57] Bouchard MF, Chevrier J, Harley KG, Kogut K, Vedar M, Calderon N (2011). Prenatal exposure to organophosphate pesticides and IQ in 7-year-old children. Environ Health Perspect.

[58] Castorina R, Bradman A, Stapleton HM, Butt C, Avery D, Harley KG (2017). Current-use flame retardants: Maternal exposure and neurodevelopment in children of the CHAMACOS cohort. Chemosphere.

[59] Damm D, Grandjean P, Lyngbye T, Trillingsgaard A, Hansen ON (1993). Early lead exposure and neonatal jaundice: Relation to neurobehavioral performance at 15 years of age. Neurotoxicol Teratol.

[60] Davidson PW, Myers GJ, Cox C, Axtell C, Shamlaye C, Sloane-Reeves J (1998). Effects of prenatal and postnatal methylmercury exposure from fish consumption on neurodevelopment: Outcomes at 66 months of age in the Seychelles Child Development Study. JAMA J Am Med Assoc.

[61] Davidson PW, Myers GJ, Shamlaye C, Cox C, Wilding GE (2004). Prenatal exposure to methylmercury and child development: influence of social factors. Neurotoxicol Teratol.

[62] Davidson PW, Myers GJ, Cox C, Wilding GE, Shamlaye CF, Huang LS (2006). Methylmercury and neurodevelopment: Longitudinal analysis of the Seychelles child development cohort. Neurotoxicol Teratol.

[63] Desrochers-Couture M, Oulhote Y, Arbuckle TE, Fraser WD, Séguin JR, Ouellet E (2018). Prenatal, concurrent, and sex-specific associations between blood lead concentrations and IQ in preschool Canadian children. Environ Int.

[64] Dietrich KN, Berger OG, Succop PA, Hammond PB, Bornschein RL (1993). The developmental consequences of low to moderate prenatal and postnatal lead exposure: Intellectual attainment in the Cincinnati Lead Study Cohort following school entry. Neurotoxicol Teratol.

[65] Ernhart CB, Morrow-Tlucak M, Wolf AW, Super D, Drotar D (1989). Low level lead exposure in the prenatal and early preschool periods: Intelligence prior to school entry. Neurotoxicol Teratol.

[66] Eskenazi B, Chevrier J, Rauch SA, Kogut K, Harley KG, Johnson C (2013). In utero and childhood polybrominated diphenyl ether (PBDE) exposures and neurodevelopment in the CHAMACOS study. Environ Health Perspect.

[67] Factor-Litvak P, Insel B, Calafat AM, Liu X, Perera F, Rauh VA (2014). Persistent Associations between Maternal Prenatal Exposure to Phthalates on Child IQ at Age 7 Years. PLoS ONE.

[68] Furlong MA, Herring A, Buckley JP, Goldman BD, Daniels JL, Engel LS (2017). Prenatal exposure to organophosphorus pesticides and childhood neurodevelopmental phenotypes. Environ Res.

[69] Gascon M, Valvi D, Forns J, Casas M, Martínez D, Júlvez J (2015). Prenatal exposure to phthalates and neuropsychological development during childhood. Int J Hyg Environ Health.

[70] Golding J, Hibbeln JR, Gregory SM, Iles-Caven Y, Emond A, Taylor CM (2017). Maternal prenatal blood mercury is not adversely associated with offspring IQ at 8 years provided the mother eats fish: A British prebirth cohort study. Int J Hyg Environ Health.

[71] Huang LS, Myers GJ, Davidson PW, Cox C, Xiao F, Thurston SW (2007). Is susceptibility to prenatal methylmercury exposure from fish consumption non-homogeneous? Tree-structured analysis for the Seychelles Child Development Study. Neurotoxicology.

[72] Huang HB, Chen HY, Su PH, Huang PC, Sun CW, Wang CJ (2015). Fetal and Childhood Exposure to Phthalate Diesters and Cognitive Function in Children Up to 12 Years of Age: Taiwanese Maternal and Infant Cohort Study. PLoS ONE.

[73] Hyland C, Mora AM, Kogut K, Calafat AM, Harley K, Deardorff J (2019). Prenatal Exposure to Phthalates and Neurodevelopment in the CHAMACOS Cohort. Environ Health Perspect.

[74] Ikeno T, Miyashita C, Nakajima S, Kobayashi S, Yamazaki K, Saijo Y (2018). Effects of low-level prenatal exposure to dioxins on cognitive development in Japanese children at 42months. Sci Total Environ.

[75] Jacobson JL, Jacobson SW (2002). Breast-feeding and gender as moderators of teratogenic effects on cognitive development. Neurotoxicol Teratol.

[76] Julvez J, Smith GD, Golding J, Ring S, Pourcain BS, Gonzalez JR (2013). Prenatal methylmercury exposure and genetic predisposition to cognitive deficit at age 8 years. Epidemiol Camb Mass.

[77] Jusko TA, van den Dries MA, Pronk A, Shaw PA, Guxens M, Spaan S (2019). Organophosphate Pesticide Metabolite Concentrations in Urine during Pregnancy and Offspring Nonverbal IQ at Age 6 Years. Environ Health Perspect.

[78] Kyriklaki A, Vafeiadi M, Kampouri M, Koutra K, Roumeliotaki T, Chalkiadaki G (2016). Prenatal exposure to persistent organic pollutants in association with offspring neuropsychological development at 4years of age: The Rhea mother-child cohort, Crete. Greece Environ Int.

[79] Li N, Papandonatos GD, Calafat AM, Yolton K, Lanphear BP, Chen A (2019). Identifying periods of susceptibility to the impact of phthalates on children’s cognitive abilities. Environ Res.

[80] Llop S, Ballester F, Murcia M, Forns J, Tardon A, Andiarena A (2017). Prenatal exposure to mercury and neuropsychological development in young children: the role of fish consumption. Int J Epidemiol.

[81] McBride WG, Black BP, English BJ (1982). Blood lead levels and behaviour of 400 preschool children. Med J Aust.

[82] McMichael AJ, Baghurst PA, Vimpani GV, Wigg NR, Robertson EF, Tong S (1994). Tooth lead levels and IQ in school-age children: the Port Pirie Cohort Study. Am J Epidemiol.

[83] McMichael AJ, Baghurst PA, Vimpani GV, Robertson EF, Wigg NR, Tong SL (1992). Sociodemographic factors modifying the effect of environmental lead on neuropsychological development in early childhood. Neurotoxicol Teratol.

[84] McMichael AJ, Baghurst PA, Wigg NR, Vimpani GV, Robertson EF, Roberts RJ (1988). Port Pirie Cohort Study: environmental exposure to lead and children’s abilities at the age of four years. N Engl J Med.

[85] Min MO, Singer LT, Kirchner HL, Minnes S, Short E, Hussain Z (2009). Cognitive development and low-level lead exposure in poly-drug exposed children. Neurotoxicol Teratol.

[86] Myers GJ, Davidson PW, Cox C, Shamlaye CF, Palumbo D, Cernichiari E (2003). Prenatal methylmercury exposure from ocean fish consumption in the Seychelles child development study. The Lancet.

[87] Myers GJ, Davidson PW, Cox C, Shamlaye CF, Tanner MA, Choisy O (1995). Neurodevelopmental outcomes of Seychellois children sixty-six months after in utero exposure to methylmercury from a maternal fish diet: pilot study. Neurotoxicology.

[88] Ntantu Nkinsa P, Muckle G, Ayotte P, Lanphear BP, Arbuckle TE, Fraser WD (2020). Organophosphate pesticides exposure during fetal development and IQ scores in 3 and 4-year old Canadian children. Environ Res.

[89] Palumbo DR, Cox C, Davidson PW, Myers GJ, Choi A, Shamlaye C (2000). Association between prenatal exposure to methylmercury and cognitive functioning in Seychellois children: a reanalysis of the McCarthy Scales of Children’s Ability from the main cohort study. Environ Res.

[90] Pocock SJ, Ashby D, Smith MA (1987). Lead exposure and children’s intellectual performance. Int J Epidemiol.

[91] Rauh V, Arunajadai S, Horton M, Perera F, Hoepner L, Barr DB (2011). Seven-year neurodevelopmental scores and prenatal exposure to chlorpyrifos, a common agricultural pesticide. Environ Health Perspect.

[92] Ris MD, Dietrich KN, Succop PA, Berger OG, Bornschein RL (2004). Early exposure to lead and neuropsychological outcome in adolescence. J Int Neuropsychol Soc.

[93] Schnaas L, Rothenberg SJ, Perroni E, Martínez S, Hernández C, Hernández RM (2000). Temporal pattern in the effect of postnatal blood lead level on intellectual development of young children. Neurotoxicol Teratol.

[94] Tatsuta N, Nakai K, Murata K, Suzuki K, Iwai-Shimada M, Kurokawa N (2014). Impacts of prenatal exposures to polychlorinated biphenyls, methylmercury, and lead on intellectual ability of 42-month-old children in Japan. Environ Res.

[95] Taylor CM, Kordas K, Golding J, Emond AM (2017). Effects of low-level prenatal lead exposure on child IQ at 4 and 8 years in a UK birth cohort study. Neurotoxicology.

[96] Torres-Olascoaga LA, Watkins D, Schnaas L, Meeker JD, Solano-Gonzalez M, Osorio-Valencia E, et al. Early Gestational Exposure to High-Molecular-Weight Phthalates and Its Association with 48-Month-Old Children’s Motor and Cognitive Scores. Int J Environ Res Public Health. 2020;17(21):8150. 10.3390/ijerph17218150 PMC7662459 33158190

[97] van den Dries MA, Guxens M, Pronk A, Spaan S, El Marroun H, Jusko TA (2019). Organophosphate pesticide metabolite concentrations in urine during pregnancy and offspring attention-deficit hyperactivity disorder and autistic traits. Environ Int.

[98] Vuong AM, Yolton K, Xie C, Webster GM, Sjödin A, Braun JM (2017). Childhood polybrominated diphenyl ether (PBDE) exposure and neurobehavior in children at 8 years. Environ Res.

[99] Zhu YD, Wu XY, Yan SQ, Huang K, Tong J, Gao H (2020). Domain- and trimester-specific effect of prenatal phthalate exposure on preschooler cognitive development in the Ma’anshan Birth Cohort (MABC) study. Environ Int.

[100] Choi G, Villanger GD, Drover SSM, Sakhi AK, Thomsen C, Nethery RC (2021). Prenatal phthalate exposures and executive function in preschool children. Environ Int.

[101] Huang PC, Su PH, Chen HY, Huang HB, Tsai JL, Huang HI (2012). Childhood blood lead levels and intellectual development after ban of leaded gasoline in Taiwan: a 9-year prospective study. Environ Int.

[102] Chen A, Yolton K, Rauch SA, Webster GM, Hornung R, Sjödin A (2014). Prenatal polybrominated diphenyl ether exposures and neurodevelopment in U.S. children through 5 years of age: the HOME study. Environ Health Perspect.

[103] Sandman CA, Glynn LM, Davis EP (2013). Is there a viability–vulnerability tradeoff? Sex differences in fetal programming. J Psychosom Res.

[104] Sood R, Zehnder JL, Druzin ML, Brown PO (2006). Gene expression patterns in human placenta. Proc Natl Acad Sci U S A.

[105] Buckberry S, Bianco-Miotto T, Bent SJ, Dekker GA, Roberts CT (2014). Integrative transcriptome metaanalysis reveals widespread sex-biased gene expression at the human fetal-maternal interface. Mol Hum Reprod.

[106] Eriksson JG, Kajantie E, Osmond C, Thornburg K, Barker DJP (2010). Boys live dangerously in the womb. Am J Hum Biol.

[107] Bale TL (2016). The placenta and neurodevelopment: sex differences in prenatal vulnerability. Dialogues Clin Neurosci.

[108] Rosenfeld CS (2015). Sex-Specific Placental Responses in Fetal Development. Endocrinology.

[109] Kelley AS, Banker M, Goodrich JM, Dolinoy DC, Burant C, Domino SE (2019). Early pregnancy exposure to endocrine disrupting chemical mixtures are associated with inflammatory changes in maternal and neonatal circulation. Sci Rep.

[110] Boskabady M, Marefati N, Farkhondeh T, Shakeri F, Farshbaf A, Boskabady MH (2018). The effect of environmental lead exposure on human health and the contribution of inflammatory mechanisms, a review. Environ Int.

[111] Barrientos RM, Brunton PJ, Lenz KM, Pyter L, Spencer SJ (2019). Neuroimmunology of the female brain across the lifespan: Plasticity to psychopathology. Brain Behav Immun.

[112] Makinson R, Lloyd K, Rayasam A, McKee S, Brown A, Barila G (2017). Intrauterine inflammation induces sex-specific effects on neuroinflammation, white matter, and behavior. Brain Behav Immun.

[113] Ardalan M, Chumak T, Vexler Z, Mallard C (2019). Sex-Dependent Effects of Perinatal Inflammation on the Brain: Implication for Neuro-Psychiatric Disorders. Int J Mol Sci.

[114] Na Q, Chudnovets A, Liu J, Lee JY, Dong J, Shin N (2021). Placental Macrophages Demonstrate Sex-Specific Response to Intrauterine Inflammation and May Serve as a Marker of Perinatal Neuroinflammation. J Reprod Immunol.

[115] Minghetti L, Greco A, Zanardo V, Suppiej A (2013). Early-life sex-dependent vulnerability to oxidative stress: the natural twining model. J Matern Fetal Neonatal Med.

[116] Demarest TG, McCarthy MM (2015). Sex differences in mitochondrial (dys)function: Implications for neuroprotection. J Bioenerg Biomembr.

[117] Di Marco BM, Bonaccorso C, Aloisi E, D’Antoni SV., Catania M (2016). Neuro-Inflammatory Mechanisms in Developmental Disorders Associated with Intellectual Disability and Autism Spectrum Disorder: A Neuro- Immune Perspective. CNS Neurol Disord - Drug Targets.

[118] Wells PG, McCallum GP, Chen CS, Henderson JT, Lee CJJ, Perstin J (2009). Oxidative Stress in Developmental Origins of Disease: Teratogenesis, Neurodevelopmental Deficits, and Cancer. Toxicol Sci.

[119] Buss C, Davis EP, Class QA, Gierczak M, Pattillo C, Glynn LM (2009). Maturation of the human fetal startle response: Evidence for sex-specific maturation of the human fetus. Early Hum Dev.

[120] Azcoitia I, Barreto GE, Garcia-Segura LM (2019). Molecular mechanisms and cellular events involved in the neuroprotective actions of estradiol Analysis of sex differences. Front Neuroendocrinol.

[121] Torres-Rojas C, Jones BC (2018). Sex differences in neurotoxicogenetics. Front Genet.

[122] Schumacher M, Liere P, Ghoumari A (2020). Progesterone and fetal-neonatal neuroprotection. Best Pract Res Clin Obstet Gynaecol.

[123] Govender P, Ghai M, Okpeku M (2022). Sex-specific DNA methylation: impact on human health and development. Mol Genet Genomics.

[124] Lister R, Mukamel EA, Nery JR, Urich M, Puddifoot CA, Johnson ND, et al. Global epigenomic reconfiguration during mammalian brain development. Science. 2013;341(6146):1237905. 10.1126/science.1237905 PMC3785061 23828890

[125] Singh G, Singh V, Wang ZX, Voisin G, Lefebvre F, Navenot JM (2018). Effects of developmental lead exposure on the hippocampal methylome: Influences of sex and timing and level of exposure. Toxicol Lett.

[126] Cardenas A, Rifas-Shiman SL, Agha G, Hivert MF, Litonjua AA, DeMeo DL (2017). Persistent DNA methylation changes associated with prenatal mercury exposure and cognitive performance during childhood. Sci Rep.

[127] Kim S, Cho YH, Won S, Ku JL, Moon HB, Park J (2019). Maternal exposures to persistent organic pollutants are associated with DNA methylation of thyroid hormone-related genes in placenta differently by infant sex. Environ Int.

[128] Geary DC (2017). Evolution of Human Sex-Specific Cognitive Vulnerabilities. Q Rev Biol.

[129] Geary DC (2021). Now you see them, and now you don’t: An evolutionarily informed model of environmental influences on human sex differences. Neurosci Biobehav Rev.

[130] Ruigrok ANV, Salimi-Khorshidi G, Lai MC, Baron-Cohen S, Lombardo MV, Tait RJ (2014). A meta-analysis of sex differences in human brain structure. Neurosci Biobehav Rev.

[131] Lee ACH, Yeung LK, Barense MD (2012). The hippocampus and visual perception. Front Hum Neurosci.

[132] Borsani E, Della Vedova AM, Rezzani R, Rodella LF, Cristini C (2019). Correlation between human nervous system development and acquisition of fetal skills: An overview. Brain Dev.

[133] Caito SW, Aschner M (2015). Mitochondrial Redox Dysfunction and Environmental Exposures. Antioxid Redox Signal.

[134] Zhang T, Yin X, Chen H, Li Y, Chen J, Yang X (2022). Global magnitude and temporal trends of idiopathic developmental intellectual disability attributable to lead exposure from 1990 to 2019: Results from Global Burden of Disease Study. Sci Total Environ.

[135] Rees N, Fuller R. The Toxic Truth: Children’s Exposure to Lead Pollution Undermines a Generation of Future Potential. UNICEF; 2020.

[136] Bellinger DC, Matthews-Bellinger JA, Kordas K (2016). A developmental perspective on early-life exposure to neurotoxicants. Environ Int.

[137] Bellinger D, Leviton A, Waternaux C (1989). Lead, IQ and social class. Int J Epidemiol.

[138] Cory-Slechta DA, Virgolini MB, Thiruchelvam M, Weston DD, Bauter MR (2004). Maternal stress modulates the effects of developmental lead exposure. Environ Health Perspect.

[139] Sobolewski M, Abston K, Conrad K, Marvin E, Harvey K, Susiarjo M (2020). Lineage-and sex-dependent behavioral and biochemical transgenerational consequences of developmental exposure to lead, prenatal stress, and combined lead and prenatal stress in mice. Environ Health Perspect.

[140] Sobolewski M, Varma G, Adams B, Anderson DW, Schneider JS, Cory-Slechta DA (2018). Developmental lead exposure and prenatal stress result in sex-specific reprograming of adult stress physiology and epigenetic profiles in brain. Toxicol Sci.

[141] Varma G, Sobolewski M, Cory-Slechta DA, Schneider JS (2017). Sex- and brain region- specific effects of prenatal stress and lead exposure on permissive and repressive post-translational histone modifications from embryonic development through adulthood. Neurotoxicology.

[142] Myers GJ, Davidson PW, Strain JJ (2007). Nutrient and methyl mercury exposure from consuming fish. J Nutr.

[143] Pacyga DC, Gardiner JC, Flaws JA, Li Z, Calafat AM, Korrick SA (2021). Maternal phthalate and phthalate alternative metabolites and urinary biomarkers of estrogens and testosterones across pregnancy. Environ Int.

[144] Winn HN, Chervenak FA, Romero R. Clinical Maternal-Fetal Medicine Online. 2nd ed. London: CRC Press; 2021. Available from: https://www.taylorfrancis.com/books/9781003222590. Cited 2022 Nov 11.

[145] Ruben RJ (1997). A Time Frame of Critical/Sensitive Periods of Language Development. Acta Otolaryngol (Stockh).

[146] Bellinger DC. Environmental Chemical Exposures and Intellectual Disability in Children. In: Matson JL, editor. Handbook of Intellectual Disabilities. Cham: Springer International Publishing; 2019 [cited 2022 Nov 11]. p. 347–63. (Autism and Child Psychopathology Series). Available from: 10.1007/978-3-030-20843-1_20

[147] Grandjean P (2019). Developmental fluoride neurotoxicity: an updated review. Environ Health Glob Access Sci Source.

[148] Galiciolli MEA, Lima LS, da Costa N de S, de Andrade DP, Irioda AC, Oliveira CS (2022). IQ alteration induced by lead in developed and underdeveloped/developing countries: A systematic review and a meta-analysis. Environ Pollut.

[149] Fuhrmann D, Knoll LJ, Blakemore SJ (2015). Adolescence as a Sensitive Period of Brain Development. Trends Cogn Sci.

[150] Lenroot RK, Gogtay N, Greenstein DK, Wells EM, Wallace GL, Clasen LS (2007). Sexual dimorphism of brain developmental trajectories during childhood and adolescence. Neuroimage.

[151] Jalnapurkar I, Allen M, Pigott T (2018). Sex Differences in Anxiety Disorders: A Review. Psychiatry Depress Anxiety.

[152] Barrett ES, Padula AM (2019). Joint Impact of Synthetic Chemical and Non-chemical Stressors on Children’s Health. Curr Environ Health Rep.

[153] Gaylord A, Osborne G, Ghassabian A, Malits J, Attina T, Trasande L (2020). Trends in neurodevelopmental disability burden due to early life chemical exposure in the USA from 2001 to 2016: A population-based disease burden and cost analysis. Mol Cell Endocrinol.

