# Supplementary material for: Sex difference of pre- and post-natal exposure to six developmental neurotoxicants on intellectual abilities: a systematic review and meta-analysis of human studies
Source: Environ Health. 2023 Nov 17;22:80. doi: 10.1186/s12940-023-01029-z (PMC10655280; doi:10.1186/s12940-023-01029-z)
Supplement: Supplementary file 1 — Additional file 1: Appendices Appendix A. Risk of Bias Instructions. Appendix B. Effect Size Transformation. [file 12940_2023_1029_MOESM1_ESM.docx]

**Appendices**

**Appendix A: Risk of Bias Instructions**

***Please answer LOW RISK, PROBABLY LOW RISK, PROBABLY HIGH RISK, or HIGH RISK.***

*These questions are adapted from those used in the Navigation Guide systematic review of PBDEs and neurodevelopmental effects*^16^*.*

*An answer of “Yes” aligns with a rating of “Low risk of bias,” “Probably Yes” aligns with a rating of “Probably low risk of bias,” “Probably No” aligns with a rating of “Probably high risk of bias”, and “No” aligns with a rating of “High risk of bias.”*

**1. Did the study sample adequately represent their source population such that the risk of selection effects was minimal?**

Criteria for a judgment of LOW risk of bias (i.e., answer: “Yes”):

EITHER:

a) The descriptions of the source population, inclusion/exclusion criteria, recruitment and enrollment procedures, participation and follow-up rates were sufficiently detailed, and adequate data were supplied on the distribution of relevant study sample and population characteristics to support the assertion that risk of selection effects was minimal.

OR

b) Although the descriptions and/or data as indicated in “a” above suggested the potential for selection effects, adequate support was given indicating that potential selection effects were *not* differential across both exposure and outcome.

OR

c) Although the descriptions and/or data as indicated in “a” above suggested the potential for selection effects and there was no support indicating that potential selection effects were *not* differential across both exposure and outcome, selection factors appeared to be well-understood, were measured in the data set, and appropriate adjustment post hoc techniques were used to control for selection bias.

Criteria for the judgment of PROBABLY LOW risk of bias (i.e., answer: “Probably Yes”):

There is insufficient information about participant selection to permit a judgment of low risk of bias, but there is indirect evidence which suggests that inclusion/exclusion criteria, recruitment and enrollment procedures, and participation and follow-up rates were consistent across groups as described by the criteria for a judgment of low risk of bias.

Criteria for the judgment of PROBABLY HIGH risk of bias (i.e., answer: “Probably No”):

There is insufficient information about participant selection to permit a judgment of high risk of bias, but there is indirect evidence which suggests that inclusion/exclusion criteria, recruitment and enrollment procedures, and participation and follow-up rates were inconsistent across groups, as described by the criteria for a judgment of high risk of bias.

Criteria for the judgment of HIGH risk of bias (i.e., answer: “No”):

1. There were indications from descriptions of the source population, inclusion/exclusion criteria, recruitment and enrollment procedures, participation and follow-up rates, or data on the distribution of relevant study sample and population characteristics that risk of selection effects were substantial; and
2. There was no support to indicate that potential selection effects were *not* differential across both exposure and outcome; and
3. Adjustment post hoc techniques were not used to control for selection bias.

**2. Was knowledge of the exposure or outcome adequately prevented (i.e., blinded or masked) during the study?**

Criteria for a judgment of LOW risk of bias (i.e., answer: “Yes”):

Any of the following:

- Blinding of key study personnel was ensured, and it is unlikely that the blinding could have been broken; or
- Some key study personnel were not blinded, but exposure and outcome assessment were blinded and the non-blinding of others is unlikely to introduce bias.

Criteria for the judgment of PROBABLY LOW risk of bias (i.e., answer: “Probably Yes”):

There is insufficient information about blinding to permit a judgment of low risk of bias, but there is indirect evidence which suggests the study was adequately blinded, as described by the criteria for a judgment of low risk of bias. Or the review authors judge that the outcome measures as well as the exposure measures are not likely to be influenced by lack of blinding. For example, if the exposure was measured by a separate entity and the outcome was obtained from a hospital record.

Criteria for the judgment of PROBABLY HIGH risk of bias (i.e., answer: “Probably No”):

There is insufficient information about blinding to permit a judgment of high risk of bias, but there is indirect evidence which suggests the study was not adequately blinded, as described by the criteria for a judgment of high risk of bias.

Criteria for the judgment of HIGH risk of bias (i.e., answer: “No”):

Any of the following:

- No blinding or incomplete blinding, and the outcome measures or exposure measures is likely to be influenced by lack of blinding (i.e., differential outcome or exposure assessment); or
- Blinding of key study personnel attempted, but likely that the blinding could have been broken so as to introduce bias; or
- Some key study personnel were not blinded, and the non-blinding of others was likely to introduce bias.

**3. Is there high confidence in the accuracy of the exposure assessment methods?**

Criteria for a judgment of LOW risk of bias (i.e., answer: “Yes”):

The reviewers judge that there is low risk of exposure misclassification, i.e.,:

- There is high confidence in the accuracy of the exposure assessment methods, such as methods that have been tested for validity and reliability in measuring the targeted exposure;
  - Lead – measured in whole blood, taking the mean of serial measurements, or in circumpulpal dentin^146^
  - Mercury – measured in hair or whole blood for acute exposure^147^
  - PCBs – measured in serum or breastmilk shortly after birth and lipid adjusted ^148,149^
  - PBDEs – measured in serum or plasma for prenatal exposure, or in breastmilk for postnatal exposure and lipid adjusted^150,151^
  - OP Pesticides – measured in urine and adjusted for urinary dilution or in blood with multiple measurements^152^
  - Phthalates – measured in multiple urine spot samples and adjusted for urinary dilution through specific gravity^153^.
- or
- Less-established or less direct exposure measurements are validated against well-established or direct methods

AND

- Appropriate QA/QC for methods are described and are satisfactory, with at least three of the following items reported, or at least two of the following items reported plus evidence of satisfactory performance in a high-quality inter‐laboratory comparison:
  - Limit of detection (LoD) or quantification (with fewer than 10% of the observations being below the LoD)^154^
  - standards recovery;
  - measure of repeatability;
  - investigation and prevention of blanks contamination.

Criteria for the judgment of PROBABLY LOW risk of bias (i.e., answer: “Probably Yes”):

- There may be insufficient information about the exposure assessment methods to permit a judgement of low risk of bias but there is indirect evidence, which suggests that methods were robust, as described by the criteria for a judgement of low risk of bias. For example, studies reporting that the QA/QC items above were satisfactory but not reporting all the actual numbers may receive a judgement of “probably low risk of bias”.

Criteria for the judgment of PROBABLY HIGH risk of bias (i.e., answer: “Probably No”):

- There is insufficient information about the exposure assessment methods to permit a judgment of high risk of bias, but there is indirect evidence which suggests that methods were not robust, as described by the criteria for a judgment of high risk of bias.

Criteria for the judgment of HIGH risk of bias (i.e., answer: “No”):

The reviewers judge that there is high risk of exposure misclassification and any one of the following:

- There is low confidence in the accuracy of the exposure assessment methods; or
- Less established or less direct exposure measurements were not validated and are suspected to introduce bias that impacts the outcome assessment (example: participants are asked to report exposure status retrospectively, subject to recall bias)
- Uncertain how exposure information was obtained

**4. Is there high confidence in the accuracy of the outcome assessment methods?**

Criteria for a judgment of LOW risk of bias (i.e., answer: “Yes”):

The reviewers judge that there is low risk of outcome misclassification, i.e.:

- Outcomes were assessed and defined consistently across all study participants, using valid and reliable measures of intelligence; or
  - - For example, assessed via the McCarthy Scales of Children’s Abilities (MCSA), the Wechsler Preschool and Primary Scale of Intelligence (WPPSI), the Wechsler Intelligence Scale for Children (WISC), or the Kaufman Assessment Battery for Children (K-ABC).
- Less established or less direct outcome measurements are validated against well-established or direct methods; or
- Appropriate sensitivity analyses were conducted that suggest the influence of outcome misclassification would be minimal
- AND, if applicable, appropriate QA/QC for methods is described and is satisfactory.

Criteria for the judgment of PROBABLY LOW risk of bias (i.e., answer: “Probably Yes”):

There is insufficient information about the outcome assessment methods to permit a judgment of low risk of bias, but there is indirect evidence which suggests that methods were robust, as described by the criteria for a judgment of low risk of bias. Appropriate QA/QC for methods are not described but the review authors judge that the outcome and the outcome assessment are objective and uniform across all study participants.

Criteria for the judgment of PROBABLY HIGH risk of bias (i.e., answer: “Probably No”):

There is insufficient information about the outcome assessment methods to permit a judgment of high risk of bias, but there is indirect evidence which suggests that methods were not robust, as described by the criteria for a judgment of high risk of bias.

Criteria for the judgment of HIGH risk of bias (i.e., answer: “No”):

The reviewers judge that there is high risk of outcome misclassification and any one of the following:

- There is low confidence in the accuracy of the outcome assessment methods; or
- Less established or less direct outcome measurements are not validated and are suspected to introduce bias that impacts the outcome assessment
- Uncertain how outcome information was obtained

**5. Were potential confounders adequately considered?**

List of important potential confounders, collectively generated by review authors prior to the initiation of screening for studies based on expert opinion and knowledge gathered from the literature:

Tier I: Important confounders

- maternal IQ/education
- SES-based measure (e.g., socioeconomic status, parental occupation/employment, household income, or income-to-needs ratio, poverty-to-income ratio,)
- Home Observation for Measure of the Environment (HOME) score (Caldwell & Bradley, 1984); or Family Assessment Measure (FAM; Skinner, Steinhauer, & Santa-Barbara, 1983); or another acceptable measure of HOME environment
- Race/ethnicity
- Centre/city if it’s multi-site study
- Fish intake if the exposure is mercury

Tier II: Other potentially important confounders:

- Exposure to at least one other neurotoxic agents (e.g., pesticides, mercury, lead, air pollutants)
- Exposure to tobacco smoke during pregnancy
- Maternal age
- Marital status
- Maternal alcohol intake
- Maternal depression or stress/mood disorder/psychopathology

Other variables for consideration – could be exclusions or confounders:

- Low birth weight, preterm birth, or other birth complications
- Parental drug use
- Serious head injury (exclusion)

Criteria for a judgment of LOW risk of bias (i.e., answer: “Yes”):

The study appropriately assessed and considered or accounted for all important confounders^[[1]](#footnote-1)^ (Tier I), or reported that important confounders were evaluated and omitted because inclusion did not substantially affect the results.

AND the study appropriately assessed and considered or accounted for most but not necessarily all other potentially important confounders relevant (Tier II), or reported that these confounders were evaluated and omitted because inclusion did not substantially affect the results,

AND the important potential confounders were measured consistently across study groups using valid and reliable methods, or the influence of covariate measurement error was determined, through sensitivity analysis, to be minimal.

Criteria for the judgment of PROBABLY LOW risk of bias (i.e., answer: “Probably Yes”):

The study appropriately assessed and considered or accounted for most but not all the important confounders (Tier I),

AND this is not expected to introduce substantial bias.

Criteria for the judgment of PROBABLY HIGH risk of bias (i.e., answer: “Probably No”):

The study evaluated some but not all the important confounders (Tier I),

AND some but not all the other potentially important confounders relevant (Tier II),

AND this is expected to introduce substantial bias.

Criteria for the judgment of HIGH risk of bias (i.e., answer: “No”):

The study did not account for or evaluate multiple important confounders (Tier I),

AND did not account for or evaluate multiple other potentially important confounders relevant (Tier II),

OR the important potential confounders were inappropriately measured and/or inappropriately analyzed across study groups.

**6. Were incomplete outcome data adequately addressed?**

Criteria for a judgment of LOW risk of bias (i.e., answer: “Yes”):

Participants were followed long enough to obtain outcome measurements

OR any one of the following:

- No missing outcome data; or
- Reasons for missing outcome data unlikely to be related to true outcome (for survival data, censoring unlikely to introduce bias); or
- Attrition or missing outcome data balanced in numbers across exposure groups, with similar reasons for missing data across groups; or
- For dichotomous outcome data, the proportion of missing outcomes compared with observed event risk not enough to have a relevant impact on the exposure effect estimate; or
- For continuous outcome data, plausible effect size (difference in means or standardized difference in means) among missing outcomes not enough to have a relevant impact on the observed effect size; or
- Missing data have been imputed using appropriate methods

Criteria for the judgment of PROBABLY LOW risk of bias (i.e., answer: “Probably Yes”):

There is insufficient information about incomplete outcome data to permit a judgment of low risk of bias, but there is indirect evidence which suggests incomplete outcome data was adequately addressed, as described by the criteria for a judgment of low risk of bias.

Criteria for the judgment of PROBABLY HIGH risk of bias (i.e., answer: “Probably No”):

There is insufficient information about incomplete outcome data to permit a judgment of high risk of bias, but there is indirect evidence which suggests incomplete outcome data was not adequately addressed, as described by the criteria for a judgment of high risk of bias.

Criteria for the judgment of HIGH risk of bias (i.e., answer: “No”):

Participants were not followed long enough to obtain outcome measurements

OR any one of the following:

- Reason for missing outcome data likely to be related to true outcome, with either imbalance in numbers or reasons for missing data across exposure groups; or
- For dichotomous outcome data, the proportion of missing outcomes compared with observed event risk enough to induce biologically relevant bias in intervention effect estimate; or
- For continuous outcome data, plausible effect size (difference in means or standardized difference in means) among missing outcomes enough to induce biologically relevant bias in observed effect size; or
- Potentially inappropriate application of imputation.

**7.** **Is the study report free from selective outcome reporting?**

Criteria for a judgment of LOW risk of bias (i.e., answer: “Yes”):

All the study’s pre-specified (primary and secondary) outcomes outlined in the protocol, methods, abstract, and/or introduction that are of interest in the review have been reported in the pre-specified way.

Criteria for the judgment of PROBABLY LOW risk of bias (i.e., answer: “Probably Yes”):

There is insufficient information about selective outcome reporting to permit a judgment of low risk of bias, but there is indirect evidence which suggests the study was free of selective reporting, as described by the criteria for a judgment of low risk of bias.

Criteria for the judgment of PROBABLY HIGH risk of bias (i.e., answer: “Probably No”):

There is insufficient information about selective outcome reporting to permit a judgment of high risk of bias, but there is indirect evidence which suggests the study was not free of selective reporting, as described by the criteria for a judgment of high risk of bias.

Criteria for the judgment of HIGH risk of bias (i.e., answer: “No”):

Any one of the following:

- Not all the study’s pre-specified primary outcomes (as outlined in the protocol, methods, abstract, and/or introduction) have been reported; or
- One or more primary outcomes is reported using measurements, analysis methods or subsets of the data (e.g. subscales) that were not pre-specified; or
- One or more reported primary outcomes were not pre-specified (unless clear justification for their reporting is provided, such as an unexpected effect); or
- One or more outcomes of interest are reported incompletely

**8. Is the study free of a financial conflict of interest as in did the study not receive any support from a company, study author, or other entity having a financial interest in any of the exposures studied?**

Criteria for a judgment of LOW risk of bias (i.e., answer: “yes”):

The study did not receive support from a company, study author, or other entity having a financial interest in the outcome of the study. Examples include the following:

- Funding source is limited to government, non-profit organizations, or academic grants funded by government, foundations and/or non-profit organizations;
- Chemicals or other treatment used in study were purchased from a supplier;
- Company affiliated staff are not mentioned in the acknowledgements section;
- Authors were not employees of a company with a financial interest in the outcome of the study;
- Company with a financial interest in the outcome of the study was not involved in the design, conduct, analysis, or reporting of the study and authors had complete access to the data;
- Study authors make a claim denying conflicts of interest;
- Study authors are unaffiliated with companies with financial interest, and there is no reason to believe a conflict of interest exists;
- All study authors are affiliated with a government agency (are prohibited from involvement in projects for which there is a conflict of interest or an appearance of conflict of interest).

Criteria for the judgment of PROBABLY LOW risk of bias (i.e., answer: “Probably Yes”):

There is insufficient information to permit a judgment of low risk of bias, but there is indirect evidence which suggests the study was free of support from a company, study author, or other entity having a financial interest in the outcome of the study, as described by the criteria for a judgment of low risk of bias.

Criteria for the judgment of PROBABLY HIGH risk of bias (i.e., answer: “Probably No”):

There is insufficient information to permit a judgment of high risk of bias, but there is indirect evidence which suggests the study was not free of support from a company, study author, or other entity having a financial interest in the outcome of the study, as described by the criteria for a judgment of high risk of bias.

Criteria for the judgment of HIGH risk of bias (i.e., answer: “No”):

The study received support from a company, study author, or other entity having a financial interest in the outcome of the study. Examples of support include:

- Research funds;
- Chemicals, equipment or testing provided at no cost;
- Writing services;
- Author/staff from study was employee or otherwise affiliated with company with financial interest;
- Company limited author access to the data;
- Company was involved in the design, conduct, analysis, or reporting of the study;
- Study authors claim a conflict of interest

**9. Does the study appear to be free from other problems that could put it at a risk of bias?**

Criteria for a judgment of LOW risk of bias (i.e., answer: “Yes”):

The study appears to be free of other sources of bias.

Criteria for the judgment of PROBABLY LOW risk of bias (i.e., answer: “Probably Yes”):

There is insufficient information to permit a judgment of low risk of bias, but there is indirect evidence which suggests the study was free of other threats to validity.

Criteria for the judgment of PROBABLY HIGH risk of bias (i.e., answer: “Probably No”):

There is insufficient information to permit a judgment of high risk of bias, but there is indirect evidence which suggests the study was not free of other threats to validity, as described by the criteria for a judgment of high risk of bias.

Criteria for the judgment of HIGH risk of bias (i.e., answer: “No”):

There is at least one important risk of bias. For example, the study:

- Had a potential source of bias related to the specific study design used; or
- Stopped early due to some data-dependent process (including a formal-stopping rule); or
- The conduct of the study is affected by interim results (e.g. recruiting additional participants from a subgroup showing greater or lesser effect); or
- Has been claimed to have been fraudulent; or
- Had some other problem (i.e., statistical assumptions were violated, sensitivity analyses show significant changes, no sensitivity analyses)

### Appendix B: Effect Size Transformation

Studies used different transformations for the exposure variable (natural log, log base 10, or log base 2, or no transformation). Because a one unit increase in the logarithmically transformed variable is equivalent to multiplying the original variable by the base of the logarithm used, the different transformations affect effect size interpretation. To homogenize the magnitude of effect observed in each study, results were recalculated as an absolute change in the dependent variable (i.e., IQ) for a relative difference of *k* = 1.5 times in the exposure variable (i.e., a 50% difference) ^37^. To estimate the absolute change in Y for a relative difference of *k* times in *X*, as well as the 95% confidence interval, for untransformed exposure we used the following formulae:

$$Absolute change in Y for a relative difference of k times in X =\left( k-1 \right)\cdot E\left[ X \right]\cdot\hat{\beta}$$

$$95\% CI= \left( k-1 \right)\cdot E\left[ X \right]\cdot\left[ \hat{\beta}\pm1.96\cdot se\left( \hat{\beta} \right) \right]$$

where *k* was set to 1.5, $E\left[ X \right]$ is the mean of the exposure measure and $\hat{\beta}$ is the regression coefficient of the exposure. To estimate the absolute change in Y for a relative difference of *k* times in *X*, as well as the 95% confidence interval, for a log transformed exposure we used the following formulae:

$$Absolute change in Y for a relative difference of k times in X= {log}_{b}\left( k \right)\cdot\hat{\beta}$$

$$95\% CI= {log}_{b}\left( k \right)\cdot\hat{\beta}\cdot\left[ \hat{\beta}\pm1.96\cdot se\left( \hat{\beta} \right) \right]$$

where *b* is the base of the log transformation.

1. [↑](#footnote-ref-1)
