# Supplementary material for: Sex difference of pre- and post-natal exposure to six developmental neurotoxicants on intellectual abilities: a systematic review and meta-analysis of human studies
Source: Environ Health. 2023 Nov 17;22:80. doi: 10.1186/s12940-023-01029-z (PMC10655280; doi:10.1186/s12940-023-01029-z)
Supplement: Supplementary file 2 — Additional file 2: Certainty of Evidence Table 1. Prenatal Exposure and General IQ in Males. Table 2. Prenatal Exposure and Nonverbal IQ in Males. Table 2. Prenatal Exposure and Nonverbal IQ in Males. Table 4. Postnatal Lead Exposure and General IQ in Males. Table 5. Prenatal Exposure and General IQ in Females. Table 6. Prenatal Exposure and Nonverbal IQ in Females. Table 7. Prenatal Exposure and Verbal IQ in Females. Table 8. Postnatal Lead Exposure and General IQ in Females. [file 12940_2023_1029_MOESM2_ESM.docx]

**Certainty of Evidence Supplemental Tables:**

**Table 1: Prenatal Exposure and General IQ in Males**

|  | **Rating** | **Rationale** | |
| --- | --- | --- | --- |
| **Initial Rate of Confidence** | Moderate | Human cohort studies | |
| **Downgrading Factors** | | |  |
| Risk of Bias | 0 | Our meta-analysis of only low risk of bias studies showed an effect of similar magnitude and direction | |
| Inconsistency | 0 | While some heterogeneity was observed, it was not significant enough to warrant downgrading. Our subgroup analysis by neurotoxicant accounted for some of the heterogeneity, and the leave-one-out meta-analysis confirmed that the effect size and direction remained consistent even when individual studies were excluded. | |
| Indirectness | 0 | All included studies utilized a validated biomarker for exposure and a standardized measure of IQ, ensuring the directness of the data. | |
| Imprecision | 0 | The overall confidence interval (CI) is narrow, suggesting a higher degree of precision in our findings. | |
| Publication Bias | -1 | Our funnel plot analysis did not reveal evidence of publication bias. However, we acknowledge that our results may be limited by the exclusion of studies that did not report sex-specific effects. In our systematic review, it is clear that there are several studies that found no significant differences between the sexes, but that didn’t report the sex-specific effects. The inclusion of this additional data could potentially alter our interpretation. | |
| **Upgrading Factors** | | |  |
| Large Magnitude of Association | 0 | The observed effect size is of relatively small magnitude | |
| Dose Response | 0 | We did not perform a dose-response meta-analysis, and the included studies did not report dose-response relationships. | |
| Residual Confounding | 0 | Confounding factors cannot be confidently ruled out, as most studies did not control for all tier 1 and tier 2 confounders that we considered important. | |
| **Overall** |  |  | |
| Quality | Moderate |  | |
| Strength | Limited Evidence of Toxicity | A negative relationship between exposure and outcome is observed where chance, bias, and confounding cannot be ruled out with reasonable confidence. Confidence in the relationship is constrained by such factors as the number, size, or quality of individual studies or inconsistency of findings across individual studies^.^ As more information becomes available, the estimated association could change, and this change may be large enough to alter the conclusion. | |

**Table 2: Prenatal Exposure and Nonverbal IQ in Males**

|  | **Rating** | **Rationale** |
| --- | --- | --- |
| **Initial Rate of Confidence** | Moderate | Human cohort studies |
| **Downgrading Factors** | | |
| Risk of Bias | 0 | Our meta-analysis of only low risk of bias studies showed an effect of similar magnitude and direction |
| Inconsistency | 0 | While some heterogeneity was observed, it was not significant enough to warrant downgrading. Our subgroup analysis by neurotoxicant accounted for some of the heterogeneity, and the leave-one-out meta-analysis confirmed that the effect size and direction remained consistent even when individual studies were excluded. |
| Indirectness | 0 | All included studies utilized a validated biomarker for exposure and a standardized measure of IQ, ensuring the directness of the data. |
| Imprecision | 0 | The overall confidence interval (CI) is narrow, suggesting a higher degree of precision in our findings. |
| Publication Bias | -1 | Our funnel plot analysis did not reveal evidence of publication bias. However, we acknowledge that our results may be limited by the exclusion of studies that did not report sex-specific effects. In our systematic review, it is clear that there are several studies that found no significant differences between the sexes, but that didn’t report the sex-specific effects. The inclusion of this additional data could potentially alter our interpretation. |
| **Upgrading Factors** | | |
| Large Magnitude of Association | 0 | The observed effect size is of relatively small magnitude |
| Dose Response | 0 | We did not perform a dose-response meta-analysis, and the included studies did not report dose-response relationships. |
| Residual Confounding | 0 | Confounding factors cannot be confidently ruled out, as most studies did not control for all tier 1 and tier 2 confounders that we considered important. |
| **Overall** |  |  |
| Quality | Moderate |  |
| Strength | Limited Evidence of Toxicity | A negative relationship between exposure and outcome is observed where chance, bias, and confounding cannot be ruled out with reasonable confidence. Confidence in the relationship is constrained by such factors as the number, size, or quality of individual studies or inconsistency of findings across individual studies^.^ As more information becomes available, the estimated association could change, and this change may be large enough to alter the conclusion. |

**Table 3: Prenatal Exposure and Verbal IQ in Males**

|  | **Rating** | **Rationale** |
| --- | --- | --- |
| **Initial Rate of Confidence** | **Moderate** | **Human cohort studies** |
| **Downgrading Factors** | | |
| Risk of Bias | -1 | The magnitude of the results differs when we only include low risk of bias studies |
| Inconsistency | 0 | Minimal heterogeneity was observed overall, and the leave-one-out meta-analysis confirmed that the effect size and direction remained relatively consistent even when individual studies were excluded |
| Indirectness | 0 | All included studies utilized a validated biomarker for exposure and a standardized measure of IQ, ensuring the directness of the data. |
| Imprecision | 0 | The overall confidence interval (CI) is narrow, suggesting a higher degree of precision in our findings. |
| Publication Bias | -2 | Our funnel plot analysis did not reveal evidence of publication bias. However, we acknowledge that our results may be limited by the exclusion of studies that did not report sex-specific effects. In our systematic review, several studies found no significant differences between the sexes but didn’t report the sex-specific effects. Limited studies also reported the sex-specific effect for verbal IQ compared to nonverbal IQ. The inclusion of this additional data could potentially alter our interpretation. |
| **Upgrading Factors** | | |
| Large Magnitude of Association | 0 | The observed effect size is of relatively small magnitude |
| Dose Response | 0 | We did not perform a dose-response meta-analysis, and the included studies did not report dose-response relationships. |
| Residual Confounding | 0 | Confounding factors cannot be confidently ruled out, as most studies did not control for all tier 1 and tier 2 confounders that we considered important. |
| **Overall** |  |  |
| Quality | Low |  |
| Strength | Inadequate evidence of toxicity | The available evidence is insufficient to assess effects of the exposure. Evidence is insufficient because of: the limited number of studies, and lower quality of individual studies. More information may allow an assessment of effects. |

**Table 4: Postnatal Lead Exposure and General IQ in Males**

|  | **Rating** | **Rationale** |
| --- | --- | --- |
| **Initial Rate of Confidence** | Moderate | Human cohort studies |
| **Downgrading Factors** | | |
| Risk of Bias | -2 | Three out of four studies were classified as probably high or high risk of bias. |
| Inconsistency | -1 | The findings are relatively inconsistent – two show null effect sizes, while two show negative effect sizes. The leave one out meta-analysis demonstrates the differences in interpretation of the magnitude of the affect and significance. |
| Indirectness | 0 | All included studies utilized a validated biomarker for exposure and a standardized measure of IQ, ensuring the directness of the data. |
| Imprecision | -1 | The studies are large; despite this, the overall confidence interval is quite large |
| Publication Bias | -1 | Our funnel plot analysis did not reveal evidence of publication bias. However, we acknowledge that our results may be limited by the exclusion of studies that did not report sex-specific effects from postnatal exposure up until age 6 |
| **Upgrading Factors** | | |
| Large Magnitude of Association | 0 | The observed effect size is of relatively small magnitude |
| Dose Response | 0 | We did not perform a dose-response meta-analysis, and the included studies did not report dose-response relationships. |
| Residual Confounding | 0 | Confounding factors cannot be confidently ruled out, as most studies did not control for all tier 1 and tier 2 confounders that we considered important. |
| **Overall** |  |  |
| Quality | Low |  |
| Strength | Inadequate Evidence of Toxicity | The available evidence is insufficient to assess the effects of the exposure. Evidence is insufficient because of the limited number or size of studies, the low quality of individual studies, and the inconsistency of findings across individual studies. More information may allow an assessment of the effects. |

**Table 5: Prenatal Exposure and General IQ in Females**

|  | **Rating** | **Rationale** |
| --- | --- | --- |
| **Initial Rate of Confidence** | Moderate | Human cohort studies |
| **Downgrading Factors** | | |
| Risk of Bias | 0 | Our meta-analysis of only low risk of bias studies showed an effect of similar magnitude and direction |
| Inconsistency | -1 | The interpretation of the findings is relatively inconsistent both within and across subgroups. However, the leave-one-out meta-analysis demonstrated that the effect size and direction remained consistent when individual studies were excluded. |
| Indirectness | 0 | All included studies utilized a validated biomarker for exposure and a standardized measure of IQ, ensuring the directness of the data. |
| Imprecision | 0 | The overall confidence interval (CI) is narrow, suggesting a higher degree of precision in our findings. |
| Publication Bias | -1 | Our funnel plot analysis did not reveal evidence of publication bias. However, we acknowledge that our results may be limited by the exclusion of studies that did not report sex-specific effects. In our systematic review, it is clear that several studies found no significant differences between the sexes, but that didn’t report the sex-specific effects. The inclusion of this additional data could potentially alter our interpretation. |
| **Upgrading Factors** | | |
| Large Magnitude of Association | 0 | The observed effect size is of relatively small magnitude |
| Dose Response | 0 | We did not perform a dose-response meta-analysis, and the included studies did not report dose-response relationships. |
| Residual Confounding | 0 | Confounding factors cannot be confidently ruled out, as most studies did not control for all tier 1 and tier 2 confounders that we considered important. |
| **Overall** |  |  |
| Quality | Low |  |
| Strength | Inadequate Evidence of Toxicity | The available evidence is insufficient to assess the effects of the exposure. Evidence is insufficient because of the inconsistency of findings across subgroups and individual studies, it is unclear whether the direction of the effect size is positive, null, or negative |

**Table 6: Prenatal Exposure and Nonverbal IQ in Females**

|  | **Rating** | **Rationale** |
| --- | --- | --- |
| **Initial Rate of Confidence** | Moderate | Human cohort studies |
| **Downgrading Factors** | | |
| Risk of Bias | 0 | Our meta-analysis of only low risk of bias studies showed an effect of similar magnitude and direction |
| Inconsistency | 0 | Very minimal heterogeneity, and the leave-one-out meta-analysis confirmed that the effect size and direction remained consistent even when individual studies were excluded. |
| Indirectness | 0 | All included studies utilized a validated biomarker for exposure and a standardized measure of IQ, ensuring the directness of the data. |
| Imprecision | 0 | The overall confidence interval (CI) is narrow, suggesting a higher degree of precision in our findings. |
| Publication Bias | -1 | Our funnel plot analysis did not reveal evidence of publication bias. However, we acknowledge that our results may be limited by the exclusion of studies that did not report sex-specific effects. In our systematic review, it is clear that several studies found no significant differences between the sexes, but that didn’t report the sex-specific effects. The inclusion of this additional data could potentially alter our interpretation. |
| **Upgrading Factors** | | |
| Large Magnitude of Association | 0 | The observed effect size is of relatively small magnitude |
| Dose Response | 0 | We did not perform a dose-response meta-analysis, and the included studies did not report dose-response relationships. |
| Residual Confounding | 0 | Confounding factors cannot be confidently ruled out, as most studies did not control for all tier 1 and tier 2 confounders that we considered important. |
| **Overall** |  |  |
| Quality | Moderate |  |
| Strength | Insufficient Evidence of Toxicity | A null relationship is observed between exposure and outcome, and chance, bias and confounding can be ruled out with reasonable confidence. The available evidence includes consistent results from more than one well-designed, well-conducted study at the full range of exposure levels that humans are known to encounter, and the conclusion is unlikely to be strongly affected by the results of future studies. The conclusion is limited to the age at exposure and/or other conditions and levels of exposure studied. |

**Table 7: Prenatal Exposure and Verbal IQ in Females**

|  | **Rating** | **Rationale** |
| --- | --- | --- |
| **Initial Rate of Confidence** | Moderate | Human cohort studies |
| **Downgrading Factors** | | |
| Risk of Bias | 0 | Our meta-analysis of only low risk of bias studies showed an effect of similar magnitude and direction |
| Inconsistency | 0 | While some heterogeneity was observed, it was not significant enough to warrant downgrading and the leave-one-out meta-analysis confirmed that the effect size and direction remained consistent even when individual studies were excluded. |
| Indirectness | 0 | All included studies utilized a validated biomarker for exposure and a standardized measure of IQ, ensuring the directness of the data. |
| Imprecision | 0 | The overall confidence interval (CI) is narrow, suggesting a higher degree of precision in our findings. |
| Publication Bias | -2 | Our funnel plot analysis did not reveal evidence of publication bias. However, we acknowledge that our results may be limited by the exclusion of studies that did not report sex-specific effects. In our systematic review, it is clear that several studies found no significant differences between the sexes, but that didn’t report the sex-specific effects. There are also limited studies that reported the sex-specific effect for the verbal IQ compared to nonverbal IQ. The inclusion of this additional data could potentially alter our interpretation. |
| **Upgrading Factors** | | |
| Large Magnitude of Association | 0 | The observed effect size is of relatively small magnitude |
| Dose Response | 0 | We did not perform a dose-response meta-analysis, and the included studies did not report dose-response relationships. |
| Residual Confounding | 0 | Confounding factors cannot be confidently ruled out, as most studies did not control for all tier 1 and tier 2 confounders that we considered important. |
| **Overall** |  |  |
| Quality | Moderate |  |
| Strength | Inadequate Evidence of Toxicity | The available evidence is insufficient to assess effects of the exposure. Evidence is insufficient because of: the limited number of studies. More information may allow an assessment of effects. |

**Table 8: Postnatal Lead Exposure and General IQ in Females**

|  | **Rating** | **Rationale** |
| --- | --- | --- |
| **Initial Rate of Confidence** | Moderate | Human cohort studies |
| **Downgrading Factors** | | |
| Risk of Bias | -2 | Three out of four studies were classified as probably high or high risk of bias. |
| Inconsistency | -1 | The findings are relatively inconsistent – two show null effect sizes, while two show negative effect sizes. The leave one out meta-analysis demonstrates the differences in interpretation of the magnitude of the effect. |
| Indirectness | 0 | All included studies utilized a validated biomarker for exposure and a standardized measure of IQ, ensuring the directness of the data. |
| Imprecision | -1 | The studies are large; despite this, the overall confidence interval is quite large |
| Publication Bias | -2 | Our funnel plot analysis revealed evidence of publication bias. We also acknowledge that our results may be limited by the exclusion of studies that did not report sex-specific effects from postnatal exposure up until age 6 |
| **Upgrading Factors** | | |
| Large Magnitude of Association | 0 | The observed effect size is of relatively small magnitude |
| Dose Response | 0 | We did not perform a dose-response meta-analysis, and the included studies did not report dose-response relationships. |
| Residual Confounding | 0 | Confounding factors cannot be confidently ruled out, as most studies did not control for all tier 1 and tier 2 confounders that we considered important. |
| **Overall** |  |  |
| Quality | Low |  |
| Strength | Inadequate evidence of toxicity | The available evidence is insufficient to assess effects of the exposure. Evidence is insufficient because of: the limited number or size of studies, low quality of individual studies, and the inconsistency of findings across individual studies. More information may allow an assessment of effects. |
