# Supplementary material for: Sex difference of pre- and post-natal exposure to six developmental neurotoxicants on intellectual abilities: a systematic review and meta-analysis of human studies
Source: Environ Health. 2023 Nov 17;22:80. doi: 10.1186/s12940-023-01029-z (PMC10655280; doi:10.1186/s12940-023-01029-z)
Supplement: Supplementary file 3 — Additional file 3. Rationale for Risk of Bias Determinations for Each Study Included in the Meta-Analysis. [file 12940_2023_1029_MOESM3_ESM.docx]

**Supplementary Tables:**

**Rationale for Risk of Bias Determinations for Each Study Included in the Meta-Analysis**

**Table 1: Azar, 2021**

| Domain | Risk of Bias Rating | Explanation |
| --- | --- | --- |
| Selection Bias | Low risk of bias | The descriptions of the source population, inclusion/exclusion criteria, recruitment and enrollment procedures, participation and follow-up rates were sufficiently detailed, and adequate data were supplied on the distribution of relevant study sample and population characteristics to support the assertion that risk of selection effects was minimal (table S1). |
| Blinding | Probably low risk of bias | The reviewers determine the outcome measures as well as the exposure measures are not likely to be influenced by lack of blinding. The exposure was measured by a separate entity (INSPQ) and the outcome was obtained from home visits. |
| Exposure Assessment | Probably low risk of bias | PBDEs were measured in blood samples and standardized for total lipid concentrations. There may be insufficient information about the QA/QC methods to permit a judgement of low risk of bias. However, indirect evidence suggests that methods were robust, as described by the criteria for a judgement of low risk of bias (see Fisher et al., 2016). |
| Outcome Assessment | Probably low risk of bias | Outcomes were assessed and defined consistently across all study participants, using valid and reliable measures of intelligence (WPPSI). Although, there is no mention of QA/QC |
| Confounding | Probably low risk of bias | The study appropriately assessed and considered/accounted for most but not all the important confounders (Tier I) and most of the other potentially important confounders relevant (Tier II). Confounders considered include maternal age, pre-pregnancy BMI, birth weight, gestational age, race/ethnicity, maternal birth country, maternal education, household annual income, marital status, parity, self-reported smoking, self-reported alcohol consumption, child sex, and study site. |
| Incomplete Outcome Data | Low risk of bias | The characteristics of the mother–child pairs included were comparable to those of the initial MIREC cohort, within the six study sites involved in the neuro-developmental follow-up study. Therefore, the reasons for missing outcome data are unlikely to be related to the true outcome. |
| Selective Outcome Reporting | Low risk of bias | All of the study’s pre-specified (primary and secondary) outcomes outlined in the protocol, methods, abstract, and/or introduction that are of interest have been reported in the pre-specified way. |
| Conflict of Interest | Low risk of bias | The authors declare that they have no known competing financial interests or personal relationships |
| Other | Low risk of bias | The study appears to be free of other sources of bias. |

**Table 2: Bouchard, 2011**

| Domain | Risk of Bias Rating | Explanation |
| --- | --- | --- |
| Selection Bias | Low risk of bias | There is sufficient information regarding recruitment, inclusion/exclusion, and follow-up. Families included in the analysis did not differ significantly from the original full cohort on most attributes; however, mothers of children included were slightly older and children were breast-fed for longer than those from the initial cohort. |
| Blinding | Probably low risk of bias | The outcome measures, as well as the exposure measures, are not likely to be influenced by lack of blinding due to the exposure being measured by a separate entity than the outcome. |
| Exposure Assessment | Probably low risk of bias | There may be insufficient information about the exposure assessment methods to permit a judgment of low risk of bias. However, there is indirect evidence, which suggests that methods were robust, as described by the criteria for a judgment of low risk of bias. See Bradman et al. 2005 for details of urine collection, analysis, detection frequencies, and quality control procedures. |
| Outcome Assessment | Low risk of bias | Outcomes were assessed and defined consistently across all study participants, using valid and reliable measures of intelligence (WISC-IV) |
| Confounding | Probably low risk of bias | The study appropriately assessed and considered/accounted for most of the important confounders (Tier I), and some of the other potentially important confounders (Tier II). Confounders included maternal IQ, education, HOME score, language, breastfeeding, maternal age, birth order, poverty, marital status, children's age at testing, and maternal levels of PBDEs, PCBs, DDE, DDT, and lead. |
| Incomplete Outcome Data | Low risk of bias | As supported by the following text:  "For subtests in which a ceiling was not achieved (< 10%), missing values were imputed based on scores obtained by other children with similar score patterns." |
| Selective Outcome Reporting | Low risk of bias | All of the study’s pre‐specified outcomes outlined in the methods that are of interest have been reported in the pre‐specified way. |
| Conflict of Interest | High risk of bias | B.E. has consulted in a case of pesticide exposure. The other authors declare they have no competing financial interests. |
| Other | Low risk of bias | The study appears to be free of other sources of bias. |

**Table 3: Desrochers-Couture, 2018**

| Domain | Risk of Bias Rating | Explanation |
| --- | --- | --- |
| Selection Bias | Probably low risk of bias | The descriptions of the source population, inclusion/exclusion criteria, recruitment process and enrollment procedures, participation, as well as follow-up rates were sufficiently detailed. Adequate data were supplied on the distribution of relevant study samples. Indirect data from Arbuckle, 2013 were supplied on the distribution of relevant population characteristics. |
| Blinding | Low risk of bias | As supported by the following text: “A trained research assistant unaware of children's exposure to environmental contaminants administered the WPPSI" |
| Exposure Assessment | Probably low risk of bias | Lead was measured in blood samples using a valid and accurate method (inductively coupled plasma mass spectrometry) at an institution with an accreditation of the Standards Council of Canada. LOD reported and concentrations <LOD were replaced by the value LOD/√2. Blood samples were measured at 4 different time points (1st and 3rd trimester, birth, and at age 3-4). Child blood was measured using venous samples. There is no additional information on standards recovery, measures of repeatability, or the investigation of blanks contamination |
| Outcome Assessment | Low risk of bias | Intelligence was assessed using the WPPSI-III using the age-standardized WPPSI-III Canadian norms. A trained research assistant administered the test. |
| Confounding | Low risk of bias | The study accounted for all important confounders (Tier I) and most of the potentially important confounders (Tier II). They also took into consideration birth weight and gestational age and the study inclusion criteria accounted for drug use and fetal anomalies. Confounders considered include marital status, household income, education, parity, ethnicity, current smoking and alcohol consumption, total mercury concentrations, depression, the Parenting Stress Index, HOME score, study site, child age and sex |
| Incomplete Outcome Data | Low risk of bias | Regression models were tested with a full information maximum likelihood (FIML) estimator, enabling them to keep all the available data instead of restricting the analyses to complete cases only. |
| Selective Outcome Reporting | Low risk of bias | All of the study’s pre‐specified outcomes outlined in the methods that are of interest have been reported in the pre‐specified way. |
| Conflict of Interest | Low risk of bias | The authors declare that they have no actual or potential competing financial or non-financial interests. |
| Other | Low risk of bias | The study appears to be free of other biases. |

**Table 4: Dietrich, 1993**

| Domain | Risk of Bias Rating | Explanation |
| --- | --- | --- |
| Selection Bias | Probably low risk of bias | There is indirect evidence which suggests the inclusion/exclusion criteria, recruitment process and enrollment procedures, as well as participation and follow-up rates were sufficiently detailed (Dietrich et al., 1991). Subjects retained in the sample did not differ from those lost to follow-up in terms of perinatal medical status, maternal age, maternal IQ, or on measures of the quality of caretaking in the home environment. |
| Blinding | Probably low risk of bias | There is insufficient information about blinding to permit a judgment of low risk of bias, but there is indirect evidence which suggests the study was adequately blinded (Dietrich et al., 1991). |
| Exposure Assessment | Probably low risk of bias | Lead was measured in whole blood. There may be insufficient information about the methods to permit a judgement of low risk of bias. However, indirect evidence suggests the methods were robust (Dietrich et al., 1987; Dietrich et al., 1991; and Roda et al., 1988). |
| Outcome Assessment | Low risk of bias | Outcomes were assessed and defined consistently across all study participants, using valid and reliable measures of intelligence (I.e., WISC-R) |
| Confounding | High risk of bias | The study appropriately assessed and considered some important confounders (Tier I) but did not account for multiple other potentially important confounders (Tier II). Confounders that were considered include birth weight, birth length, neonatal head circumference, gestational age by physical exam, gestational age by dates, apgar at one minute, apgar at five minutes, Obstetrical Complications Scale Score, Postnatal Complications Scale Score, cigarette consumption during pregnancy, alcohol consumption, marijuana consumption, narcotics use, number of previous abortions, number of previous stillbirths, gravidity, parity, hemoglobin, Social Class Score, HOME Score, maternal intelligence, highest grade attained by primary caregiver, family on public assistance, number of adults at home, number of children at home, child attends preschool, child race |
| Incomplete Outcome Data | Probably high risk of bias | Four subjects were excluded from analyses for medical conditions other than lead poisoning (Visual handicap, n = 1, Tourette's Syndrome, n = 1, Fetal Alcohol Syndrome, n = 1, and Sickle Cell Anemia, n = 1). When compared to children who were not evaluated, subjects in the present sample had slightly higher blood lead (PbB) concentrations in the first year of life. |
| Selective Outcome Reporting | Low risk of bias | All of the study’s pre-specified (primary and secondary) outcomes outlined in the protocol, methods, abstract, and/or introduction that are of interest have been reported in the pre-specified way. |
| Conflict of Interest | Low risk of bias | This research has been supported by a Program Project Grant from the National Institute of Environmental Health Sciences (POLES01566). Paul B. Hammond and Robert L. Bornschein were directors of the overall program |
| Other | Low risk of bias | The study appears to be free of other sources of bias. |

**Table 5: Eskenazi, 2013**

| Domain | Risk of Bias Rating | Explanation |
| --- | --- | --- |
| Selection Bias | Probably low risk of bias | There are sufficient details on the recruitment process, inclusion/exclusion, and subsample. Compared with children in the cohort who were not followed, children included in the present analyses were more likely to be female and born full term, with mothers who were older, breastfed longer, and were less likely to smoke or drink during pregnancy. They did not differ according to other sociodemographic characteristics or by prenatal PBDE levels |
| Blinding | Probably low risk of bias | The review authors judge that the outcome measures, as well as the exposure measures, are not likely to be influenced by lack of blinding. Exposure was analyzed by a separate entity (CDC) |
| Exposure Assessment | Low risk of bias | PBDEs measured in serum and lipid adjusted; appropriate QA/QC methods are described. Therefore, reviewers judge that there is a low risk of exposure misclassification, i.e., there is high confidence in the accuracy of the exposure assessment methods, such as methods that have been tested for validity and reliability in measuring the targeted exposure |
| Outcome Assessment | Low risk of bias | Outcomes were assessed and defined consistently across all study participants, using valid and reliable measures of intelligence (WPPSI-III & WISC-IV) |
| Confounding | Probably low risk of bias | The study appropriately assessed and considered most important confounders (Tier I) and all other potentially important confounders relevant (Tier II). Confounders considered include: maternal age, maternal education, years in the US, marital status, work outside the home, use of alcohol and tobacco during pregnancy, depression, parity, PPVT/TVIP score, housing density, household poverty, pregnancy exposure to smoke, number of children, father's presence, HOME score, child care attendance, psychometrician, location, language of assessment, child sex, birth weight, preterm delivery status, maternal thyroid hormones, DAPs, lead, and PCBs, |
| Incomplete Outcome Data | Low risk of bias | There is some but not substantial amounts of missing data. |
| Selective Outcome Reporting | Low risk of bias | All of the study’s pre-specified (primary and secondary) outcomes outlined in the protocol, methods, abstract, and/or introduction that are of interest have been reported in the pre-specified way. |
| Conflict of Interest | Low risk of bias | The authors declare they have no competing financial interests |
| Other | Low risk of bias | The study appears to be free of other sources of bias. |

**Table 6: Gascon, 2015**

| Domain | Risk of Bias Rating | Explanation |
| --- | --- | --- |
| Selection Bias | Probably low risk of bias | There is insufficient information about participant selection to permit a judgment of low risk of bias, but there is indirect evidence which suggests that inclusion/exclusion criteria, recruitment and enrollment procedures, as well as participation and follow-up rates, were consistent across groups (see Guxens et al., 2012). The included participants were younger and of lower education than excluded birth cohort participants. |
| Blinding | Probably low risk of bias | The review authors judge that the outcome measures as well as the exposure measures are not likely to be influenced by lack of blinding. The exposure was measured by a separate entity as the outcome. i.e., hospital versus health care centre |
| Exposure Assessment | Probably low risk of bias | Urine samples were adjusted for creatinine instead of specific gravity. There may be insufficient information about the exposure assessment methods to permit a judgement of low risk of bias. However, there is indirect evidence, which suggests that methods were robust, as described by the criteria for a judgement of low risk of bias. Further details about QA/QC in Valvi et al., 2015 |
| Outcome Assessment | Low risk of bias | Outcomes were assessed and defined consistently across all study participants, using valid and reliable measures of intelligence (MSCA) |
| Confounding | Probably high risk of bias | The study evaluated some but not all the important confounders (Tier I) and some but not all the other potentially important confounders relevant (Tier II). Confounders considered include: maternal age, social class, education and country of origin, maternal smoking during pregnancy, number of older siblings, daycare attendance during the 1st year of life, duration of exclusive breastfeeding and pre-pregnancy maternal BMI. |
| Incomplete Outcome Data | Low risk of bias | Missing data have been imputed using appropriate methods |
| Selective Outcome Reporting | Probably low risk of bias | It is unclear whether there were any significant findings with interactions by sex, as the in-text wording states "we did not observe consistent sex specific associations between the different phthalates analyzed and cognitive or psychomotor development" |
| Conflict of Interest | Low risk of bias | Study authors make a claim denying conflicts of interest; |
| Other | Low risk of bias | The study appears to be free of other sources of bias. |

**Table 7: Guo, 2020**

| Domain | Risk of Bias Rating | Explanation |
| --- | --- | --- |
| Selection Bias | Probably low risk of bias | There is insufficient information about participant selection to permit a judgment of low risk of bias, but there is indirect evidence that suggests that inclusion/exclusion criteria, recruitment and enrollment procedures, as well as participation and follow-up rates were consistent across groups as described by the criteria for a judgment of low risk of bias (see Liu et al., 2016; Qi et al., 2012). Compared to the total participants and the excluded subjects, the participated women in the current study tended to have slightly lower education and higher household income during pregnancy |
| Blinding | Probably low risk of bias | The outcome measures as well as the exposure measures are not likely to be influenced by lack of blinding. The exposure was collected during delivery day at the hospital and the outcome was assessed at a separate time by pediatricians. |
| Exposure Assessment | Probably high risk of bias | There is insufficient information about the exposure assessment methods to permit a judgment of high risk of bias; however, there is indirect evidence which suggests that methods were not robust, as described by the criteria for a judgment of high risk of bias. Heavy metals were measured in urine spot samples as opposed to in blood. Assessment methods were described in other studies but information about lead and mercury were less clear. (See Guo et al., 2019; Guo et al., 2017; Qi et al., 2012; and Zhou et al., 2019) |
| Outcome Assessment | Low risk of bias | Outcomes were assessed and defined consistently across all study participants, using valid and reliable measures of intelligence (C-WISC) |
| Confounding | Probably high risk of bias | The study included some but not all the important confounders (Tier I), and some but not all the other potentially important confounders relevant (Tier II). Confounders accounted for included: maternal age, education, family income, smoking during pregnancy, breastfeeding duration, sex of the child, physicians, marital status, and creatinine |
| Incomplete Outcome Data | Low risk of bias | Missing data have been imputed using appropriate methods. |
| Selective Outcome Reporting | Low risk of bias | All of the study’s pre-specified (primary and secondary) outcomes outlined in the protocol, methods, abstract, and/or introduction that are of interest have been reported in the pre-specified way. |
| Conflict of Interest | Low risk of bias | The authors declare they have no competing financial interests |
| Other | Low risk of bias | The study appears to be free of other sources of bias. |

**Table 8: Huang, 2012**

| Domain | Risk of Bias Rating | Explanation |
| --- | --- | --- |
| Selection Bias | Probably high risk of bias | The descriptions of the source population, inclusion/exclusion criteria, recruitment and enrollment procedures, as well as participation, and follow-up rates were sufficiently detailed. Children with outcome data at 2-3 years of age had slightly lower maternal and cord blood lead than those without outcome data (not significant at p<.05, as it would produce biased results toward the null). The authors did not discuss these differences in children followed-up at age 5-6 and 8-9. |
| Blinding | Probably low risk of bias | The review authors judge that the outcome and exposure measures, are not likely to be influenced by lack of blinding. Lead was measured within a hospital setting, and intelligence was assessed by trained psychologists elsewhere. |
| Exposure Assessment | Probably low risk of bias | Lead was measured using whole blood samples. Further, indirect evidence suggests that appropriate QA/QC methods were robust, as described by Wang et al., 2002. |
| Outcome Assessment | Low risk of bias | Outcomes were assessed and defined consistently across all study participants, using valid and reliable measures of intelligence (i.e., BSID-II, and the Chinese version of the WPPSI-R and WISC-III). |
| Confounding | Probably high risk of bias | The study evaluated some but not all the important confounders (Tier I) and most but not all the other potentially important confounders (Tier II). The covariates included HOME scores, maternal education, maternal age, birth weight, pesticide use, pre-pregnancy smoking, and post-pregnancy alcohol use. |
| Incomplete Outcome Data | Probably low risk of bias | There is incomplete outcome data due to divorce, migration, accident, personal reasons, lack of health status of newborns, lack of blood sample, or incomplete BSID-II; these reasons are unlikely to be related to the true outcome. |
| Selective Outcome Reporting | Low risk of bias | All of the study’s pre-specified (primary and secondary) outcomes outlined in the protocol, methods, abstract, and/or introduction that are of interest have been reported in the pre-specified way. |
| Conflict of Interest | Low risk of bias | Study authors make a claim denying conflicts of interest |
| Other | Low risk of bias | The study appears to be free of other sources of bias |

**Table 9: Hyland, 2019**

| Domain | Risk of Bias Rating | Explanation |
| --- | --- | --- |
| Selection Bias | Low risk of bias | The descriptions of the source population, inclusion/exclusion criteria, recruitment and enrollment procedures, as well as participation and follow-up rates were sufficiently detailed, and adequate data were supplied on the distribution of relevant study samples and population characteristics to support the assertion that risk of selection effects was minimal. |
| Blinding | Probably low risk of bias | The review authors judge that the outcome and exposure measures are not likely to be influenced by a lack of blinding. Exposure was measured by a separate entity to the outcome. |
| Exposure Assessment | Low risk of bias | There is high confidence in the accuracy of the exposure assessment methods, such as methods tested for validity and reliability in measuring the targeted exposure; Phthalates were measured in spot urine samples adjusted for urinary dilution through specific gravity. Appropriate QA/QC for methods are described and are satisfactory. |
| Outcome Assessment | Low risk of bias | Outcomes were assessed and defined consistently across all study participants, using valid and reliable measures of intelligence (WISC-IV) |
| Confounding | Probably low risk of bias | The study appropriately assessed and considered or accounted for most but not all the important confounders (Tier I), and most but not all other potentially important confounders (Tier II). Confounders considered include maternal parity, prenatal diet, smoking, alcohol and illicit drug use, mode of delivery, duration of breastfeeding, parental marital status, maternal education, maternal employment, household income, depression, HOME scores, OPPS, OCPS and PBDEs, and Mn |
| Incomplete Outcome Data | Low risk of bias | Missing data have been imputed using appropriate methods. |
| Selective Outcome Reporting | Low risk of bias | All of the study’s pre-specified (primary and secondary) outcomes outlined in the protocol, methods, abstract, and/or introduction that are of interest have been reported in the pre-specified way. |
| Conflict of Interest | Low risk of bias | The authors declare they have no competing financial interests. |
| Other | Probably low risk of bias | The authors conducted a large number of analyses with multiple comparisons, which could produce spurious associations by chance alone. Nonetheless, patterns were carefully reported in the results rather than highlighting isolated findings. |

**Table 10: Julvez, 2013**

| Domain | Risk of Bias Rating | Explanation |
| --- | --- | --- |
| Selection Bias | Probably low risk of bias | There is detailed information about the ALSPAC cohort in Fraser 2013. Among children with available outcome data, participants had higher mean IQ scores than non-participants (107 vs 105) |
| Blinding | Probably low risk of bias | The review authors judge that the outcome and exposure measures, are not likely to be influenced by lack of blinding |
| Exposure Assessment | Low risk of bias | QA/QC explanations are clear. Hg was measured using cord tissue samples; authors note cord blood may show greater precision as risk indicator for MeHg neurotoxicity than material hair samples (Grandjean et al., 2011; Grandjean et al., 2005) |
| Outcome Assessment | Probably low risk of bias | Outcomes were assessed and defined consistently across all study participants, using valid and reliable measures of intelligence (i.e., short form of WISC-III). However, QA/QC are not described. |
| Confounding | Probably low risk of bias | The study assessed and considered most of the important confounders (Tier I) but did not account for other potentially important confounders (Tier II). Confounders considered include sex, age, examiner, fatty acid intake from fish consumption, maternal age, parity, house ownership status, parental education, social class, HOME scores, and stressful life events |
| Incomplete Outcome Data | Probably low risk of bias | The reasons for missing IQ data are unclear, but given that it is a relatively small amount of missing data, it is unlikely to have a large effect |
| Selective Outcome Reporting | Low risk of bias | All of the study’s pre-specified (primary and secondary) outcomes outlined in the protocol, methods, abstract, and/or introduction that are of interest have been reported in the pre-specified way. |
| Conflict of Interest | Low risk of bias | Study authors make a claim denying conflicts of interest |
| Other | Low risk of bias | The study appears to be free of other sources of bias. |

**Table 11: Jusko, 2019**

| Domain | Risk of Bias Rating | Explanation |
| --- | --- | --- |
| Selection Bias | Probably low risk of bias | The descriptions of the source population, inclusion/exclusion criteria, recruitment and enrollment procedures, as well as participation and follow-up rates were sufficiently detailed, and adequate data were supplied on the distribution of relevant study sample and population characteristics. Compared with all women in the Generation R Study, the women in the analysis were more likely to be older, nulliparous, Dutch, highly educated, married, and to occasionally consume alcoholic beverages during pregnancy. Author’s note selection bias is unlikely. |
| Blinding | Probably low risk of bias | The review authors judge that the outcome and exposure measures are not likely to be influenced by lack of blinding. Exposure was measured by a separate entity in Quebec, whereas IQ was measured at the research center in the Netherlands. |
| Exposure Assessment | Low risk of bias | The reviewers judge that there is a low risk of exposure misclassification. There is high confidence in the accuracy of the exposure assessment methods, such as methods that have been tested for validity and reliability in measuring the targeted exposure. OP Pesticides were measured in urine and adjusted for urinary dilution. Appropriate QA/QC for methods are described and are satisfactory |
| Outcome Assessment | Probably low risk of bias | Outcomes were assessed and defined consistently across all study participants, using valid and reliable measures of intelligence (SON-R IQ). No QA/QC are described. |
| Confounding | Low risk of bias | The study appropriately assessed and considered all important confounders (Tier I), and most but not necessarily all other potentially important confounders (Tier II). Confounders considered include, maternal age, ethnicity, education, income, marital status, alcohol consumption, IQ, BMI, height, parity, smoking, sex, and HOME scores. |
| Incomplete Outcome Data | Low risk of bias | Reasons for exclusion are described in a clear and concise manner. |
| Selective Outcome Reporting | Low risk of bias | All of the study’s pre-specified (primary and secondary) outcomes outlined in the protocol, methods, abstract, and/or introduction that are of interest have been reported in the pre-specified way. |
| Conflict of Interest | Low risk of bias | All authors declare they have no competing financial interests. |
| Other | Low risk of bias | The study appears to be free of other sources of bias. |

**Table 12: Li, 2019**

| Domain | Risk of Bias Rating | Explanation |
| --- | --- | --- |
| Selection Bias | Probably low risk of bias | There is insufficient information about participant selection to permit a judgment of low risk of bias, but there is indirect evidence which suggests that the inclusion/exclusion criteria, recruitment and enrollment procedures, as well as participation and follow-up rates were consistent across groups as described by the criteria for a judgment of low risk of bias (See Braun et al., 2017). |
| Blinding | Probably low risk of bias | There is insufficient information about blinding to permit a judgment of low risk of bias, but there is indirect evidence which suggests the study was adequately blinded, as described by the criteria for a judgment of low risk of bias (see chen et al., 2014). |
| Exposure Assessment | Probably low risk of bias | There may be insufficient information about the exposure assessment methods to permit a judgement of low risk of bias but there is indirect evidence, which suggests that methods were robust, as described by the criteria for a judgement of low risk of bias (Silva et al., 2007). |
| Outcome Assessment | Low risk of bias | Outcomes were assessed and defined consistently across all study participants, using valid and reliable measures of intelligence (WPPSi-III, WISC-IV) |
| Confounding | Probably low risk of bias | The study appropriately assessed and considered all important confounders (Tier I), and some but not all other potentially important confounders (Tier II). Confounders considered include: maternal age, education, income, race, sex, pregnancy hypertension, pre pregnancy BMI, maternal IQ, smoke, cotinine, HOME scores, creatinine and visit. |
| Incomplete Outcome Data | Probably high risk of bias | Information on incomplete outcome data is unclear. |
| Selective Outcome Reporting | Low risk of bias | All of the study’s pre-specified (primary and secondary) outcomes outlined in the protocol, methods, abstract, and/or introduction that are of interest have been reported in the pre-specified way. |
| Conflict of Interest | Low risk of bias | Study authors make a claim denying conflicts of interest |
| Other | Low risk of bias | The study appears to be free of other sources of bias. |

**Table 13: Llop, 2017**

| Domain | Risk of Bias Rating | Explanation |
| --- | --- | --- |
| Selection Bias | Low risk of bias | The descriptions of the source population, inclusion/exclusion criteria, recruitment and enrollment procedures, as well as participation and follow-up rates were sufficiently detailed, and adequate data were supplied on the distribution of relevant study sample and population characteristics to support the assertion that risk of selection effects was minimal. |
| Blinding | Probably low risk of bias | There is insufficient information about blinding to permit a judgment of low risk of bias, but there is indirect evidence which suggests the study was adequately blinded, as described by the criteria for a judgment of low risk of bias (see Casas et al., 2015). |
| Exposure Assessment | Low risk of bias | The reviewers judge that there is low risk of exposure misclassification, i.e., there is high confidence in the accuracy of the exposure assessment methods, such as methods that have been tested for validity and reliability in measuring the targeted exposure. |
| Outcome Assessment | Low risk of bias | Outcomes were assessed and defined consistently across all study participants, using valid and reliable measures of intelligence (i.e., MSCA). Tests were administered by six psychologists or neuropsychologists and adapted to the Spanish population. |
| Confounding | Probably high risk of bias | The study evaluated some but not all the important confounders (Tier I) and some but not all the other potentially important confounders (Tier-II). Confounders considered include maternal age, education level, country of birth, birth order, BMI, working status during pregnancy, the season of birth, parental social class, fish intake, PUFA concentrations, gestational age, sex, duration of breastfeeding, maternal and paternal working status, smoking habit, main care provider, and maternal IQ |
| Incomplete Outcome Data | Low risk of bias | “Thirty-four percent of children who were followed until 4–5 years did not take part in the study, the main cause being the unavailability of cord blood samples in which to analyze mercury concentrations. Differences between the included and excluded population were evaluated and a marginal difference according to the maternal smoking habit was observed (p = 0.07): the percentage of smokers at the time of neuropsychological testing was slightly lower in the study population (26%) compared with the excluded women (30%).” |
| Selective Outcome Reporting | Low risk of bias | All of the study’s pre‐specified outcomes outlined in the methods that are of interest have been reported in a pre‐specified way. |
| Conflict of Interest | Low risk of bias | Study authors make a claim denying conflicts of interest |
| Other | Low risk of bias | The study appears to be free of other sources of bias. |

**Table 14: McMichael, 1992**

| Domain | Risk of Bias Rating | Explanation |
| --- | --- | --- |
| Selection Bias | Probably high risk of bias | There is insufficient information about the inclusion/exclusion criteria. Although, recruitment and enrollment procedures as well as participation and follow‐up rates are detailed. There is indirect information about differences between children remaining in the cohort at age 7 and those lost to follow-up. However, there is no information regarding children remaining in the cohort at age 4. There is also no mention of differential lead exposure. |
| Blinding | Low risk of bias | As supported by the following text: “A research psychologist who was unaware of the child's blood lead status conducted all testing sessions in a clinic setting. Although the psychologist had also assessed the child's abilities (using BSID) at the age of 2 years, he was not aware of that earlier result when the MSCA was conducted.” |
| Exposure Assessment | Probably low risk of bias | There is insufficient information about the exposure assessment methods to permit a judgment of low risk of bias, but there is indirect evidence that suggests that the methods were robust (see McMichael et al, 1985). |
| Outcome Assessment | Low risk of bias | Intelligence was assessed by a research psychologist and was defined consistently across all study participants, using valid and reliable measures (MSCA). |
| Confounding | Probably high risk of bias | The study evaluated some but not all important confounders (Tier I) and did not account for or evaluate other potentially important confounders relevant (Tier II). Confounders considered include gender, parental smoking, socioeconomic status, quality of the home environment, birth weight, and method of feeding. |
| Incomplete Outcome Data | Probably high risk of bias | There is insufficient information about incomplete outcome data to permit a judgment of high risk of bias. The majority (approx. 80%) of children lost to follow-up at age 4 were in the families that left the Port Pirie District. A small number of families (about 20%) simply discontinued their participation. It is unclear how the number of families that discontinued their participation is related to the outcome. |
| Selective Outcome Reporting | Low risk of bias | All of the study’s pre‐specified outcomes outlined in the methods that are of interest have been reported in the pre‐specified way. |
| Conflict of Interest | Low risk of bias | Funding source is limited to government and academic grants. |
| Other | Low risk of bias | The study appears to be free of other sources of bias. |

**Table 15: Ntantu Nkinsa, 2020**

| Domain | Risk of Bias Rating | Explanation |
| --- | --- | --- |
| Selection Bias | Low risk of bias | The descriptions of the source population, inclusion/exclusion criteria, recruitment and enrollment procedures, as well as participation and follow-up rates were sufficiently detailed, and adequate data were supplied on the distribution of relevant study sample and population characteristics to support the assertion that risk of selection effects was minimal. |
| Blinding | Low risk of bias | As supported by the following text: “Testing was performed…by trained research personnel with no knowledge of the participants’ level of exposure to environmental contaminants." |
| Exposure Assessment | Probably low risk of bias | There may be insufficient information about the exposure assessment methods to permit a judgment of low risk of bias but there is indirect evidence, which suggests that methods were robust, as described by the criteria for a judgment of low risk of bias. Further details on laboratory methods and descriptive statistics on in-dividual DAP metabolites have been previously published (Sokoloff et al., 2016). While DAP was measured in urine and adjusted for SG, there are not multiple measurements. |
| Outcome Assessment | Low risk of bias | Outcomes were assessed and defined consistently across all study participants, using valid and reliable measures of intelligence (WPPSI-III0. |
| Confounding | Low risk of bias | The study appropriately assessed and considered all important confounders (Tier I), and most but not necessarily all other potentially important confounders (Tier II). Confounders considered include: maternal age, education, parity, race, income, marital status, employment, pre-BMI, smoking, alcohol consumption, HOME scores, and study site |
| Incomplete Outcome Data | Probably low risk of bias | Minimal missing outcome data but unclear how it impacts the results. |
| Selective Outcome Reporting | Low risk of bias | All of the study’s pre-specified (primary and secondary) outcomes outlined in the protocol, methods, abstract, and/or introduction that are of interest have been reported in the pre-specified way. |
| Conflict of Interest | Low risk of bias | The authors declare that they have no competing financial interests or personal relationships that could influence the work. |
| Other | Low risk of bias | The study appears to be free of other sources of bias. |

**Table 16: Pocock, 1987**

| Domain | Risk of Bias Rating | Explanation |
| --- | --- | --- |
| Selection Bias | Probably low risk of bias | There is insufficient information about participant selection to permit a judgment of low risk of bias, but there is indirect evidence which suggests that inclusion/exclusion criteria, recruitment and enrollment procedures, as well as participation and follow-up rates were consistent across groups as described by the criteria for a judgment of low risk of bias (See Smith et al., 1983). |
| Blinding | Probably low risk of bias | The review authors judge that the outcome and exposure measures are not likely to be influenced by a lack of blinding. The exposure was measured by a separate entity to the outcome |
| Exposure Assessment | Probably high risk of bias | There is insufficient information about the exposure assessment methods. As noted in the following text: "Tooth lead estimations were made using atomic absorption spectroscopy on whole tooth crowns, such analysis being on duplicate portions." There is no additional QA/QC reported and no citations. |
| Outcome Assessment | Low risk of bias | Outcomes were assessed and defined consistently across all study participants, using valid and reliable measures of intelligence (WISC-R) |
| Confounding | Probably high risk of bias | The study evaluated most but not all the important confounders (Tier I), but did not evaluate multiple other potentially important confounders (Tier II). Confounders considered include: sex, social group, family size, neonatal problems, mothers IQ, quality of marital relationship, family characteristics, mother’s mental health, social background, parental education, parental interest and attitude |
| Incomplete Outcome Data | Low risk of bias | “Of the total participant population, eight children moved out of the area, six refused, six never responded, and nine more were subsequently found to be ineligible.” |
| Selective Outcome Reporting | Low risk of bias | All of the study’s pre-specified (primary and secondary) outcomes outlined in the protocol, methods, abstract, and/or introduction that are of interest have been reported in the pre-specified way. |
| Conflict of Interest | Low risk of bias | The study did not receive support from a company, study author, or other entities having a financial interest in the outcome of the study. |
| Other | Low risk of bias | The study appears to be free of other sources of bias. |

**Table 17: Tatsuta, 2020**

| Domain | Risk of Bias Rating | Explanation |
| --- | --- | --- |
| Selection Bias | Low risk of bias | The descriptions of the source population, inclusion/exclusion criteria, recruitment and enrollment procedures, as well as participation and follow-up rates were sufficiently detailed, and adequate data were supplied on the distribution of relevant study sample and population characteristics to support the assertion that risk of selection effects was minimal. |
| Blinding | Low risk of bias | As supported by the following text: “The testers were unaware of the exposure status of the participants.” |
| Exposure Assessment | Probably low risk of bias | The reviewers judge that there is low risk of exposure misclassification, i.e., There is high confidence in the accuracy of the exposure assessment methods, such as methods that have been tested for validity and reliability in measuring the targeted exposure. Appropriate QA/QC for methods are described and are satisfactory. However, there is only one prenatal measurement of lead described. |
| Outcome Assessment | Low risk of bias | Outcomes were assessed and defined consistently across all study participants, using valid and reliable measures of intelligence (Japanese-version of the WISC-IV) |
| Confounding | Probably low risk of bias | The study evaluated some but not all the important confounders (Tier I) and some but not all the other potentially important confounders relevant (Tier II). Confounders considered include birth weight, drinking, smoking, Raven score, income, testers, and cord blood mercury. |
| Incomplete Outcome Data | Low risk of bias | Reasons for missing outcome data are unlikely to be related to true outcome, as shown in Figure 1. |
| Selective Outcome Reporting | Low risk of bias | All of the study’s pre-specified (primary and secondary) outcomes outlined in the protocol, methods, abstract, and/or introduction that are of interest have been reported in the pre-specified way. |
| Conflict of Interest | Low risk of bias | The study did not receive support from a company, study author, or other entities having a financial interest in the outcome of the study. |
| Other | Low risk of bias | The study appears to be free of other sources of bias |

**Table 18: Taylor, 2017**

| Domain | Risk of Bias Rating | Explanation |
| --- | --- | --- |
| Selection Bias | Probably high risk of bias | A subsample of children was chosen at random to be tested on IQ at age 4 -, the method of randomization was not disclosed, and they found significant differences between those included at age 4 versus those not included at age 4. Whether lead levels differed in mothers with children with and without outcome data is unclear. |
| Blinding | Probably low risk of bias | No blinding was reported in the study, but the review authors judge that the outcome and exposure measures are not likely to be influenced by lack of blinding. Whole blood samples were collected by midwives and measured for lead in a laboratory setting, whereas trained psychologists administered IQ tests in a health clinic. |
| Exposure Assessment | Probably low risk of bias | Lead was measured in whole blood samples. The limit of detection is discussed, and a source is cited with indirect evidence regarding how blood levels were measured, accompanied by appropriate quality controls (see Taylor et al, 2013). |
| Outcome Assessment | Low risk of bias | Intelligence was assessed and defined consistently across all study participants, using a valid and reliable measure (WPPSI and WISC-III UK edition). Trained psychologists administered all tests. |
| Confounding | Probably low risk of bias | The study appropriately assessed and considered for most but not all of the important confounders (Tier I), and some but not all potentially important confounders relevant (Tier II). The study accounted for family adversity, housing tenure, household crowding, smoking in the first trimester, alcohol consumption in the first trimester, maternal age, parity, maternal education, length of time the mother had lived in Avon, child sex, child age at testing, weighted life events score and hemoglobin level. Birth weight and gestational age were not included, as they are considered to be on the common pathway to IQ. |
| Incomplete Outcome Data | Low risk of bias | Missing data were imputed using the Multiple Imputation function in SPSS. Results are shown in a parallel paper (Taylor et al., 2017). |
| Selective Outcome Reporting | Low risk of bias | All of the study’s pre‐specified outcomes outlined in the methods that are of interest have been reported in the pre‐specified way. |
| Conflict of Interest | Low risk of bias | Study authors make a claim denying conflicts of interest |
| Other | Low risk of bias | The study appears to be free of other sources of bias. |

**Table 19: Torres-Olascoaga, 2020**

| Domain | Risk of Bias Rating | Explanation |
| --- | --- | --- |
| Selection Bias | Low risk of bias | The descriptions of the source population, inclusion/exclusion criteria, recruitment and enrollment procedures, as well as participation and follow-up rates were sufficiently detailed, and adequate data were supplied on the distribution of relevant study sample and population characteristics to support the assertion that risk of selection effects was minimal. |
| Blinding | Low risk of bias | As supported by the following text: “The MSCA was administered by trained and standardized psychologists, who were blind to participants’ phthalate metabolite concentrations." |
| Exposure Assessment | Probably low risk of bias | There may be insufficient information about the exposure assessment methods to permit a judgement of low risk of bias, however, there is indirect evidence, which suggests that methods were robust, as described by the criteria for a judgement of low risk of bias (see Lewis et al., 2013). |
| Outcome Assessment | Low risk of bias | Outcomes were assessed and defined consistently across all study participants, using valid and reliable measures of intelligence (MSCA) |
| Confounding | High risk of bias | The study did not account for multiple important confounders (Tier I), and multiple other potentially important confounders relevant (Tier II). Confounders considered include sex, gestational age, birth weight and length, breastfeeding, mothers age, education, and IQ, and HOME scores. |
| Incomplete Outcome Data | Low risk of bias | No missing outcome data from the subsample. |
| Selective Outcome Reporting | Low risk of bias | All of the study’s pre-specified (primary and secondary) outcomes outlined in the protocol, methods, abstract, and/or introduction that are of interest have been reported in the pre-specified way. |
| Conflict of Interest | Low risk of bias | The authors declare no conflict of interest. |
| Other | Probably low risk of bias | Limited number of women who had archived urinary samples to measure phthalate metabolites, which likely limited power to detect interactions with sex. |

**Table 20: van den Dries, 2020**

| Domain | Risk of Bias Rating | Explanation |
| --- | --- | --- |
| Selection Bias | Probably low risk of bias | The descriptions of the source population, inclusion/exclusion criteria, recruitment and enrollment procedures, as well as participation and follow-up rates, were sufficiently detailed. Women in this subset had higher education and income levels, were slightly older, and were more likely to be of Dutch national origin than the broader Generation R cohort (Kooijman et al. 2016). |
| Blinding | Probably low risk of bias | The review authors judge that the outcome and exposure measures are not likely to be influenced by a lack of blinding. |
| Exposure Assessment | Probably low risk of bias | There is high confidence in the accuracy of the exposure assessment methods, such as methods that have been tested for validity and reliability in measuring the targeted exposure (See Philips et al., 2018 for more details), although phthalates were adjusted for creatinine instead of SG |
| Outcome Assessment | Low risk of bias | Outcomes were assessed and defined consistently across all study participants, using valid and reliable measures of intelligence (SON-R) |
| Confounding | Probably low risk of bias | The study appropriately assessed and considered most of the important confounders (Tier I), and most of the other potentially important confounders (Tier II). Confounders considered include: BMI, maternal age, parity, smoking, alcohol intake, marital status, household total net income, highest completed education level, ethnicity, folic acid intake, maternal IQ, and consumption of fruits and vegetables |
| Incomplete Outcome Data | Probably low risk of bias | All missing covariate data were imputed 10 times with the multivariate imputation by chained equations method in R. However, the outcome variable child nonverbal IQ was not imputed. |
| Selective Outcome Reporting | Low risk of bias | All of the study’s pre-specified (primary and secondary) outcomes outlined in the protocol, methods, abstract, and/or introduction that are of interest have been reported in the pre-specified way |
| Conflict of Interest | Low risk of bias | This research received financial support from government grants |
| Other | Low risk of bias | The study appears to be free of other sources of bias. |
