# Supplementary material for: Sex difference of pre- and post-natal exposure to six developmental neurotoxicants on intellectual abilities: a systematic review and meta-analysis of human studies
Source: Environ Health. 2023 Nov 17;22:80. doi: 10.1186/s12940-023-01029-z (PMC10655280; doi:10.1186/s12940-023-01029-z)
Supplement: Supplementary file 5 — Additional file 5: Sensitivity Analyses Figure 1. Funnel Plot - General Intelligence in Males. Figure 2. Funnel Plot - General Intelligence in Females. Figure 3. Funnel Plot - Nonverbal Intelligence in Males. Figure 4. Funnel Plot - Nonverbal Intelligence in Females. Figure 5. Funnel Plot - Verbal Intelligence in Males. Figure 6. Funnel Plot - Verbal Intelligence in Females. Figure 7. Funnel Plot - Postnatal Lead and General Intelligence in Males. Figure 8. Funnel Plot - Postnatal Lead and General Intelligence in Females. Figure 9. Leave One Out - General Intelligence in Males. Figure 10. Leave One Out - General Intelligence in Females. Figure 11. Leave One Out - Nonverbal Intelligence in Males. Figure 12. Leave One Out - Nonverbal Intelligence in Females. Figure 13. Leave One Out - Verbal Intelligence in Males. Figure 14. Leave One Out - Verbal Intelligence in Females. Figure 15. Leave One Out - Postnatal Lead and General Intelligence in Males. Figure 16. Leave One Out - Postnatal Lead and General Intelligence in Females. Figure 17. Low Risk of Bias - General Intelligence in Males. Figure 18. Low Risk of Bias - General Intelligence in Females. Figure 19. Low Risk of Bias - Nonverbal Intelligence in Males. Figure 20. Low Risk of Bias - Nonverbal Intelligence in Females. Figure 21. Low Risk of Bias - Verbal Intelligence in Males. Figure 22. Low Risk of Bias - Verbal Intelligence in Females. [file 12940_2023_1029_MOESM5_ESM.docx]

**Supplemental Figures**


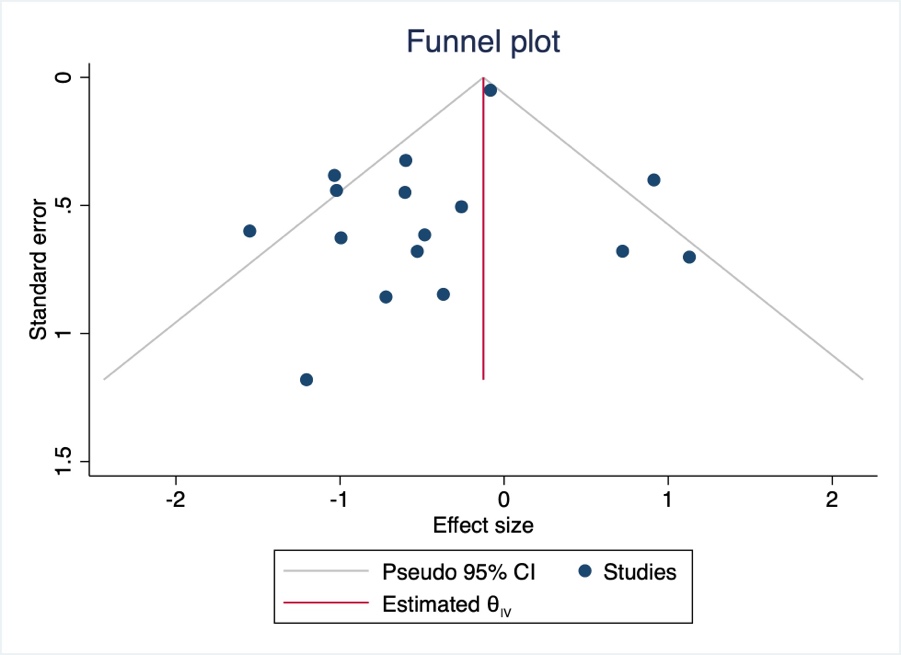


Supplemental Figure 1. Funnel Plot - General Intelligence in Males


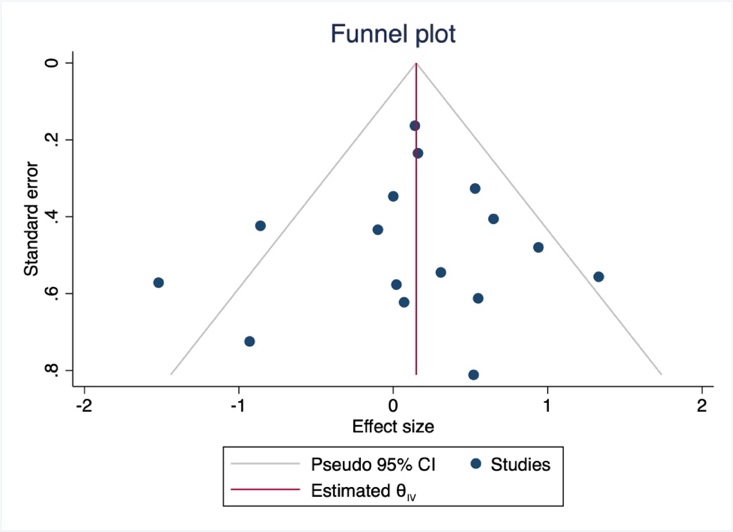


Supplementary Figure 2. Funnel Plot - General Intelligence in Females


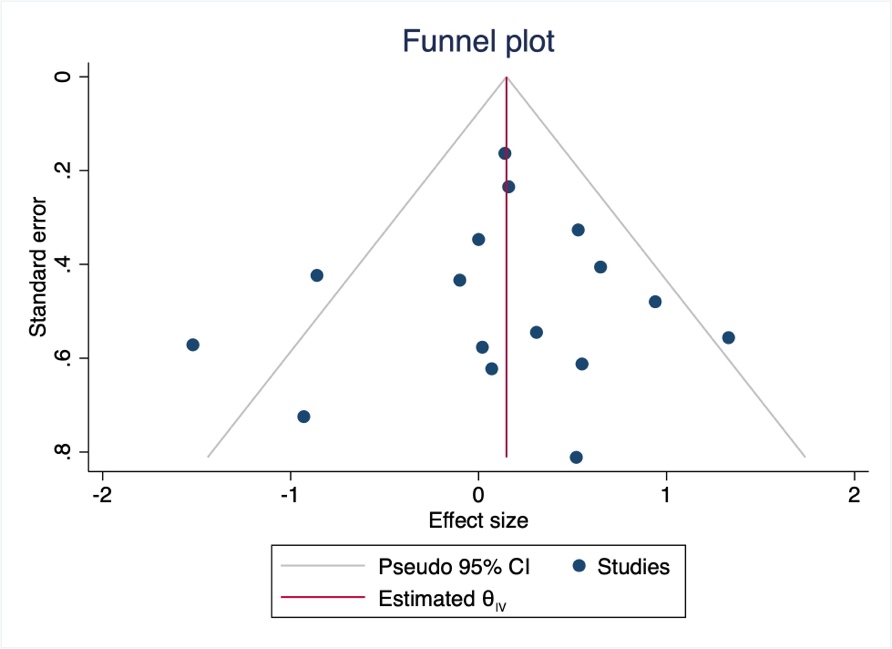


Supplementary Figure 3. Funnel Plot - Nonverbal Intelligence in Males


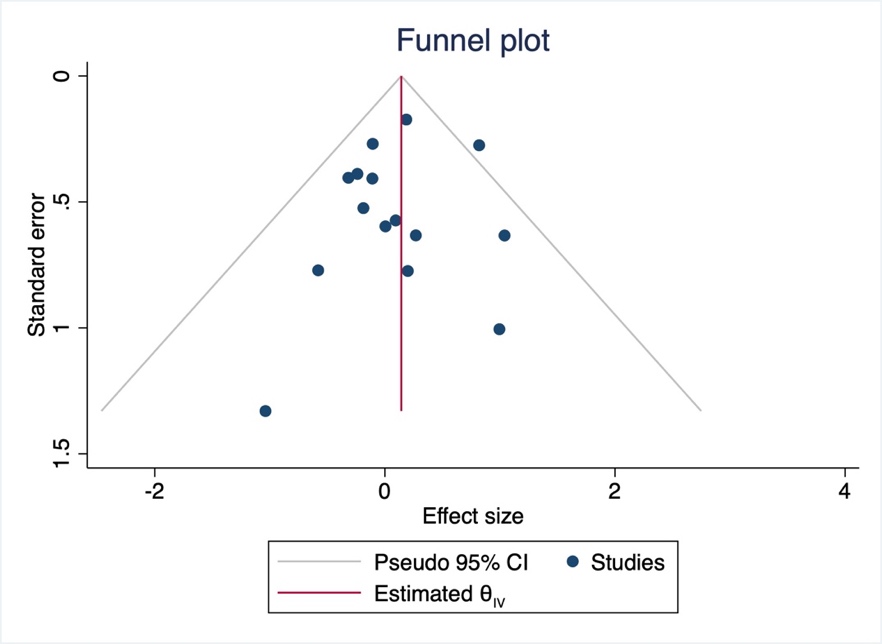


Supplementary Figure 4. Funnel Plot - Nonverbal Intelligence in Females


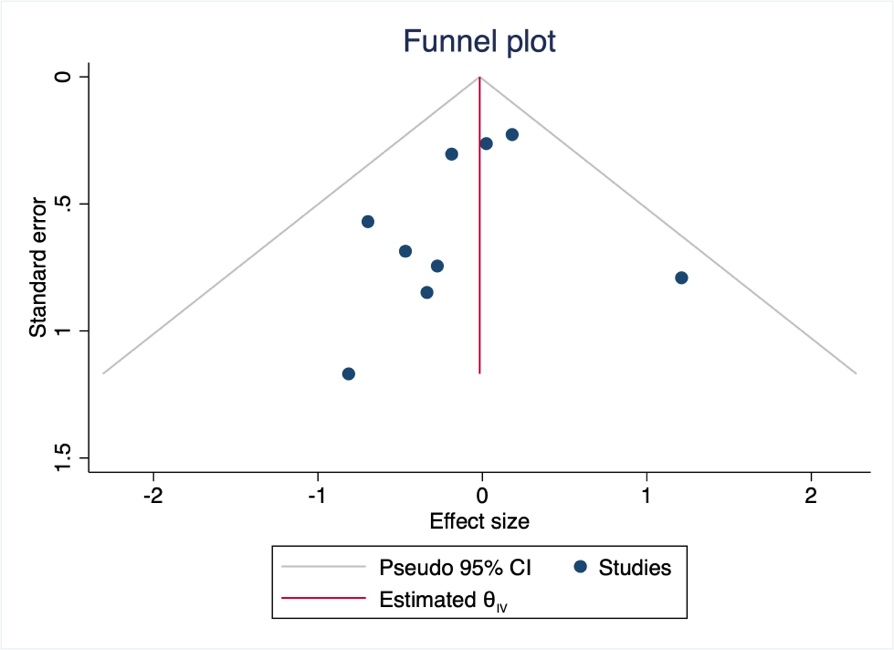


Supplementary Figure 5. Funnel Plot - Verbal Intelligence in Males


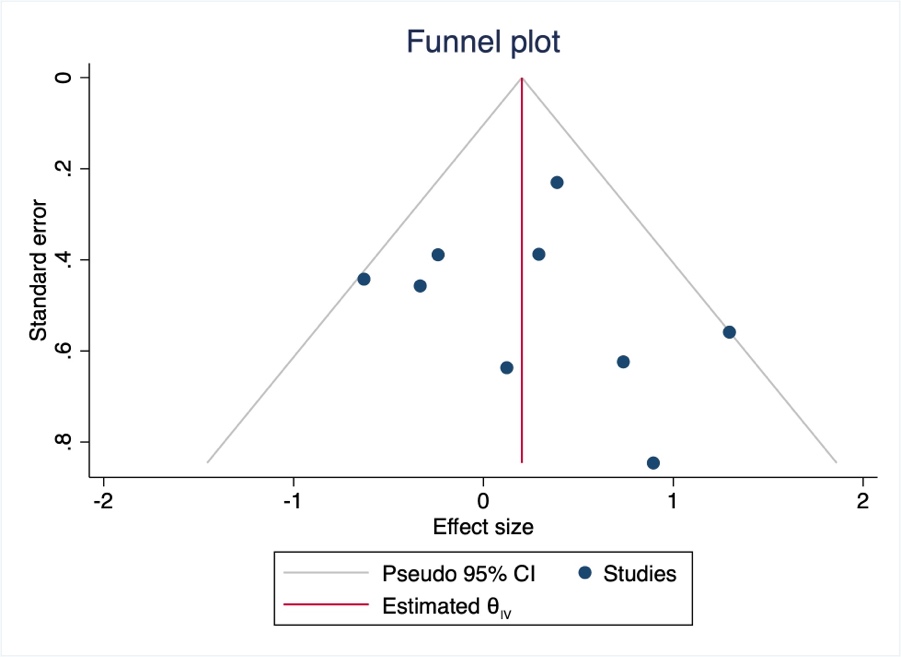


Supplementary Figure 6. Funnel Plot - Verbal Intelligence in Females


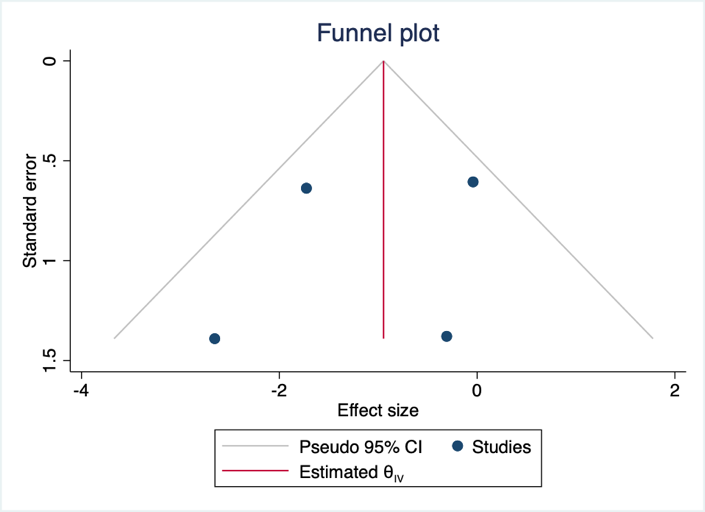


Supplementary Figure 7. Funnel Plot - Postnatal Lead and General Intelligence in Males


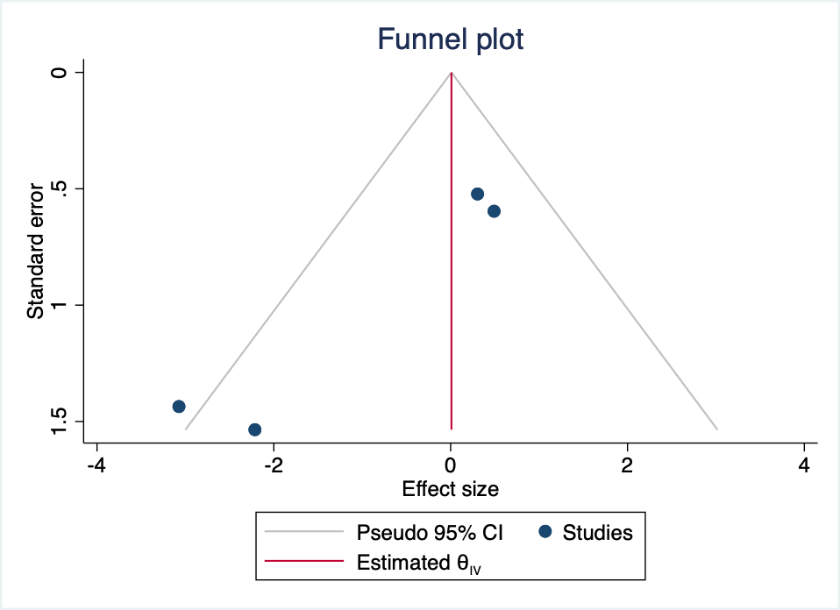


Supplementary Figure 8. Funnel Plot - Postnatal Lead and General Intelligence in Females


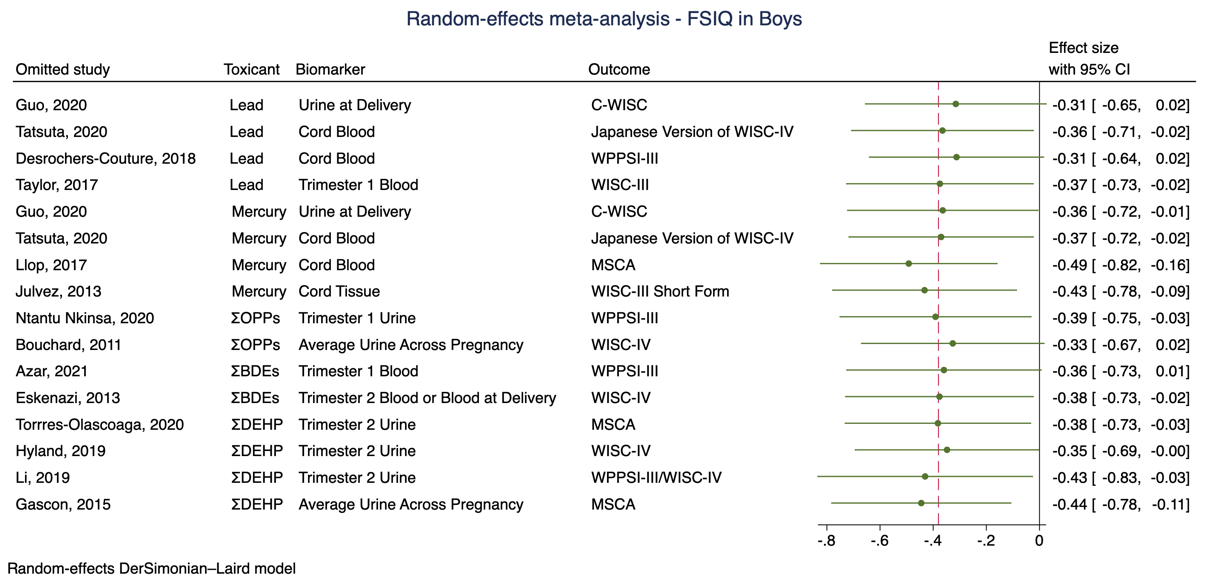
Leave-one-out meta-analysis

Supplementary Figure 9. Leave One Out - General Intelligence in Males


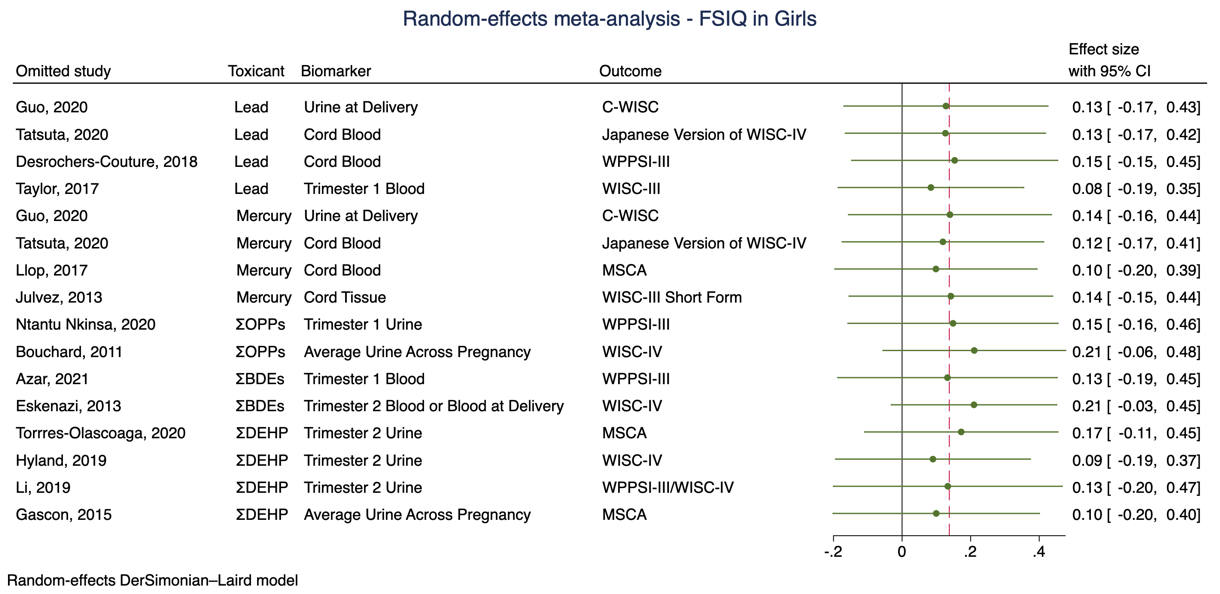


Supplementary Figure 10. Leave One Out - General Intelligence in Females


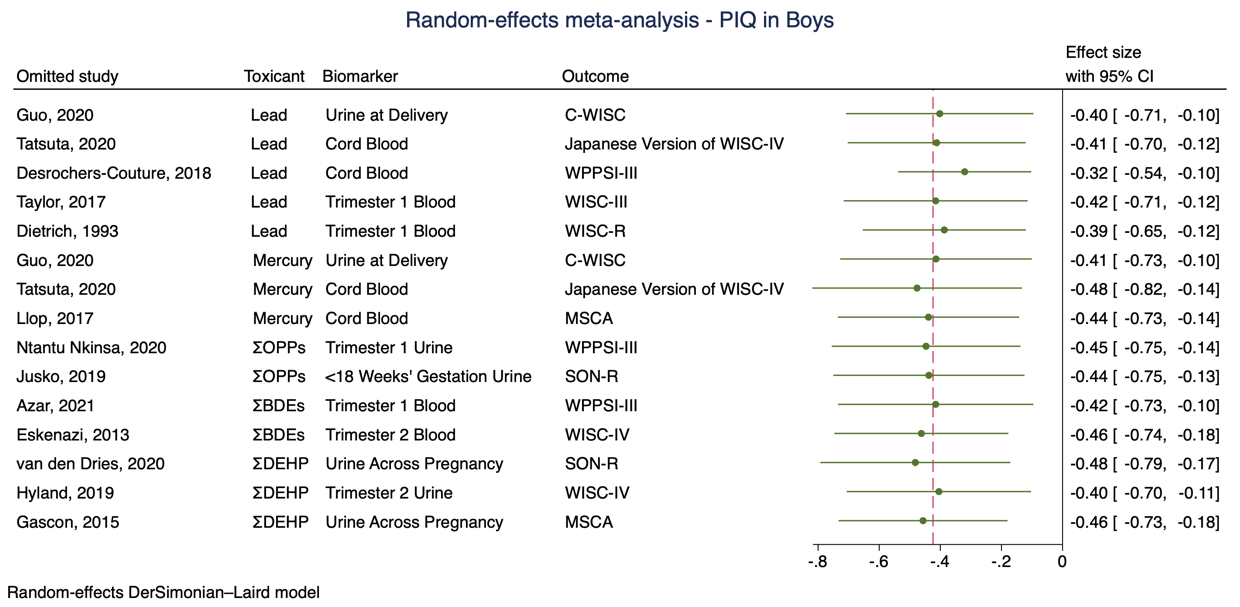


Supplementary Figure 11. Leave One Out - Nonverbal Intelligence in Males


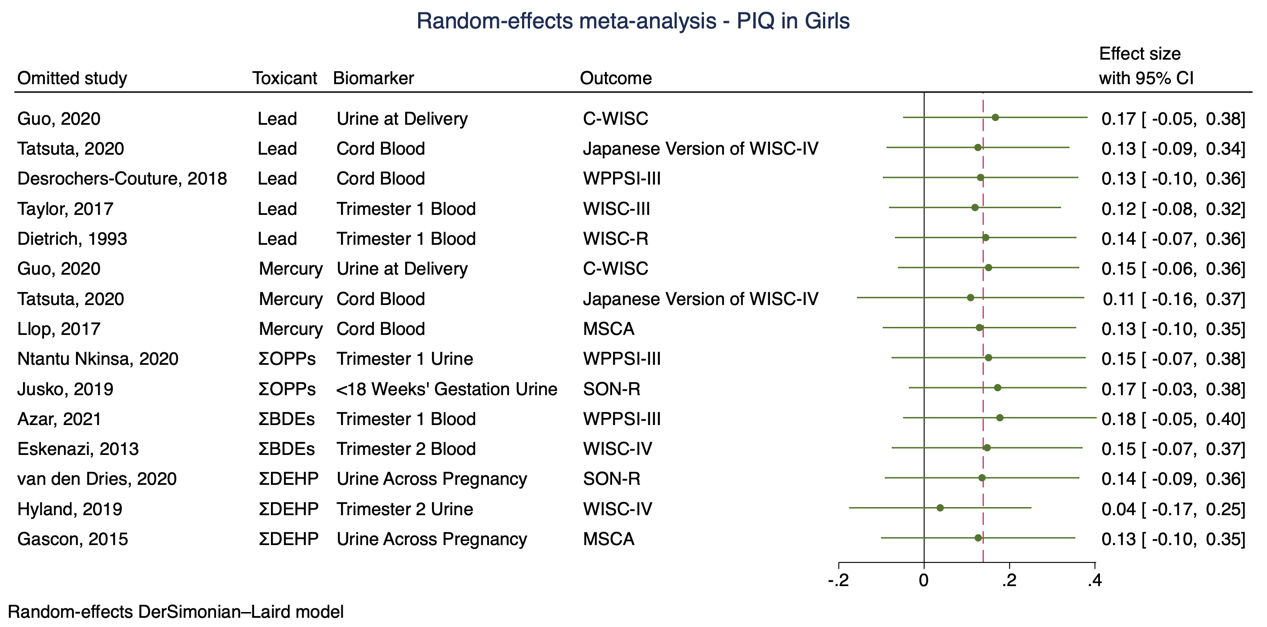


Supplementary Figure 12. Leave One Out - Nonverbal Intelligence in Females


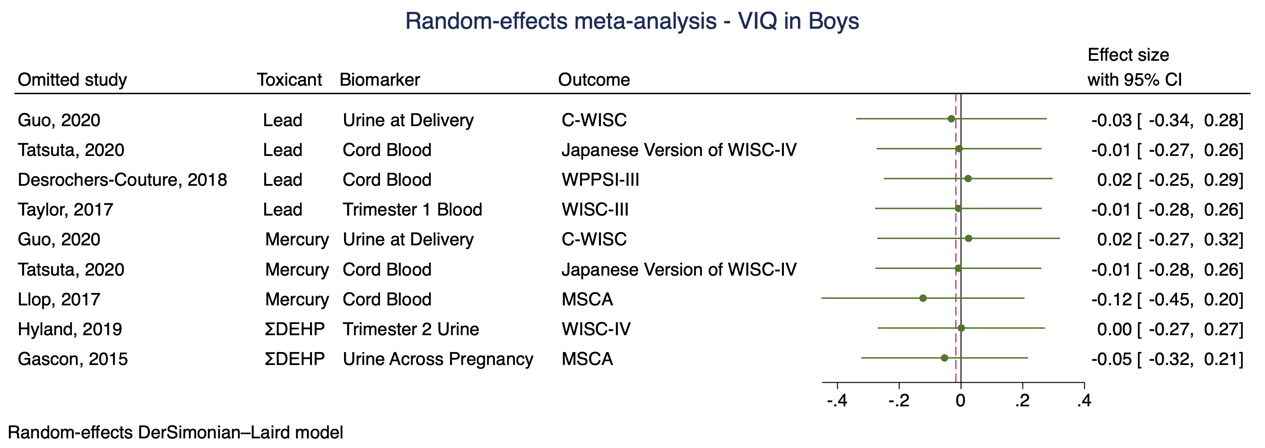


Supplementary Figure 13. Leave One Out - Verbal Intelligence in Males


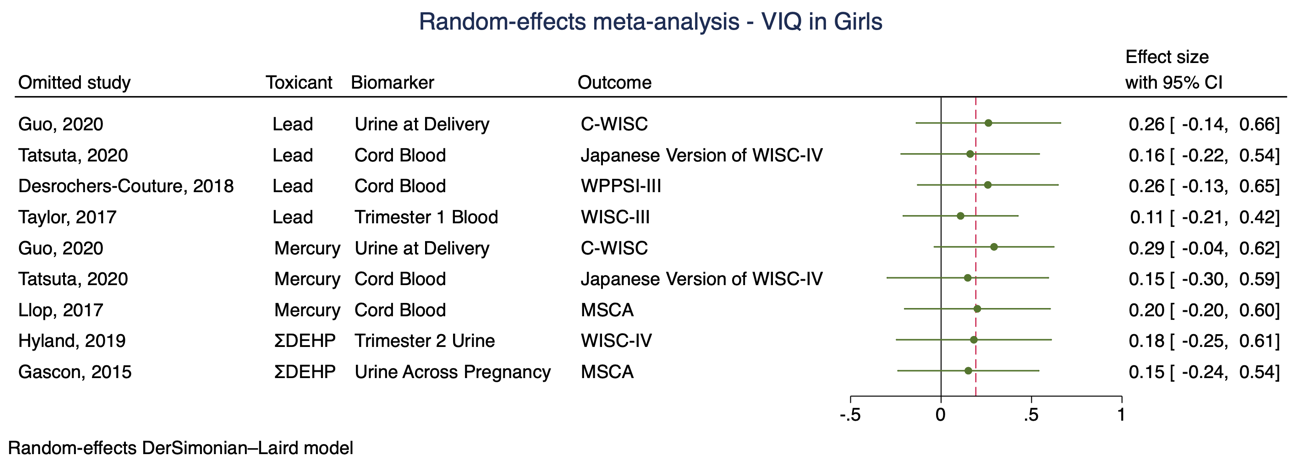


Supplementary Figure 14. Leave One Out - Verbal Intelligence in Females

***
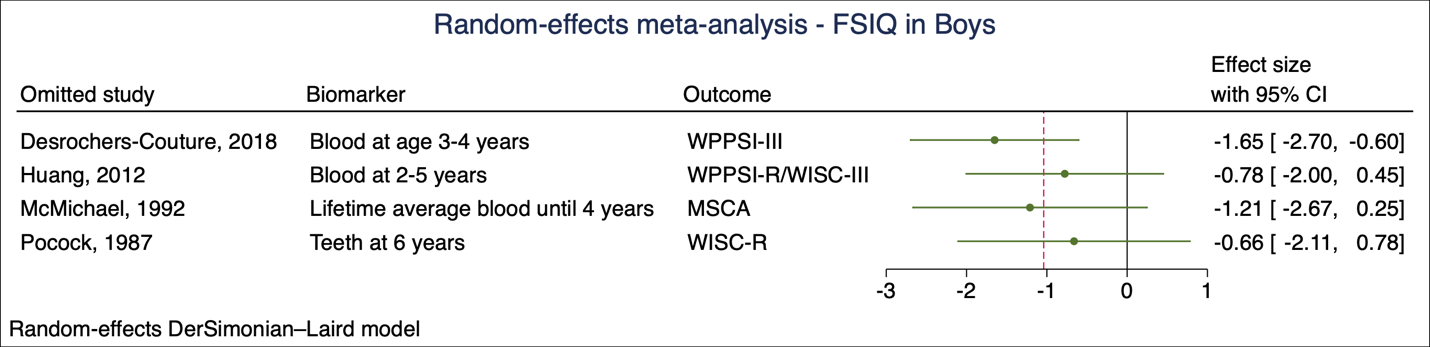
***

Supplementary Figure 15. Leave One Out - Postnatal Lead and General Intelligence in Males


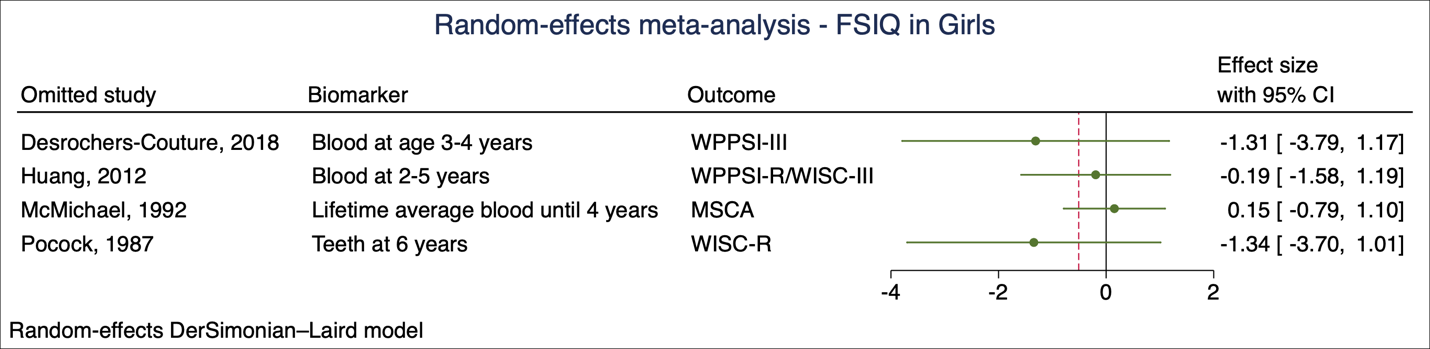


Supplementary Figure 16. Leave One Out - Postnatal Lead and General Intelligence in Females

**
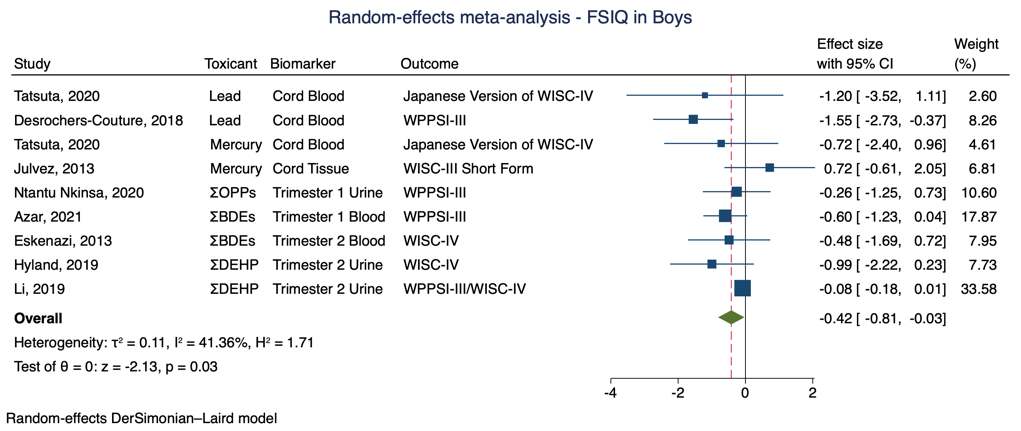
**

Supplementary Figure 17. Low Risk of Bias - General Intelligence in Males

**
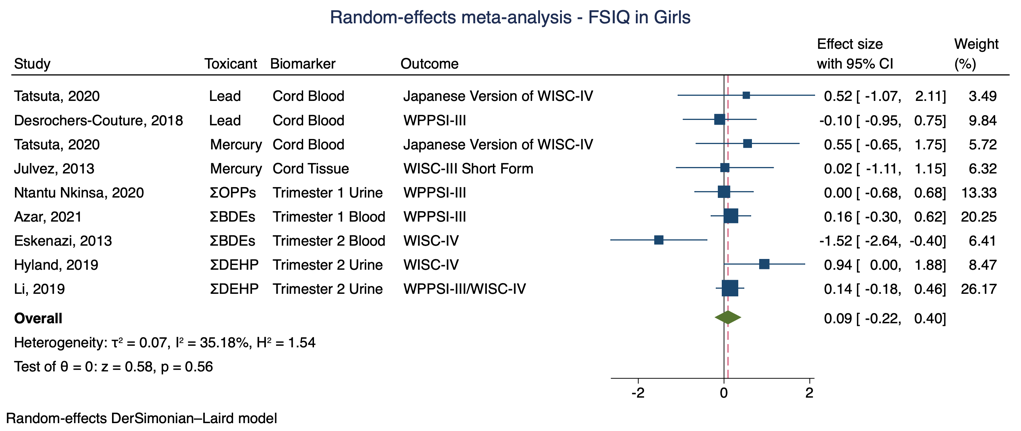
**

Supplementary Figure 18. Low Risk of Bias - General Intelligence in Females


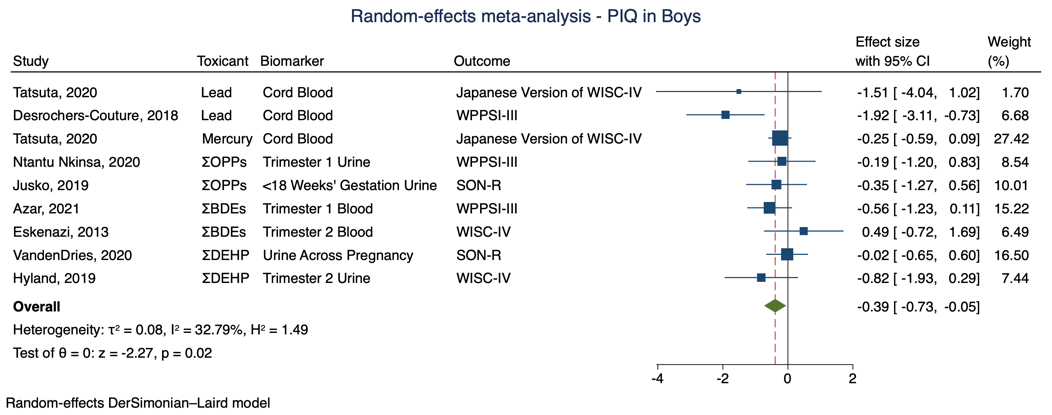


Supplementary Figure 19. Low Risk of Bias - Nonverbal Intelligence in Males


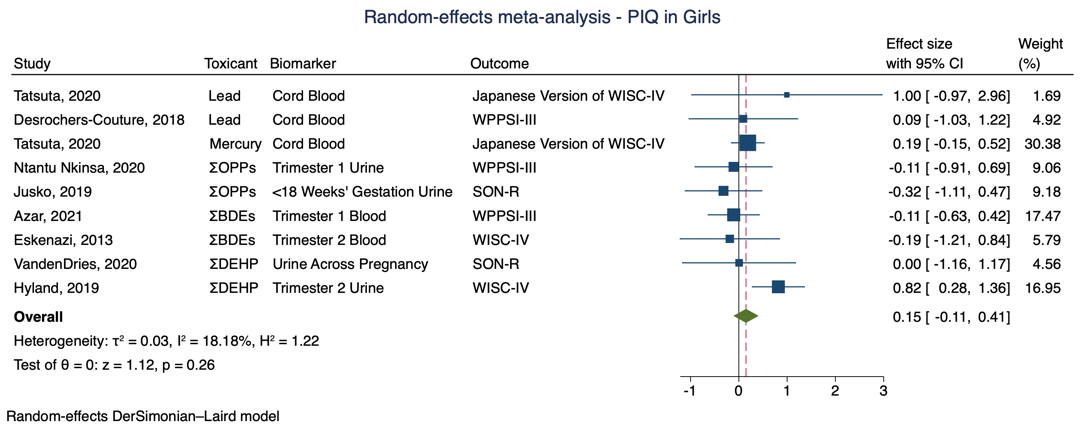


Supplementary Figure 20. Low Risk of Bias - Nonverbal Intelligence in Females


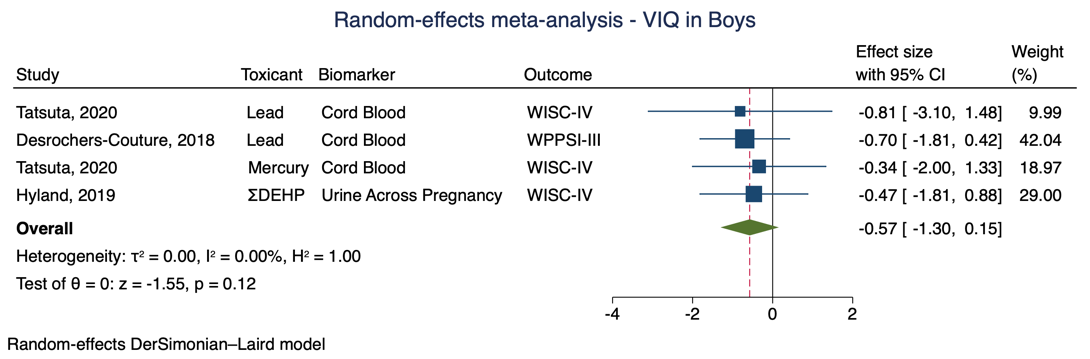


Supplementary Figure 21. Low Risk of Bias - Verbal Intelligence in Males


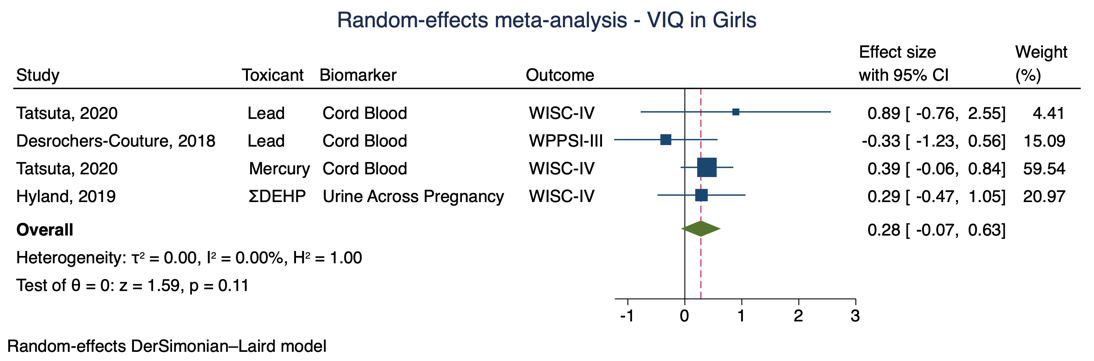


Supplementary Figure 22. Low Risk of Bias - Verbal Intelligence in Females
