# Supplementary material for: Sex difference of pre- and post-natal exposure to six developmental neurotoxicants on intellectual abilities: a systematic review and meta-analysis of human studies
Source: Environ Health. 2023 Nov 17;22:80. doi: 10.1186/s12940-023-01029-z (PMC10655280; doi:10.1186/s12940-023-01029-z)
Supplement: Supplementary file 6 — Additional file 6. Rationale for Risk of Bias Determinations for Each Study Included in the Narrative Synthesis. [file 12940_2023_1029_MOESM6_ESM.docx]

**Supplementary Tables:**

**Rationale for Risk of Bias Determinations for Each Study Included in the Narrative Synthesis**

**Table 1: Baghurst, 1992**

| Domain | Risk of Bias Rating | Explanation |
| --- | --- | --- |
| Selection Bias | Probably low risk of bias | There is insufficient information about participant selection to permit a judgment of low risk of bias, however, there is indirect evidence which suggests that inclusion/exclusion criteria, recruitment and enrollment procedures, and participation and follow-up rates were consistent across groups as described by the criteria for a judgment of low risk of bias (See McMichael et al., 1985, and Baghurst et al., 1985). |
| Blinding | Low risk of bias | As supported by the text: “All children were evaluated by the same research psychologist who was unaware of each child's lead exposure status" |
| Exposure Assessment | Probably low risk of bias | There may be insufficient information about the exposure assessment methods to permit a judgement of low risk of bias, but there is indirect evidence which suggests that the methods were robust (see McMichael et al., 1985). Lead was measured in whole blood, taking the mean of serial measurements. |
| Outcome Assessment | Low risk of bias | Outcomes were assessed and defined consistently across all study participants, using valid and reliable measures (i.e., WISC-R). |
| Confounding | Probably high risk of bias | The study appropriately considered most of the important confounders (Tier I) but did not account for multiple other potentially important confounders relevant (Tier II). Confounders considered include SES, HOME environment, maternal IQ, parental smoking, years of education, whether parents were living together, birth weight, birth order, and breastfeeding. |
| Incomplete Outcome Data | Low risk of bias | Minimal missing outcome data. |
| Selective Outcome Reporting | Low risk of bias | All of the study’s pre-specified (primary and secondary) outcomes outlined in the protocol, methods, abstract, and/or introduction that are of interest have been reported in the pre-specified way. |
| Conflict of Interest | Probably low risk of bias | There is insufficient information to permit a judgment of low risk of bias, but there is indirect evidence which suggests the study was free of support from a company, study author, or other entities having a financial interest in the outcome of the study, as described by the criteria for a judgment of low risk of bias. |
| Other | Low risk of bias | The study appears to be free of other sources of bias. |

**Table 2: Berghuis, 2018**

| Domain | Risk of Bias Rating | Explanation |
| --- | --- | --- |
| Selection Bias | Probably high risk of bias | There are indirect details of the recruitment process and inclusion/exclusion criteria of the original cohort. There is sufficient information described about the sample invited to participate. The participating adolescents of the GIC cohort had significantly higher total intelligence at 5–6 years of age compared to the children who were not participating at follow-up (~104 vs ~99). |
| Blinding | Low risk of bias | As supported by the text: “All assessors were blind to prenatal POP levels.” |
| Exposure Assessment | Probably low risk of bias | Maternal serum samples were collected during the second and/or third trimester of pregnancy. There is insufficient information about the QA/QC, but there is indirect evidence, which suggests that methods were robust, as described by the criteria for a judgement of low risk of bias (i.e., Meijer et al., 2008; Soechitram et al., 2004). |
| Outcome Assessment | Low risk of bias | Outcomes were assessed and defined consistently across all study participants, using valid and reliable measures of intelligence (WISC-III-NL) |
| Confounding | High risk of bias | The study did not account for multiple important confounders (Tier I), and multiple other potentially important confounders relevant (Tier II). Confounders considered include maternal education, maternal smoking and alcohol consumption, breastfeeding and age at examination. |
| Incomplete Outcome Data | Probably low risk of bias | “There was no difference between the participating and non-participating adolescents regarding maternal education level and POP levels, except for PBDE-154 levels, which were lower in the participating children….there were seven children missing performance and total IQs due to the following reasons: four children performed a subtest of the WISC < 12 months before current testing; two children had a very low score on a performance subtest, most likely due to too much emphasis on speed and insufficient emphasis on accuracy; and for one child, an error in testing procedure occurred.” These reasons are unlikely to be related to the true outcome |
| Selective Outcome Reporting | Low risk of bias | All of the study’s pre‐specified outcomes outlined in the methods that are of interest have been reported in the pre‐specified way. |
| Conflict of Interest | Low risk of bias | The authors declare they have no competing financial interests |
| Other | Probably low risk of bias | Increased risk for a Type I error due to the large number of tests performed. However, associations are generally consistent. |

**Table 3: Castorina, 2017**

| Domain | Risk of Bias Rating | Explanation |
| --- | --- | --- |
| Selection Bias | Probably low risk of bias | There is indirect information regarding the recruitment process and follow-up participation (Eskenazi et al., 2004, 2006). Selection factors appeared to be well-understood and were measured in the data set. Appropriate adjustment post hoc techniques were used to control for selection bias. |
| Blinding | Low risk of bias | As supported by the following text: “The psychometrician was blinded to exposure status." |
| Exposure Assessment | Probably low risk of bias | Used urine instead of serum to measure OPPs and only one urine sample. However, there is indirect evidence which suggests that QA/QC was robust, as described by the criteria for a judgment of low risk of bias (see Castorina et al., 2017). |
| Outcome Assessment | Low risk of bias | Outcomes were assessed and defined consistently across all study participants, using valid and reliable measures (WISC-IV) |
| Confounding | Probably high risk of bias | The study appropriately considered most of the important confounders (Tier I) and some of the other potentially important confounders (Tier II). Confounders considered include the child's age at assessment, sex, maternal country of birth, HOME score, maternal age, maternal IQ, education, marital status, depression, housing density, poverty, presence of father in the home, maternal work status, and location of assessment |
| Incomplete Outcome Data | Probably low risk of bias | Excluded children with medical conditions that could affect neurodevelopmental assessment. Out of 330 children followed until age 7, a Full-Scale IQ was available for 268 children with maternal urinary PFR metabolite measurements. Unclear how this missing outcome data impacted the results |
| Selective Outcome Reporting | Low risk of bias | All of the study’s pre‐specified outcomes outlined in the methods that are of interest have been reported in the pre‐specified way. |
| Conflict of Interest | High risk of bias | Dr. Asa Bradman is a volunteer member of the Board for The Organic Center. None of the other authors declares any competing financial interest. |
| Other | Low risk of bias | The study appears to be free of other sources of bias. |

**Table 4: Chen, 2014**

| Domain | Risk of Bias Rating | Explanation |
| --- | --- | --- |
| Selection Bias | Low risk of bias | The descriptions of the source population, inclusion/exclusion criteria, recruitment process and enrollment procedures, participation and follow-up rates were sufficiently detailed. |
| Blinding | Low risk of bias | As supported by the following text: “The assessors conducted the neurobehavioral assessments without knowledge of maternal PBDE levels". |
| Exposure Assessment | Low risk of bias | PBDEs were measured in serum and lipid adjusted. There is high confidence in the accuracy of the exposure assessment methods, such as methods that have been tested for validity and reliability in measuring the targeted exposure. |
| Outcome Assessment | Low risk of bias | Outcomes were assessed and defined consistently across all study participants, using valid and reliable measures (WPPSI-III) |
| Confounding | Low risk of bias | The study appropriately considered all important confounders (Tier I) and most other potentially important confounders (Tier II). Confounders considered: maternal age at enrollment, maternal race/ethnicity, education, marital status, maternal serum cotinine concentrations at enrollment, maternal IQ, maternal depression, household income, HOME score, and maternal blood lead |
| Incomplete Outcome Data | Low risk of bias | “The 190 child participants who had an IQ test at age 5 were not statistically different in maternal PBDE concentrations and Bayley scores at ages 1–3 years from the 119 participants who did not complete IQ test at the age of 5 years.” |
| Selective Outcome Reporting | Probably low risk of bias | One outcome (PDI<85) was not reported, but otherwise all of the study’s pre‐specified outcomes outlined in the methods that are of interest have been reported in the pre‐specified way. |
| Conflict of Interest | Low risk of bias | The authors declare they have no competing financial interests. |
| Other | Low risk of bias | The study appears to be free of other sources of bias. |

**Table 5: Choi, 2021**

| Domain | Risk of Bias Rating | Explanation |
| --- | --- | --- |
| Selection Bias | Probably low risk of bias | The descriptions of the source population, inclusion/exclusion criteria, recruitment process and enrollment procedures, and participation and follow-up rates were sufficiently detailed. The subsample involved an oversampling of children with ADHD; however, they used a weighted analysis to combat this. |
| Blinding | Probably low risk of bias | The review authors judge that the outcome and exposure measures are not likely to be influenced by lack of blinding. The exposure was measured by a separate entity and the outcome measure was obtained from a hospital record. |
| Exposure Assessment | Probably low risk of bias | Phthalates were measured in one urine sample. There may be insufficient information about the exposure assessment methods to permit a judgement of low risk of bias. However, there is indirect evidence, which suggests that methods were robust, as described by the criteria for a judgement of low risk of bias (See Engel, 2018 & Ye at al., 2009). |
| Outcome Assessment | Low risk of bias | Outcomes were assessed and defined consistently across all study participants, using valid and reliable measures (stanford-binet 5) |
| Confounding | Probably high risk of bias | The study did not account for multiple important confounders (Tier I), but did account for most of the potentially important confounders. Confounders considered included maternal ADHD symptoms, parity, marital status, education, pre-pregnancy BMI, self-reported depression, smoking, alcohol intake, and fish intake during pregnancy, folate use during pregnancy, maternal age at birth, childbirth year, child sex, and child age at the clinical exam. |
| Incomplete Outcome Data | Probably low risk of bias | “Since missing data was present, considered omitting some potential confounders from the minimally sufficient adjustment set in order to improve variance and selection bias” |
| Selective Outcome Reporting | Low risk of bias | All of the study’s pre-specified outcomes outlined in the protocol, methods, abstract, and/or introduction that are of interest have been reported in the pre-specified way. |
| Conflict of Interest | Low risk of bias | Study authors make a claim denying conflicts of interest |
| Other | Low risk of bias | The study appears to be free of other sources of bias. |

**Table 6: Damm, 1993**

| Domain | Risk of Bias Rating | Explanation |
| --- | --- | --- |
| Selection Bias | Low risk of bias | The descriptions of the source population, inclusion/exclusion criteria, recruitment and enrolment procedures, participation and follow-up rates were sufficiently detailed. The attrition was almost evenly distributed between the high-lead and low-lead groups. The average lead concentrations within the two lead groups were virtually unchanged. |
| Blinding | Low risk of bias | As supported by the following text, “The psychological testing…was performed by a single clinical psychologist who was blind to all data regarding lead exposure.” |
| Exposure Assessment | Probably low risk of bias | Lead was measured in toot dentin. There may be insufficient information about the QA/QC methods to permit a judgement of low risk of bias. However, there is indirect evidence, which suggests that methods were robust (See Grandjean et al., 1978). |
| Outcome Assessment | Low risk of bias | Outcomes were assessed and defined consistently across all study participants, using valid and reliable measures (WISC - Danish version) |
| Confounding | Probably high risk of bias | The study accounted for some important confounders (Tier I) and some other potentially important confounders (Tier II). Confounders considered include neonatal jaundice, maternal education, birth before Week 36, maternal smoking during pregnancy, maternal age adjusted for parity, birth order, and left-handedness. Sex of the child and paternal SES had been used as matching variables in the original selection of the children. |
| Incomplete Outcome Data | Low risk of bias | The reasons for missing outcome data are unlikely to be related to true outcome |
| Selective Outcome Reporting | Low risk of bias | All of the study’s pre-specified outcomes outlined in the protocol, methods, abstract, and/or introduction that are of interest have been reported in the pre-specified way. |
| Conflict of Interest | Low risk of bias | This study was supported by the Danish Medical Research Council. |
| Other | Low risk of bias | The study appears to be free of other sources of bias. |

**Table 7: Davidson, 2006**

| Domain | Risk of Bias Rating | Explanation |
| --- | --- | --- |
| Selection Bias | Probably high risk of bias | There is clear information on cohort attrition, although recruitment process, and inclusion/exclusion; however, differences between the participants and non-participants is unclear. Despite some of this information being present in Marsh et al., 1995, there is not enough evidence to permit a probably low risk of bias rating. |
| Blinding | Low risk of bias | As supported by the following text: “All examiners were blinded to MeHg exposure levels and to test results at previous ages" |
| Exposure Assessment | Probably low risk of bias | Mercury was measured in maternal hair. There is indirect evidence, which suggests that the methods were robust, (see Cernichiari et al., 1995; Davidson et al, 1995; Davidson et al, 1998; Myers et al, 1995; Myers et al, 2004). |
| Outcome Assessment | Probably low risk of bias | Outcomes were assessed and defined consistently across all participants, using the MSCA and the WISC-III, however, the outcome assessments were translated into Creole |
| Confounding | Probably high risk of bias | The study appropriately considered most of the important confounders (Tier I), but did not account for multiple other potentially important confounders (Tier II). Confounders considered included gender, maternal age, birth weight (kg), the child's medical history, alcohol consumption during pregnancy, the child's hearing status, HOME scores, caregiver intelligence, SES, FRS, HELPS |
| Incomplete Outcome Data | Low risk of bias | Reasons for missing outcome data are unlikely to be related to true outcome. To note, there is minimal missing outcome data (91% of the original cohort) |
| Selective Outcome Reporting | Low risk of bias | All of the study’s pre‐specified outcomes outlined in the methods that are of interest have been reported in the pre‐specified way. |
| Conflict of Interest | Low risk of bias | This research was supported by government grants. |
| Other | Probably low risk of bias | Potential for type 1 error. |

**Table 8: Davidson, 1998**

| Domain | Risk of Bias Rating | Explanation |
| --- | --- | --- |
| Selection Bias | Probably low risk of bias | Information about the original cohort in Shamlaye et al., 1995. It is unclear whether there are differences between those who participated and those who didn’t (although only a small number were lost to follow-up). |
| Blinding | Low risk of bias | As supposed by the followed text, “A team of specially trained Seychellois nurses blinded to MeHg levels administered the tests” |
| Exposure Assessment | Probably low risk of bias | Mercury was measured in maternal hair. There is insufficient information about the QA/QC methods to permit a judgment of low risk of bias, but there is indirect evidence that suggests that methods were robust (Cernichiari et al., 1995) |
| Outcome Assessment | Probably low risk of bias | Outcomes were assessed and defined consistently across all participants, using the MSCA, however, the outcome assessments were translated into Creole |
| Confounding | Probably low risk of bias | The study appropriately considered most of the important confounders (Tier I), and other potentially important confounders (Tier II). Confounders considered include birth weight, birth order, sex, history of breastfeeding, hearing status, and the child’s medical history, maternal age, maternal smoking, maternal alcohol consumption, maternal medical history, caregiver intelligence, language spoken in the home, SES, HOME score and PCBs. |
| Incomplete Outcome Data | Low risk of bias | Reasons for missing outcome data are unlikely to be related to true outcome. To note, there is minimal missing outcome data (91% of the original cohort) |
| Selective Outcome Reporting | Low risk of bias | All of the study’s pre‐specified outcomes outlined in the methods that are of interest have been reported in the pre‐specified way. |
| Conflict of Interest | Low risk of bias | This research was supported by government grants. |
| Other | Low risk of bias | The study appears to be free of other sources of bias |

**Table 9: Davidson, 2004**

| Domain | Risk of Bias Rating | Explanation |
| --- | --- | --- |
| Selection Bias | Probably low risk of bias | There is insufficient information about participant selection to permit a judgment of low risk of bias. However, there is indirect evidence which suggests that inclusion/exclusion criteria, recruitment process and enrollment procedures, as well as participation and follow-up rates were consistent across groups as described by the criteria for a judgment of low risk of bias (see Shamlaye et al., 1995 and Marsh et al., 1995). |
| Blinding | Low risk of bias | As supported by the text: “All examiners and family members were blinded to MeHg exposure levels and to previous testing results" |
| Exposure Assessment | Probably low risk of bias | There may be insufficient information about the exposure assessment methods to permit a judgement of low risk of bias. However, there is indirect evidence, which suggests that methods were robust, as described by the criteria for a judgement of low risk of bias ( see Cernichiari et al., 1995). |
| Outcome Assessment | Probably low risk of bias | Outcomes were assessed and defined consistently across all study participants, using valid and reliable measures of intelligence (MSCA). Although, it is important to note that all tests were translated into Creole |
| Confounding | Probably high risk of bias | The study appropriately considered most of the important confounders (Tier I) but did not account for multiple other potentially important confounders (Tier II). Confounders considered include gender, maternal intelligence, HOME scores, SES and HFFI, and recent postnatal exposure. |
| Incomplete Outcome Data | Low risk of bias | Reasons for missing outcome data are unlikely to be related to true outcome. To note, there is minimal missing outcome data (91% of the original cohort) |
| Selective Outcome Reporting | Low risk of bias | All of the study’s pre-specified outcomes outlined in the protocol, methods, abstract, and/or introduction that are of interest have been reported in the pre-specified way. |
| Conflict of Interest | Low risk of bias | This research was supported by government grants |
| Other | Low risk of bias | The study appears to be free of other sources of bias. |

**Table 10: Ernhart, 1989**

| Domain | Risk of Bias Rating | Explanation |
| --- | --- | --- |
| Selection Bias | Low risk of bias | The descriptions of the source population, inclusion/exclusion criteria, recruitment process and enrolment procedures, as well as participation and follow-up rates, were sufficiently detailed. Cases lost to follow-up were contrasted with those receiving the 4- and 10-month assessments on 40 variables collected at or before birth. None of these contrasts were significant. |
| Blinding | Low risk of bias | As supported by the following text: “All examinations were conducted by well-trained examiners who were blinded to risk and other background information" |
| Exposure Assessment | Probably low risk of bias | There may be insufficient information about the exposure assessment methods to permit a judgement of low risk of bias. However, indirect evidence suggests that methods were robust, as described by the criteria for a judgement of low risk of bias (details in Ernhart et al., 1985). |
| Outcome Assessment | Low risk of bias | Outcomes were assessed and defined consistently across all study participants, using valid and reliable measures of intelligence (i.e., WPPSI). However, two children were assessed using the Standford-Binet Intelligence Scale. “The validity of the S-B to provide a downward extension for these children is indicated by a correlation of .74 for the rest of the sample between the scores at age three years on the S-B and the WPPSI Full Scale IQ at age 4-10.” |
| Confounding | Probably low risk of bias | The study evaluated most of the important confounders (Tier I) and some of the other potentially important confounders (Tier II) Confounders considered include: sex, race, birth order, birth weight, gestational age at birth, parental education, maternal IQ, alcohol, smoking, HOME score |
| Incomplete Outcome Data | Low risk of bias | The reasons for missing outcome data are unlikely to be related to the true outcome. “Of the cases lost, 38% refused further participation, 38% moved, 19% could not be located and 5% died, were adopted, or suffered a severe medical condition.” |
| Selective Outcome Reporting | Low risk of bias | All of the study’s pre-specified outcomes outlined in the protocol, methods, abstract, and/or introduction that are of interest have been reported in the pre-specified way. |
| Conflict of Interest | Low risk of bias | Funding source is limited to government, non-profit organizations, or academic grants funded by government, foundations and/or non-profit organizations |
| Other | Low risk of bias | The study appears to be free of other sources of bias. |

**Table 11: Factor-Litvak, 2014**

| Domain | Risk of Bias Rating | Explanation |
| --- | --- | --- |
| Selection Bias | Low risk of bias | The descriptions of the source population, inclusion/exclusion criteria, recruitment process and enrollment procedures, as well as participation and follow-up rates were sufficiently detailed. Adequate data were supplied on the distribution of relevant study sample and population characteristics to support the assertion that risk of selection effects was minimal. |
| Blinding | Probably low risk of bias | The review authors judge that the outcome and exposure measures are not likely to be influenced by a lack of blinding. The exposure was measured by a separate entity (CDC) |
| Exposure Assessment | Probably low risk of bias | Phthalates were measured in one urine sample. There may be insufficient information about the exposure assessment methods to permit a judgement of low risk of bias. However, indirect evidence suggests that the methods were robust (i.e., silva et al., 2004). |
| Outcome Assessment | Low risk of bias | Outcomes were assessed and defined consistently across all study participants, using valid and reliable measures (WISC-IV) |
| Confounding | Low risk of bias | The study appropriately considered all important confounders (Tier I), and most other potentially important confounders (Tier II). Confounders considered include race/ethnicity, maternal education, marital status, household income, parity, gestational age, birth weight, sex, breastfeeding history, exposure to tobacco smoke, prenatal alcohol consumption, prenatal psychosocial factors, maternal demoralization, maternal IQ, and HOME score. The study also considered other neurotoxicants in sensitivity analyses |
| Incomplete Outcome Data | Low risk of bias | Any missing data have been imputed using appropriate methods |
| Selective Outcome Reporting | Low risk of bias | All of the study’s pre-specified outcomes outlined in the protocol, methods, abstract, and/or introduction that are of interest have been reported in the pre-specified way. |
| Conflict of Interest | Low risk of bias | Funding source is limited to government, non-profit organizations, or academic grants funded by government, foundations and/or non-profit organizations |
| Other | Low risk of bias | The study appears to be free of other sources of bias. |

**Table 12: Freire, 2018**

| Domain | Risk of Bias Rating | Explanation |
| --- | --- | --- |
| Selection Bias | Probably high risk of bias | Indirect evidence suggests inclusion/exclusion criteria, recruitment/enrollment procedures, and participation/follow-up rates were inconsistent across groups. The text indicates differences between cohorts included in the study (i.e., the Granada cohort contained only boys). Additionally, recruitment procedures differed between cohorts (i.e., the Grenada cohort recruited at birth and the other cohorts recruited at first prenatal visit). Indirect evidence (Guxens et al., 2012) indicates loss of study participants (up to age 1-1.5) was not random. |
| Blinding | Low risk of bias | As supported by the following text: “The MSCA was administered by trained psychometricians who were blinded to the metal exposure of the children." |
| Exposure Assessment | Probably low risk of bias | Lead and mercury were measured in placental tissue. There may be insufficient information about the exposure assessment methods to permit a judgement of low risk of bias. However, indirect evidence suggests that the methods were robust (see Amaya et al., 2013; Gil et al., 2006, 2011; Olmedo et al., 2010.) |
| Outcome Assessment | Low risk of bias | Intelligence was assessed and defined consistently across all study participants, using valid and reliable measures (MSCA) |
| Confounding | Probably high risk of bias | The study evaluated some of the important confounders (Tier I) and some of the other potentially important confounders relevant (Tier II). Confounders that were considered include maternal age, schooling, pre-BMI, maternal smoking, maternal employment, parental social class, gestational age, gender, birth weight/length, delivery type, SGA, breastfeeding, number of siblings, place of residence (urban/rural), fish intake, vitamin D, and psychologist administering the test, as well as the child’s age at assessment. |
| Incomplete Outcome Data | Probably high risk of bias | 63% of 3294 babies born underwent neuropsychological testing. There are unclear reasons for missing data. |
| Selective Outcome Reporting | High risk of bias | The authors mention that they evaluated the interaction between metals and the effect modification by gender, breastfeeding, and smoking during pregnancy with respect to the general cognitive score, using interaction terms and stratified linear regression models. Nonetheless, they didn’t report the outcomes of the metal x sex interaction effect for Pb, Mn, and Cd. |
| Conflict of Interest | Low risk of bias | Study authors make a claim denying conflicts of interest |
| Other | Probably low risk of bias | “Multiple comparisons were conducted, and thus cannot rule out the possibility that some associations were due to chance; however, it appears unlikely that the significant trends found for Mn and Hg are the result of chance” |

**Table 13: Furlong, 2017**

| Domain | Risk of Bias Rating | Explanation |
| --- | --- | --- |
| Selection Bias | Probably low risk of bias | There is insufficient information about participant selection to permit a judgment of high risk of bias, however, there is indirect evidence which suggests that inclusion/exclusion criteria, recruitment, and enrolment procedures, as well as participation and follow-up rates were consistent across groups, as described by the criteria for a judgment of low risk of bias (see Berkowitz et al., 2003, Engel et al., 2007). |
| Blinding | Probably low risk of bias | The review authors judge that the outcome measures, as well as the exposure measures, are not likely to be influenced by lack of blinding. Exposure was measured in a lab at the CDC and the outcome was measured by a separate entity. |
| Exposure Assessment | Probably high risk of bias | OP Pesticides and phthalates were measured using single-spot urine samples. Quality control and laboratory methods are described elsewhere, however does not include enough information to permit a probably low risk of bias (Barr et al., 2005; Bravo et al., 2004) |
| Outcome Assessment | Low risk of bias | Outcomes were assessed and defined consistently across all study participants, using valid and reliable measures (WPPSI-III & WISC-IV) |
| Confounding | Probably low risk of bias | The study appropriately considered most of the important confounders (Tier I) and all other potentially important confounders (Tier II). Confounders considered include maternal education, race, marital status, maternal age, HOME scores, smoking, alcohol, Spanish language, analysis batch, creatinine, examiner, coexposures |
| Incomplete Outcome Data | Low risk of bias | Missing data have been imputed using appropriate methods |
| Selective Outcome Reporting | Low risk of bias | All of the study’s pre-specified outcomes outlined in the protocol, methods, abstract, and/or introduction that are of interest have been reported in the pre-specified way. |
| Conflict of Interest | Low risk of bias | Funding source is limited to government, non-profit organizations, or academic grants funded by government, foundations and/or non-profit organizations |
| Other | Low risk of bias | The study appears to be free of other sources of bias. |

**Table 14: Golding, 2017**

| Domain | Risk of Bias Rating | Explanation |
| --- | --- | --- |
| Selection Bias | Probably low risk of bias | Cohort and recruitment details are seen in Boyd et al 2013. No biases regarding a range of social conditions and lifestyles were noted. However, older and/or more educated women were more likely to have had blood taken for trace metal analyses. |
| Blinding | Probably low risk of bias | The review authors judge that the outcome measures, as well as the exposure measures, are not likely to be influenced by lack of blinding. |
| Exposure Assessment | Probably low risk of bias | There may be insufficient information about the exposure assessment methods to permit a judgment of low risk of bias. However, there is indirect evidence, which suggests that methods were robust (see Golding et al., 2013). It is important to note, however, that whole blood had been stored for 18+ years before analyzing mercury. Even so, the authors suggest that this is unlikely to be a problem. |
| Outcome Assessment | Probably low risk of bias | Outcome was assessed using the short-form WISC-III described elsewhere in Bath et al., 2013. |
| Confounding | Probably high risk of bias | The study did not account for multiple important confounders (Tier I) but did account for most other potentially important confounders relevant (Tier II). Confounders considered include family adversity, smoking in pregnancy, alcohol consumption in pregnancy, maternal age, parity, maternal education, child age and sex, breastfeeding, and fish intake. |
| Incomplete Outcome Data | High risk of bias | Missing data was not considered at random, and so was not included in the analysis. |
| Selective Outcome Reporting | Low risk of bias | All of the study’s pre‐specified outcomes outlined in the methods that are of interest have been reported in a pre‐specified way. |
| Conflict of Interest | Low risk of bias | The funders had no involvement in the study design nor in the collection, analysis, and interpretation of the data. The researchers worked independently from the funders. |
| Other | Low risk of bias | The study appears to be free of other sources of bias |

**Table 15: Huang, 2015**

| Domain | Risk of Bias Rating | Explanation |
| --- | --- | --- |
| Selection Bias | Low risk of bias | The descriptions of the source population, inclusion/exclusion criteria, recruitment process and enrollment procedures, as well as participation and follow-up rates were sufficiently detailed. Adequate data were supplied on the distribution of relevant study sample and population characteristics to support the assertion that risk of selection effects was minimal. |
| Blinding | Low risk of bias | As supported by the following text: “A total of 10 certified psychologists who were unaware of the results of the phthalate measurements administered all tests." |
| Exposure Assessment | Probably low risk of bias | Phthalates were measured in one spot urine sample and adjusted for creatinine. There is indirect evidence, which suggests that methods were robust, as described by the criteria for a judgement of low risk of bias (See Lin et al., 2011). |
| Outcome Assessment | Low risk of bias | Outcomes were assessed and defined consistently across all study participants, using valid and reliable measures of intelligence (WPPSI-III, WISC-III, WISC-IV) |
| Confounding | Probably high risk of bias | The study evaluated some of the important confounders (Tier I) and some of the other potentially important confounders (Tier II). Confounders considered include: gender, birth weight, child’s age, breastfeeding status, gestational age, child’s BMI, maternal age, education, parity, cigarette, alcohol, and HOME Scores. |
| Incomplete Outcome Data | Probably high risk of bias | There is insufficient information on incomplete outcome data |
| Selective Outcome Reporting | Low risk of bias | All of the study’s pre‐specified outcomes outlined in the methods that are of interest have been reported in the pre‐specified way. |
| Conflict of Interest | Low risk of bias | Funding source is limited to government, non-profit organizations, or academic grants funded by government, foundations and/or non-profit organizations |
| Other | Low risk of bias | The study appears to be free of other sources of bias. |

**Table 16: Huang, 2007**

| Domain | Risk of Bias Rating | Explanation |
| --- | --- | --- |
| Selection Bias | Probably low risk of bias | There is insufficient information about participant selection to permit a judgment of low risk of bias. However, there is indirect evidence which suggests that inclusion/exclusion criteria, recruitment and enrollment procedures, as well as participation and follow-up rates, were consistent across groups (see Davidson et al., 1998; Marsh et al., 1995b; Myers et al., 1995c, 1997, 2003; Shamlaye et al., 1995). |
| Blinding | Probably low risk of bias | There is insufficient information about blinding to permit a judgment of low risk of bias, however, there is indirect evidence which suggests the study was adequately blinded (see Davidson et al., 1998; Marsh et al., 1995b; Myers et al., 1995c, 1997, 2003; Shamlaye et al., 1995). |
| Exposure Assessment | Probably low risk of bias | There may be insufficient information about the exposure assessment methods to permit a judgement of low risk of bias, however, there is indirect evidence, which suggests that methods were robust, as described by the criteria for a judgement of low risk of bias (See Cernichiari et al., 1995). |
| Outcome Assessment | Low risk of bias | Outcomes were assessed and defined consistently across all study participants, using valid and reliable measures of intelligence (WISC III) |
| Confounding | Probably high risk of bias | The study appropriately considered most of the important confounders (Tier I), but did not account for multiple other potentially important confounders (Tier II) Confounders considered include: sex, maternal age, test examiner, maternal intelligence, medical history, FRS, family status, SES, HELPS, child age at testing, HOME Scores, hearing, and recent postnatal exposure |
| Incomplete Outcome Data | High risk of bias | “For this analysis, observations with missing information were ignored.” |
| Selective Outcome Reporting | Low risk of bias | All of the study’s pre-specified outcomes outlined in the protocol, methods, abstract, and/or introduction that are of interest have been reported in the pre-specified way. |
| Conflict of Interest | Low risk of bias | Funding source is limited to government, non-profit organizations, or academic grants funded by government, foundations and/or non-profit organizations |
| Other | Low risk of bias | The study appears to be free of other sources of bias. |

**Table 17: Ikeno, 2018**

| Domain | Risk of Bias Rating | Explanation |
| --- | --- | --- |
| Selection Bias | High risk of bias | There were indications from descriptions of the source population, inclusion/exclusion criteria, recruitment and enrollment procedures, as well as participation and follow-up rates that the risk of selection effects was substantial. “The annual income of non-participants and participants at 42 months of age was significantly different. The 42-month-old subjects had 5,000,000 yen compared to those in the non-participant group. Therefore, this study population was biased for economic advantage and environmental conditions.” |
| Blinding | Low risk of bias | As supported by the following text: “The examiners were unaware of the infants' exposure levels to DLCs" |
| Exposure Assessment | Probably low risk of bias | There may be insufficient information about the exposure assessment methods to permit a judgement of low risk of bias, but there is indirect evidence, which suggests that methods were robust, as described by the criteria for a judgement of low risk of bias. Blood sampling details from mothers and specimen storage have been previously described (Nakajima et al., 2006). |
| Outcome Assessment | Low risk of bias | Outcomes were assessed and defined consistently across all study participants, using valid and reliable measures (K-ABC) |
| Confounding | Probably high risk of bias | The study accounted for some of the important confounders (Tier I) and some of the other potentially important confounders (Tier II). Confounders considered include maternal age, smoking, gestational age birth weight, children's age, mothers’ WASI score, income, ICCE, and blood sampling |
| Incomplete Outcome Data | Probably high risk of bias | It is unclear how those with missing outcome data differ on exposure levels and whether this impacted the results. |
| Selective Outcome Reporting | Low risk of bias | All of the study’s pre-specified outcomes outlined in the protocol, methods, abstract, and/or introduction that are of interest have been reported in the pre-specified way. |
| Conflict of Interest | Low risk of bias | The authors declare no conflicts of interest. |
| Other | Low risk of bias | The study appears to be free of other sources of bias. |

**Table 18: Jacobson, 2002**

| Domain | Risk of Bias Rating | Explanation |
| --- | --- | --- |
| Selection Bias | High risk of bias | Although the descriptions of the source population, inclusion/exclusion criteria, recruitment and enrollment procedures, as well as participation and follow-up rates were sufficiently detailed, there was no support to indicate that potential selection effects were not differential across both exposure and outcome |
| Blinding | Probably low risk of bias | The review authors judge that the outcome and exposure measures are not likely to be influenced by lack of blinding. The exposure was measured by a separate entity to the outcome |
| Exposure Assessment | Probably low risk of bias | There may be insufficient information about the exposure assessment methods to permit a judgement of low risk of bias, however, there is indirect evidence, which suggests that methods were robust, as described by the criteria for a judgement of low risk of bias (See Needham et al., 1993). |
| Outcome Assessment | Low risk of bias | Outcomes were assessed and defined consistently across study participants, using valid and reliable measures (MSCA and WISC) |
| Confounding | Probably low risk of bias | The study appropriately considered most of the important confounders (Tier I), and other potentially important confounders (Tier II). Confounders considered include SES, maternal age, marital status, sex of the child, age of the child at testing, examiner, parity, gravidity, number of children, maternal education, maternal IQ, HOME scores, nursery school attendance, maternal employment, family stress, maternal drinking, maternal smoking, delivery complications, and child’s grades in school |
| Incomplete Outcome Data | Probably low risk of bias | “17 participants failed to cooperate with all or all but one of the items on one or more of the 17 McCarthy subtests. The data from one highly exposed child with an IQ score of 63, who had been diagnosed as mentally retarded, were excluded from the statistical analysis to avoid undue influence by extreme scores.” Only 7% of data on the MSCA was missing – minimal missing data is unlikely to be related to the true affect and impact the results. |
| Selective Outcome Reporting | Low risk of bias | All of the study’s pre-specified outcomes outlined in the protocol, methods, abstract, and/or introduction that are of interest have been reported in the pre-specified way. |
| Conflict of Interest | Low risk of bias | Funding source is limited to government, non-profit organizations, or academic grants funded by government, foundations and/or non-profit organizations |
| Other | Low risk of bias | The study appears to be free of other sources of bias |

**Table 19: Kyriklaki, 2016**

| Domain | Risk of Bias Rating | Explanation |
| --- | --- | --- |
| Selection Bias | Probably high risk of bias | There is detailed information on recruitment, inclusion, exclusion, and sub-sample. Insufficient data was supplied on the distribution of relevant population characteristics. The authors mention that "children included in the present analysis who had complete data were more socially advantaged than the remainder of the cohort and this could lead to underestimation of the observed associations” |
| Blinding | Probably low risk of bias | The review authors judge that the outcome and exposure measures are not likely to be influenced by lack of blinding. The exposure was measured by a separate entity from the outcomes. |
| Exposure Assessment | Probably low risk of bias | There may be insufficient information about the exposure assessment methods to permit a judgement of low risk of bias. However, there is indirect evidence, which suggests that methods were robust, as described by the criteria for a judgement of low risk of bias (See Koponen et al., 2013). |
| Outcome Assessment | Low risk of bias | Outcomes were assessed and defined consistently across all study participants, using valid and reliable measures (MSCA) |
| Confounding | High risk of bias | The study did not account for multiple important confounders (Tier I) and other potentially important confounders relevant (Tier II). Confounders considered include sex of the child, quality of the assessment, examiner, maternal age, maternal education, maternal tobacco smoke, parity, birth weight, preterm birth, breastfeeding duration, pre-BMI, TSH and maternal IQ. |
| Incomplete Outcome Data | Probably high risk of bias | There is insufficient information as how missing outcome data may have impacted the results. |
| Selective Outcome Reporting | Low risk of bias | All of the study’s pre‐specified outcomes outlined in the methods that are of interest have been reported in the pre‐specified way. |
| Conflict of Interest | Low risk of bias | The authors declare no conflict of interest |
| Other | Low risk of bias | The study appears to be free of other sources of bias |

**Table 20: McBride, 1982**

| Domain | Risk of Bias Rating | Explanation |
| --- | --- | --- |
| Selection Bias | High risk of bias | There was no support to indicate that potential selection effects were not differential across both exposure and outcome |
| Blinding | Probably low risk of bias | The review authors judge that the outcome and exposure measures are not likely to be influenced by lack of blinding. The exposure was measured by a separate entity than to the outcome. |
| Exposure Assessment | Probably high risk of bias | There is insufficient information about the exposure assessment methods to permit a judgement of low risk of bias and there is no indirect evidence, to suggests that methods were robust. |
| Outcome Assessment | Probably low risk of bias | Outcomes were assessed and defined consistently across all study participants, using valid and reliable measures of intelligence (PPVT). However, it is unclear who administered these tests. |
| Confounding | High risk of bias | This study did not account for any confounders. |
| Incomplete Outcome Data | Probably high risk of bias | There is insufficient information on incomplete outcome data |
| Selective Outcome Reporting | Low risk of bias | All of the study’s pre-specified outcomes outlined in the protocol, methods, abstract, and/or introduction that are of interest have been reported in the pre-specified way. |
| Conflict of Interest | High risk of bias | As supported by the text: "This study was jointly financed by the Australian lead development association and the international lead zinc research organization, both of which are affiliated with the lead industry" |
| Other | Low risk of bias | The study appears to be free of other sources of bias |

**Table 21: McMichael, 1988**

| Domain | Risk of Bias Rating | Explanation |
| --- | --- | --- |
| Selection Bias | High risk of bias | The descriptions of the source population, inclusion/exclusion criteria, recruitment and enrollment procedures, as well as participation and follow-up rates were sufficiently detailed, however, there was no support to indicate that potential selection effects were not differential across both exposure and outcome |
| Blinding | Low risk of bias | “A full-time research psychologist who was blinded to the child's past or current blood lead concentration, conducted all testing sessions within a clinical setting. Although the psychologist had also assessed the child's development (using the Bayley Scales of Infant Development) at the age of two years, they were not aware of that earlier result when making the subsequent assessment” |
| Exposure Assessment | Probably low risk of bias | There may be insufficient information about the exposure assessment methods to permit a judgement of low risk of bias but there is indirect evidence, which suggests that methods were robust, as described by the criteria for a judgement of low risk of bias (See reference #18). |
| Outcome Assessment | Probably low risk of bias | Outcomes were assessed and defined consistently across all study participants, using valid and reliable measures (i.e., MSCA). There is no Australian standardization data on the MSCA, so they did not evaluate absolute scores, rather they only evaluated relative scores for compared groups of children. |
| Confounding | Probably high risk of bias | The study evaluated some of the important confounders (Tier I), and some of the other potentially important confounders (Tier II). Confounders considered include sex, residence, mother's education, mother's work site, marital status, maternal age, antenatal medication use, mother's IQ, HOME scores, father's education, delivery, Apgar score, oxygen use at birth, neonatal jaundice, birth weight, and birth rank. |
| Incomplete Outcome Data | Probably high risk of bias | The majority (80%) of the children who were lost to follow-up during the four years of postnatal study belonged to families that left the Port Pirie district. A few families simply discontinued their participation. Unclear how the remaining 20% of children lost to follow up impact the results. |
| Selective Outcome Reporting | Low risk of bias | All of the study’s pre‐specified outcomes outlined in the methods that are of interest have been reported in the pre‐specified way. |
| Conflict of Interest | Low risk of bias | The study was supported by a series of grants from the National Health and Medical Research Council as well as additional assistance from the South Australian Health Commission and the University of Adelaide. |
| Other | Low risk of bias | The study appears to be free of other sources of bias |

**Table 22: McMichael, 1994**

| Domain | Risk of Bias Rating | Explanation |
| --- | --- | --- |
| Selection Bias | High risk of bias | There is insufficient information about participant selection to permit a judgment of low risk of bias, but there is indirect evidence which suggests that inclusion/exclusion criteria, recruitment and enrollment procedures, as well as participation and follow-up rates were consistent across groups as described by the criteria for a judgment of low risk of bias (See Wigg et al., 1988; McMichael et al., 1988; and Baghurst et al., 1992). However, those include differ from those excluded, as supported by the following text: "The demographic characteristics of the 262 children from whom teeth were obtained showed some differences from those of other children in the cohort. Teeth were obtained from a higher proportion of females than males, and the children who donated teeth tended to come from families with higher socioeconomic status, higher quality of home environment, and lower prevalence of parental smoking.” |
| Blinding | Low risk of bias | As supported by the following text: “All assessments were performed…by the study's full-time psychologist, who was unaware of each child's lead-exposure status." |
| Exposure Assessment | Probably low risk of bias | Whole teeth were used rather than circumpulpal dentin. Nonetheless, QA/QC for methods are described and are satisfactory |
| Outcome Assessment | Low risk of bias | Outcomes were assessed and defined consistently across all study participants, using valid and reliable measures (WISC-R) |
| Confounding | Probably low risk of bias | The study appropriately considered most of the important confounders (Tier I), and some of the other potentially important confounders (Tier II) Confounders considered include sex, birth weight, birth order, feeding style, duration of breastfeeding, parental smoking, mother’s age, period of residence in port Pirie, SES, HOME scores, maternal IQ, father's education, whether the child's parents were living together, and the age the tooth was shed. |
| Incomplete Outcome Data | Probably high risk of bias | There is unclear information on missing outcome data. |
| Selective Outcome Reporting | Low risk of bias | All of the study’s pre-specified outcomes outlined in the protocol, methods, abstract, and/or introduction that are of interest have been reported in the pre-specified way. |
| Conflict of Interest | Low risk of bias | This research was supported by a series of grants from the National Health and Medical Research Council with additional assistance from the South Australian Health Commission and the University of Adelaide. |
| Other | Probably low risk of bias | As supported by the following text: "The power to detect a sex difference among the subset of children who donated a tooth would have been substantially reduced.” |

**Table 23: Min, 2009**

| Domain | Risk of Bias Rating | Explanation |
| --- | --- | --- |
| Selection Bias | Probably low risk of bias | The descriptions of the source population, inclusion/exclusion criteria, recruitment and enrollment procedures, as well as participation, and follow-up rates were sufficiently detailed. Although retention rates are generally high (>90%), a greater percentage of African American and married women consented to blood collection, with a lower percentage of foster parents consenting to blood collection. |
| Blinding | Low risk of bias | As supported by the following text: “Examiners were unaware of lead and prenatal drug exposures, including cocaine" |
| Exposure Assessment | Probably low risk of bias | Lead was measured in venous blood. There may be insufficient information about the exposure assessment methods to permit a judgement of low risk of bias but there is indirect evidence, which suggests that methods were robust, as described by the criteria for a judgement of low risk of bias (i.e., the lab was enrolled in the CDC proficiency testing program for blood lead and was OSHA approved for blood lead analysis) |
| Outcome Assessment | Low risk of bias | Outcomes were assessed and defined consistently across all study participants, using valid and reliable measures (abbreviated WPPSI-R at 4 years and WISC-IV at 9 and 11 years) |
| Confounding | Low risk of bias | The study appropriately considered all important confounders (Tier I) and most other potentially important confounders (Tier II). Covariates considered include SES, maternal age, marital status at birth, years of education of biological mother, number of prenatal care visits, parity, child’s race and gender, infant's head circumference, maternal vocabulary and nonverbal IQ, Maternal psychological distress, HOME scores, Iron Deficiency Anemia (IDA), prenatal cocaine exposure, cigarettes, and alcohol |
| Incomplete Outcome Data | Low risk of bias | The proportion of missing outcome data at ages 9 and 11 is unlikely to have a relevant impact (96% retention rate). |
| Selective Outcome Reporting | Low risk of bias | All of the study’s pre‐specified outcomes outlined in the methods that are of interest have been reported in the pre‐specified way |
| Conflict of Interest | Low risk of bias | Study authors make a claim denying conflicts of interest |
| Other | Low risk of bias | The study appears to be free of other sources of bias. |

**Table 24: Myers, 1995**

| Domain | Risk of Bias Rating | Explanation |
| --- | --- | --- |
| Selection Bias | Probably low risk of bias | There is indirect information about the pilot cohort in Shamlaye et al, 1995. “A comparison of study participants (n = 217) with the remaining untested cohort children (n = 572) indicated that the two groups were similar about all covariates (including MeHg exposure) except maternal alcohol consumption during pregnancy. Only 5% of tested children’s mothers reported consuming alcohol during pregnancy, compared to 12% of the remaining cohort mothers” |
| Blinding | Probably low risk of bias | There is indirect evidence that those who administered the tests were blinded to MeHg levels (see Davidson et al., 1994). |
| Exposure Assessment | Probably low risk of bias | There is insufficient information about the exposure methods to permit a judgment of low risk of bias, but there is indirect evidence that suggests that the methods were robust (See Cernichiari et al., 1995; Myers et al 1995) |
| Outcome Assessment | Probably low risk of bias | There is insufficient information about the outcome assessment methods to permit a judgment of low risk of bias, but there is indirect evidence which suggests that methods were robust, as described by the criteria for a judgment of low risk of bias. However it is important to note that he MSCA was modified, translated into creole and standardized on a western population. These modifications and validity of them are described in Davidson et al, 1994. |
| Confounding | High risk of bias | The study did not account any of the important confounders (Tier I), but did account for some of the other potentially important confounders relevant (Tier-II). Confounders considered include birth weight, maternal age, gender, child's medical history, maternal alcohol and tobacco use during pregnancy, maternal medical history, number of persons per room living at the child's home, and one and five-minute APGAR scores. |
| Incomplete Outcome Data | Low risk of bias | The study authors comment on having no knowledge of any systematic reason for test scores to be missing. The mean mercury level for children not completing the GCI was 8.1 ppm, compared to 8.3 ppm for children who completed the test. |
| Selective Outcome Reporting | Low risk of bias | All of the study’s pre‐specified outcomes outlined in the methods that are of interest have been reported in the pre‐specified way. |
| Conflict of Interest | Low risk of bias | This study was supported by grants #ES-05197 and ES-01247 from the National Institute of Environmental Health Sciences (NIEHS) and from the U.S. Food and Drug administration via an interagency agreement with NIEHS. |
| Other | Probably low risk of bias | Potential for Type II error. |

**Table 25: Myers, 2003**

| Domain | Risk of Bias Rating | Explanation |
| --- | --- | --- |
| Selection Bias | Probably low risk of bias | There is insufficient information about participant selection to permit a judgment of low risk of bias, but there is indirect evidence which suggests that inclusion/exclusion criteria, recruitment and enrollment procedures, as well as participation and follow-up rates were consistent across groups as described by the criteria for a judgment of low risk of bias (see Myers et al., 1995; Davidson et al., 1998; Davidson et al., 1995) |
| Blinding | Low risk of bias | As supported by the following text: “All personnel working in Seychelles were unaware of theMeHg exposure from the start of the study and no individual MeHg concentrations have been shared with families, clinical investigators, or anyone in Seychelles" |
| Exposure Assessment | Probably low risk of bias | There may be insufficient information about the exposure assessment methods to permit a judgement of low risk of bias but there is indirect evidence, which suggests that methods were robust, as described by the criteria for a judgement of low risk of bias (i.e., Cernichiari et al., 1995) |
| Outcome Assessment | Low risk of bias | Outcomes were assessed and defined consistently across all study participants, using valid and reliable measures (i.e., WISC-III) |
| Confounding | Probably high risk of bias | The study appropriately considered most of the important confounders (Tier I), but did not account for multiple other potentially important confounders relevant (Tier II). Confounders considered include: sex, examiner, family resource scale, family status code, HELPS, child’s age, child medical history, maternal age, HOME scores, maternal IQ, SES, hearing, and child mercury concentration. |
| Incomplete Outcome Data | Probably low risk of bias | The reasons children were not tested included residing abroad, refusal, and inability to locate them. These are unlikely to be related to the true outcome. |
| Selective Outcome Reporting | Low risk of bias | All of the study’s pre-specified outcomes outlined in the protocol, methods, abstract, and/or introduction that are of interest have been reported in the pre-specified way. |
| Conflict of Interest | Low risk of bias | Study authors make a claim denying conflicts of interest. |
| Other | Low risk of bias | The study appears to be free of other sources of bias. |

**Table 26: Palumbo, 2000**

| Domain | Risk of Bias Rating | Explanation |
| --- | --- | --- |
| Selection Bias | Probably low risk of bias | There is insufficient information about participant selection to permit a judgment of low risk of bias, but there is indirect evidence which suggests that inclusion/exclusion criteria, recruitment and enrollment procedures, as well as participation and follow-up rates were consistent across groups as described by the criteria for a judgment of low risk of bias (See Shamlaye et al., 1995; Marsh et al., 1995) |
| Blinding | Low risk of bias | As supported by the following text: “The team members were blinded to maternal hair MeHg levels and to the results of testing that took place during previous visits." |
| Exposure Assessment | Probably low risk of bias | There may be insufficient information about the exposure assessment methods to permit a judgement of low risk of bias but there is indirect evidence, which suggests that methods were robust, as described by the criteria for a judgement of low risk of bias (see Cernichiari et al., 1995) |
| Outcome Assessment | Low risk of bias | Outcomes were assessed and defined consistently across all study participants, using valid and reliable measures (MSCA) |
| Confounding | Probably low risk of bias | The study appropriately considered most of the important confounders (Tier I) and some of the other potentially important confounders (Tier II). Covariates considered include birthweight, birth order, gender, history of breastfeeding, hearing status, child’s medical history, maternal age, smoking, alcohol, maternal medical history, intelligence, SES, HOME scores, and language spoken. |
| Incomplete Outcome Data | Low risk of bias | Minimal missing data. |
| Selective Outcome Reporting | Low risk of bias | All of the study’s pre-specified outcomes outlined in the protocol, methods, abstract, and/or introduction that are of interest have been reported in the pre-specified way. |
| Conflict of Interest | Probably low risk of bias | There is insufficient information to permit a judgment of low risk of bias, but there is indirect evidence which suggests the study was free of support from a company, study author, or other entities having a financial interest in the outcome of the study, as described by the criteria for a judgment of low risk of bias (i.e., other studies published within this cohort). |
| Other | Low risk of bias | The study appears to be free of other sources of bias. |

**Table 27: Rauh, 2011**

| Domain | Risk of Bias Rating | Explanation |
| --- | --- | --- |
| Selection Bias | Low risk of bias | The descriptions of the source population, inclusion/exclusion criteria, recruitment and enrollment procedures, participation and follow-up rates were sufficiently detailed, and adequate data were supplied on the distribution of relevant study sample and population characteristics to support the assertion that risk of selection effects was minimal |
| Blinding | Probably low risk of bias | The review authors judge that the outcome and exposure measures are not likely to be influenced by lack of blinding. The exposure was measured by a separate entity than to the outcome. |
| Exposure Assessment | Probably low risk of bias | There may be insufficient information about the exposure assessment methods to permit a judgement of low risk of bias but there is indirect evidence, which suggests that methods were robust, as described by the criteria for a judgement of low risk of bias. Methods for the laboratory assay for CPF, including quality control, reproducibility, and limits of detection (LODs), have also been previously published (Barr etal. 2002). In addition, OPPs were measured using cord blood - not urine. |
| Outcome Assessment | Low risk of bias | Outcomes were assessed and defined consistently across all study participants, using valid and reliable measures (WISC-IV) |
| Confounding | Low risk of bias | The study appropriately considered all important confounders (Tier I), but not necessarily all other potentially important confounders relevant (Tier II). Confounders included: child sex, race/ethnicity, maternal IQ, maternal education, income, child age at testing, environmental tobacco smoke and air pollution. |
| Incomplete Outcome Data | Probably low risk of bias | “The retention rate for the full cohort was 82% at the 7-year follow-up, with no significant sociodemographic differences between subjects retained in the study and those lost to follow-up” |
| Selective Outcome Reporting | Low risk of bias | All of the study’s pre-specified outcomes outlined in the protocol, methods, abstract, and/or introduction that are of interest have been reported in the pre-specified way. |
| Conflict of Interest | Low risk of bias | The authors declare they have no competing financial interests. |
| Other | Low risk of bias | The study appears to be free of other sources of bias. |

**Table 28: Ris, 2004**

| Domain | Risk of Bias Rating | Explanation |
| --- | --- | --- |
| Selection Bias | Low risk of bias | The descriptions of the source population, inclusion/exclusion criteria, recruitment and enrollment procedures, as well as participation and follow‐up rates were sufficiently detailed, and adequate data were supplied on the distribution of relevant study sample and population characteristics to support the assertion that risk of selection effects was minimal. |
| Blinding | Probably low risk of bias | The review authors judge that the outcome and exposure measures are not likely to be influenced by lack of blinding. The exposure was measured by a separate entity than to the outcome. |
| Exposure Assessment | Probably low risk of bias | There may be insufficient information about the exposure assessment methods to permit a judgment of low risk of bias but there is indirect evidence, which suggests that methods were robust, as described by the criteria for a judgment of low risk of bias. Clear descriptions of lead analyses can be found in Dietrich et al., 1987 and Dietrich et al., 1991. T |
| Outcome Assessment | Low risk of bias | Outcomes were assessed and defined consistently across all study participants, using valid and reliable measures (WRAT & WISC). |
| Confounding | Probably low risk of bias | The study appropriately considered most important confounders (Tier I), and some potentially important confounders (Tier II). Covariates considered include maternal age, Otitis media, birth weight, infections to 5 years of age, birth length, iron status, neonatal head circumference, consumption of alcohol, tobacco and marijuana during adolescence, gestational age by physical exam, gestational age by dates, APGAR at 1 and 5 minutes, subject’s gender, Obstetrical Complications Scale Score, subject’s age at the time of assessment, Postnatal Complications Scale score, SES, cigarette consumption during pregnancy, mean H.O.M.E. scores, alcohol consumption during pregnancy, marijuana consumption during pregnancy, maternal intelligence, use of narcotics during pregnancy, highest grade attained by a primary caregiver, number of previous abortions, family pr public assistance, number of previous stillbirths, number of adults in the home, gravidity, number of children in the home, parity, subject attended a preschool program |
| Incomplete Outcome Data | Low risk of bias | The reasons for missing outcome data are unlikely to be related to the true outcome. “Reasons for attrition since the last published follow-up assessment at 6.5 years (n = 253) included refusals (n = 4), chronically missed appointments (n = 6), inability to determine the current location of the subjects' families (n = 38), long-term incarceration (n = 4), homicide (n = 2), severe developmental disability (n = 2), and the lack of a psychometrician on the day of the appointment (n = 2). Subjects in the analysis did not differ significantly from those lost to follow-up in terms of measures of Pb exposure, perinatal health, early school-age intelligence, or socioeconomic status” |
| Selective Outcome Reporting | Probably low risk of bias | Not all of the study’s pre-specified primary outcomes have been reported. The authors wrote that for every factor, the PbB x Gender and PbB x SES interactions were tested. But they only reported statistically significant interaction effects. |
| Conflict of Interest | Low risk of bias | Funding source is limited to a government grant. |
| Other | Low risk of bias | The study appears to be free of other sources of bias. |

**Table 29: Schnaas, 2000**

| Domain | Risk of Bias Rating | Explanation |
| --- | --- | --- |
| Selection Bias | High risk of bias | There is insufficient information about participant selection to permit a judgment of low risk of bias, but there is indirect evidence which suggests that inclusion/exclusion criteria, recruitment and enrollment procedures, and participation and follow-up rates were consistent across groups as described by the criteria for a judgment of low risk of bias. (see Rothenberg et al., 1994). Although there were significant differences between those included and excluded on blood lead and GCI. |
| Blinding | Low risk of bias | As supported by the following: “Four trained psychologists who did not know the blood lead levels of their subjects applied the tests" |
| Exposure Assessment | Probably low risk of bias | here may be insufficient information about the exposure assessment methods to permit a judgment of low risk of bias but there is indirect evidence, which suggests that methods were robust, (see Rothenberg et al., 1994) |
| Outcome Assessment | High risk of bias | Intelligence was assessed and defined consistently across all study participants, however, since there are no norms for the MSCA in the Mexican population, they used the US norms to calculate the GCI with a Spanish translation of the test. |
| Confounding | Probably high risk of bias | The study appropriately considered most of the important confounders (Tier I) and did not account for multiple other potentially important confounders (Tier II). Confounders Included: maternal IQ, sex of the child, APGAR score at 5 minutes, birth weight, serial order of the child in the family, educational level of the mother, and family SES. |
| Incomplete Outcome Data | High risk of bias | “The statistical comparison of this group with the group with incomplete data indicated that the selected sample differed in important aspects from the unselected sample. The geometric mean of blood lead at 24 ± 36 months and at 42 ± 54 months was significantly higher in the excluded group. The GCI at 42, 48, and 54 months was also significantly lower in the excluded group.” |
| Selective Outcome Reporting | Low risk of bias | All of the study’s pre‐specified outcomes outlined in the methods that are of interest have been reported in a pre‐specified way. |
| Conflict of Interest | Low risk of bias | The funding source is limited to government grants. |
| Other | Probably low risk of bias | The authors constructed several separate multivariate models, one for each age range of blood lead level. This could have introduced some inflation of probabilities noted for lead effects due to multiple testing. |

**Table 30: Tatsuta, 2014**

| Domain | Risk of Bias Rating | Explanation |
| --- | --- | --- |
| Selection Bias | Probably low risk of bias | The descriptions of the source population, inclusion/exclusion criteria, recruitment and enrollment procedures, as well as participation and follow-up rates were sufficiently detailed, and adequate data were supplied on the distribution of relevant study sample characteristics. However, there is not enough data supplied on relevant population characteristics |
| Blinding | Probably low risk of bias | The review authors judge that the outcome measures, as well as the exposure measures, are not likely to be influenced by lack of blinding as the exposure was measured by a separate entity to the outcome |
| Exposure Assessment | Low risk of bias | There is high confidence in the accuracy of the exposure assessment methods, such as methods that have been tested for validity and reliability in measuring the targeted exposure; i.e., serum for PCBs and whole blood for mercury as well as lead. Appropriate QA/QC for methods are described and are satisfactory. |
| Outcome Assessment | Low risk of bias | Outcomes were assessed and defined consistently across all study participants, using valid and reliable measures (K-ABC) |
| Confounding | Probably low risk of bias | The study evaluated most of the important confounders (Tier I), and some of the other potentially important confounders relevant (Tier II). Confounders considered include: gender, birth order, drinking and smoking, breastfeeding, income, and maternal IQ. Note that this study was assessing Japanese children, so it is likely that the researchers did not believe it was necessary to report their participants' race/ethnicity explicitly. |
| Incomplete Outcome Data | Low risk of bias | There is only 1 participant with incomplete outcome data. |
| Selective Outcome Reporting | Low risk of bias | All of the study’s pre‐specified outcomes outlined in the methods that are of interest have been reported in the pre‐specified way. |
| Conflict of Interest | Low risk of bias | Financial supports were provided by the Japan Ministry of the Environment and the Japan Ministry of Health, Labor and Welfare |
| Other | Low risk of bias | The study appears to be free of other sources of bias. |

**Table 31: Vuong, 2017**

| Domain | Risk of Bias Rating | Explanation |
| --- | --- | --- |
| Selection Bias | Probably low risk of bias | Detailed information on enrollment, inclusion criteria and neurobehavioral assessments are described by Braun et al. (2017). |
| Blinding | Low risk of bias | As supported by the following text: “Neither HOME Study staff nor parents had knowledge of prenatal or childhood PBDE concentrations at the neurobehavioral assessment" |
| Exposure Assessment | Probably low risk of bias | The reviewers judge that there is a low risk of exposure misclassification. There is high confidence in the accuracy of the exposure assessment methods, such as methods that have been tested for validity and reliability in measuring the targeted exposure. PBDE's measured in serum and are lipid adjusted. QA/QC methods are also indirectly reported (see Jones et al., 2012) |
| Outcome Assessment | Low risk of bias | Outcomes were assessed and defined consistently across all study participants, using valid and reliable measures (WISC-IV) |
| Confounding | Probably low risk of bias | The study appropriately considered all important confounders (Tier I), and most other potentially important confounders (Tier II). Confounders considered include maternal age, race, income, cotinine, marital status, IQ, depression, HOME scores, sex of the child and child age, blood lead, and breastfeeding duration |
| Incomplete Outcome Data | Low risk of bias | Missing data have been imputed using appropriate methods |
| Selective Outcome Reporting | Low risk of bias | All of the study’s pre-specified outcomes outlined in the protocol, methods, abstract, and/or introduction that are of interest have been reported in the pre-specified way. |
| Conflict of Interest | Low risk of bias | The authors declare they have no competing financial interests |
| Other | Probably low risk of bias | “Multiple comparisons is also a concern; however, we used multiple informant models to incorporate all PBDE measures in the same model, thereby reducing the total number of models. Of the 24 models in the present study, we would expect to see one statistically significant association based on chance alone. However, we observed 10 statistically significant findings.” |

**Table 32: Zhu, 2020**

| Domain | Risk of Bias Rating | Explanation |
| --- | --- | --- |
| Selection Bias | Low risk of bias | The descriptions of the source population, inclusion/exclusion criteria, recruitment and enrollment procedures, as well as participation and follow-up rates were sufficiently detailed, and adequate data were supplied on the distribution of relevant study sample and population characteristics to support the assertion that risk of selection effects was minimal. |
| Blinding | Low risk of bias | “All of the raw data were submitted to a blinded researcher for calculation of the IQ scores of each participant" |
| Exposure Assessment | Probably low risk of bias | Prenatal phthalate exposure was measured in three spot urine samples, although they were adjusted for creatine rather than specific gravity. QA/QC was reported - discuss blanks, LoD, but there is additional information in previous studies (Gao et al 2017; Wang et al., 2013; Zhu et al., 2018) |
| Outcome Assessment | Low risk of bias | Outcomes were assessed and defined consistently across all study participants, using valid and reliable measures (C-WPPSI-IV) |
| Confounding | Probably high risk of bias | The study evaluated some of the important confounders (Tier I), and some of the other potentially important confounders relevant (Tier II). Confounders considered include maternal age, maternal IQ, pre BMI, parity, income, sunscreen use, pregnancy willingness, breastfeeding, and urinary creatinine concentration. |
| Incomplete Outcome Data | Low risk of bias | Missing data have been imputed using appropriate methods. |
| Selective Outcome Reporting | Low risk of bias | All of the study’s pre-specified outcomes outlined in the protocol, methods, abstract, and/or introduction that are of interest have been reported in the pre-specified way |
| Conflict of Interest | Low risk of bias | The authors declare that they have no known competing financial interests. |
| Other | Low risk of bias | The study appears to be free of other sources of bias. |
